# Development and validation of an automated basal cell carcinoma histopathology information extraction system using natural language processing

**DOI:** 10.3389/fsurg.2022.870494

**Published:** 2022-08-24

**Authors:** Stephen R. Ali, Huw Strafford, Thomas D. Dobbs, Beata Fonferko-Shadrach, Arron S. Lacey, William Owen Pickrell, Hayley A. Hutchings, Iain S. Whitaker

**Affiliations:** ^1^ Reconstructive Surgery and Regenerative Medicine Research Centre, Institute of Life Sciences, Swansea University Medical School, Swansea, United Kingdom; ^2^ Welsh Centre for Burns and Plastic Surgery, Morriston Hospital, Swansea, United Kingdom; ^3^ Neurology and Molecular Neuroscience Group, Institute of Life Science, Swansea University Medical School, Swansea University, Swansea, United Kingdom; ^4^ Health Data Research UK, Data Science Building, Swansea University Medical School, Swansea University, Swansea, United Kingdom; ^5^ Department of Neurology, Morriston Hospital, Swansea, United Kingdom; ^6^ Patient and Population Health and Informatics Research, Swansea University Medical School, Swansea, United Kingdom

**Keywords:** Natural Language Processing (NLP), information extraction (IE), basal cell carcinoma (BCC), electronic health records (EHRs), non-melanoma skin cancer (NMSC)

## Abstract

**Introduction:**

Routinely collected healthcare data are a powerful research resource, but often lack detailed disease-specific information that is collected in clinical free text such as histopathology reports. We aim to use natural Language Processing (NLP) techniques to extract detailed clinical and pathological information from histopathology reports to enrich routinely collected data.

**Methods:**

We used the general architecture for text engineering (GATE) framework to build an NLP information extraction system using rule-based techniques. During validation, we deployed our rule-based NLP pipeline on 200 previously unseen, de-identified and pseudonymised basal cell carcinoma (BCC) histopathological reports from Swansea Bay University Health Board, Wales, UK. The results of our algorithm were compared with gold standard human annotation by two independent and blinded expert clinicians involved in skin cancer care.

**Results:**

We identified 11,224 items of information with a mean precision, recall, and F1 score of 86.0% (95% CI: 75.1–96.9), 84.2% (95% CI: 72.8–96.1), and 84.5% (95% CI: 73.0–95.1), respectively. The difference between clinician annotator F1 scores was 7.9% in comparison with 15.5% between the NLP pipeline and the gold standard corpus. Cohen's Kappa score on annotated tokens was 0.85.

**Conclusion:**

Using an NLP rule-based approach for named entity recognition in BCC, we have been able to develop and validate a pipeline with a potential application in improving the quality of cancer registry data, supporting service planning, and enhancing the quality of routinely collected data for research.

## Introduction

Skin cancer is the most common malignancy in the UK, comprising at least 25% of all new cancer diagnoses (1, 2). Since the early 1990s, UK non-melanoma skin cancer (NMSC) incidence rates have increased by 163% and continue to rise, placing a huge demand on the National Health Service (NHS) (1). In order to plan and deliver this ever-increasing clinical activity, along with undertaking good quality research to improve patient outcomes, high quality and detailed data capture is required.

Routinely collected healthcare data have the potential to significantly impact skin oncology research, identifying new disease associations, treatment modalities, outcomes, and healthcare delivery planning (3, 4). Current coding methods and therefore data capture for NMSC grossly underestimate the true burden of disease, by up to 50% for basal cell carcinoma (BCC) and 30% for squamous cell carcinoma (SCC) (5). As per the UK and Ireland Association of Cancer Registries (UKIACR), most cancer registries currently record only a single BCC or SCC per patient lifetime (5). Metachronous, synchronous, and recurrent lesions are ignored in these data (5). Not only is a significant volume of data not collected, but the depth and quality of data are also lacking. Clinical coding using International Classification of Diseases Version 10 (ICD-10), Office of Population Censuses and Surveys Classification of Surgical Operations and Procedures Version 4 (OPCS-4), and other coding systems can misrepresent the true disease and treatment burden (6–8). Furthermore, the error can be introduced by those tasked with coding disease or treatment episodes (9). This practice of underestimating the true incidence of NMSC is not isolated to the UK alone (10). In order to gain a better understanding of the disease and the burden it places on both patients and the NHS, this needs to be addressed.

Rich data can be found locked away in “unstructured” formats such as clinic letters, handwritten clinical notes, and histopathology reports, which form a part of the electronic health record (EHR) (11). Manual review of the free text in EHRs has been the mainstay of data capture in this setting. This is a process that is labour-intensive, costly, and open to error and bias. Traditionally, EHR data have been inaccessible in an “unstructured” free text format; however, with the advent of Natural Language Processing (NLP), this is no longer the case.

Information extraction using NLP describes a set of techniques used to convert passages of written text into interpretable datasets through either rule-based or machine learning (ML) models (12). It can be used in healthcare for named entity recognition (NER) or feature extraction using unstructured EHR data. The stages can conceptually be broken down and summarised into five steps—text extraction, text processing, system task, performance evaluation, and implementation (13). First, an unstructured free text report is converted into a series of features such as part of speech tags, tokens, and phrase chunks, which are then algorithmically processed. The NLP algorithm can then be set to task. After the system is validated, it can finally be applied to unstructured text to extract data in the setting of its intended purpose. The nascent field of NLP has the potential to enrich routinely collected healthcare data with detailed disease-specific information and harness the power of big data to ensure that these data are accurate, comprehensive, and easily accessible.

The importance of big data and modern analytical techniques in medical research has been recognised by the United Kingdom (UK) Government in their Eight Great Technologies drive, by the Medical Research Council in 2016–2017, who plan to invest £37.5 million in health informatics over the next 5 years, and by The Royal College of Surgeons of England in their “Future of Surgery” commission (14–16). Additionally, the King's Fund report into dermatology services in the UK commissioned by the British Association of Dermatologists recognises the need for improved dermatology service data collection and accessible real-time information (17). The use of NLP in clinical outcomes research is accelerating. In a recent systematic review and meta-analysis of NLP-based data capture versus conventional administrative methods of data capture [current procedural terminology codes, international classification of diseases (ICD) codes, patient safety indicators based on discharge coding and diagnosis-related group database code], postoperative complications were identified with higher sensitivity, whilst specificity was comparable (18). NLP models may be reliably used for both confirming and ruling out documentation of outcomes/diagnoses, whilst conventional methods of data capture demonstrate clinical utility for confirming documentation of outcomes/diagnoses alone (18). Applications of NLP to the EHR continue to expand and include novel phenotype discovery, clinical trial screening, pharmacogenomics, drug-drug interaction and adverse drug event detection, and genome-wide and phenome-wide association studies (19).

We intend to build on this wave of enthusiasm and improve data collection and therefore research in skin oncology. In this study, we developed and internally validated a rule-based NLP pipeline to extract BCC primary histopathological data, with the ultimate aim of improving the quality of cancer registry data, supporting service planning, and enhancing the quality of research using routinely collected data.

## Materials and methods

### Study population

We used manually de-identified and pseudonymised BCC histopathology reports from Swansea Bay University Health Board. Forty-one histopathology reports from 2015 were used to develop, train, and test rule sets. This training corpus of 41 reports contained 62 individual BCCs. Training histopathology reports were written by ten consultant histopathologists. The gold standard validation corpus consisted of 200 histopathology reports from 2016 to 2018 and contained 299 individual BCCs. The validation histopathology reports were written by 20 consultant histopathologists.

### Annotation

Each free text histopathology report was annotated using the open-source, web-based annotation tool Markup (https://www.getmarkup.com/) (20). Markup incorporates NLP and active learning (AL) technologies to enable rapid and accurate annotation using custom user configurations, predictive annotation suggestions, and automated mapping suggestions to both domain-specific ontologies, such as the Unified Medical Language System (UMLS), and custom, user-defined ontologies.

### Annotation guidelines

We developed an annotation guideline (Supplementary Material 1) and data definition dictionary (Supplementary Material 2) as an aide for clinicians when annotating histopathology reports as the ground truth. An iterative approach to guideline development was taken, with a first draft containing general guidelines updated and re-tested following its implementation by two clinicians on an initial 20 histopathology reports and compared with a gold standard defined by consensus agreement between two expert skin cancer clinicians.

### Algorithm construction

**Figure 1:**
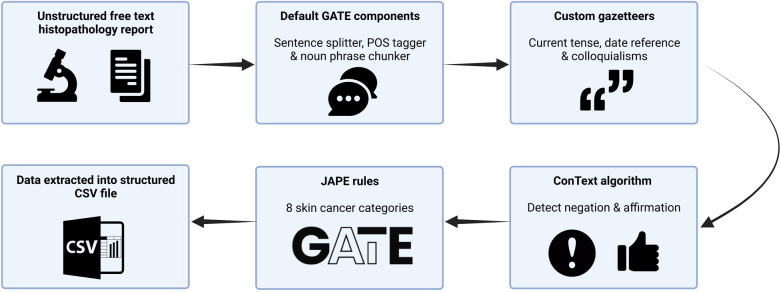
Schematic representation of our rule-based named entity recognition Natural Language Processing system for BCC histopathology reports. CSV, comma-separated values; JAPE, java annotation patterns engine scripting.

We used General Architecture for Text Engineering (GATE) Developer 9.0 (University of Sheffield, UK), an established open-source toolkit for NLP, to build an information extraction system using rule-based techniques from histopathology reports (21) (Figure 1). GATE can be defined as a Java-based infrastructure for developing and deploying software components that process human language (21). GATE as an architecture can be broken down into various types of components, known as resources (21). These resources include language resources (lexicons, corpora, or ontologies), processing resources (parsers, generators, or n-gram modellers), and visual resources (visualisation and editing components involved with graphical user interfaces) (21). The 139 entities that we set out to extract are summarised in Supplementary Table S1. We developed our own custom gazetteers (native dictionaries used within GATE) informed by the World Health Organisation classification of tumours of soft tissue and bone tumours, to map clinical terms to UMLS concepts (22). We deployed the ConText algorithm to detect the negation of extracted terms, e.g., “there was *no* residual disease,” and to detect affirmation of normal prognostic factors, such as “tumour *confined* to the dermis.” Finally, we used the Java Annotation Patterns Engine (JAPE) scripting language to define rules based on varying combinations of UMLS and custom lookups to extract eight broad information categories. In total, we created 80 separate gazetteers and 445 JAPE rule files in order to annotate the variables of interest, establish context, and to remove certain annotations from the output. Data were outputted into a comma-separated values (CSV) file by using the Groovy scripting language.

### Determining the number of documents needed for a gold standard validation corpus

There is no agreed standard for determining the size of a validation set in NLP (23–25). We therefore used string matching and a modified version of the method outlined by Juckett et al. to determine the number of documents required in our validation set to ensure that we represented all 139 concepts well enough to validate them (23). A further 1,000 manually de-identified and pseudonymised BCC histopathology reports generated from Swansea Bay University Health Board in 2015 were used for this. We calculated the capture probability of a token (which is any sequence of alphanumeric characters, beginning with a letter and occurring between spaces, slashes, brackets, braces, parentheses, quotation marks, or punctuation marks that are found in our 139 concepts) occurring in the validation set from these 1,000 reports (see Supplemental Material 3 for a full method). In order to achieve a capture probability of 90%, we required a validation set of 200 documents.

### Analysis and statistical tests

**Figure 2:**
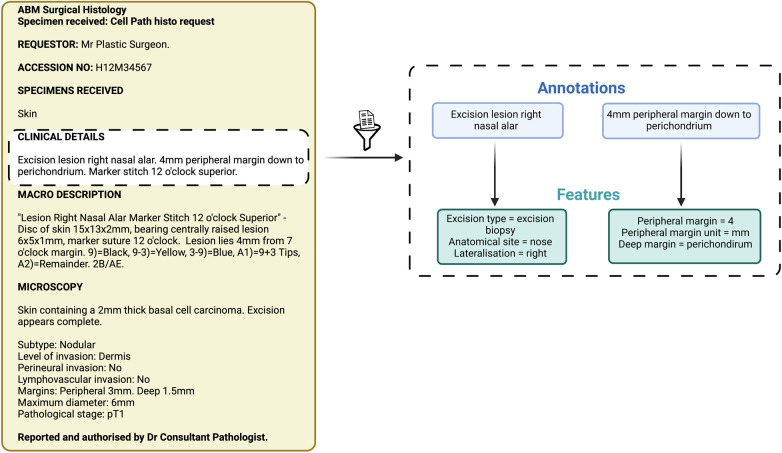
Schematic representation of annotations and features. These items of information were extracted by both the Natural Language Processing pipeline and the expert manual review.

Items of information extracted by the NLP pipeline were compared with those extracted by manual review performed by two independent expert skin cancer clinicians who had access to the annotation guidelines only (Figure 2). We used the most widely adopted measures in the literature to evaluate an NLP pipeline: precision, recall, and F1 score to calculate the accuracy of the NLP pipeline when compared with clinician assessment (Table 1) (21). We used GATE's definition of precision as the number of correctly identiﬁed items expressed as a percentage of the number of items identiﬁed, recall as the number of correctly identiﬁed items expressed as a percentage of the total number of correct items, and the F1 score as the harmonic mean of precision and recall (aiming to achieve a balance between precision and recall) (Table 1) (21). Precision is analogous to positive predictive value (PPV) and aims to measure how many of the items identiﬁed by the application are actually correct, irrespective of whether it also failed to retrieve correct items (Table 1) (21). Recall is analogous to sensitivity or the true positive rate and aims to measure how many of the items that should have been identiﬁed actually were identiﬁed, regardless of how many spurious or false positive identiﬁcations were made (Table 1) (21).

**Table 1:** Formulae used to calculate precision, recall, and F1 score.

Measure	Formula
Precision	$=\;\frac{\mathrm{Correct}+\frac{1}{2}\;\mathrm{partial}}{\mathrm{Correct}+\mathrm{spurious}+\;\mathrm{partial}}$
Recall	$=\;\frac{\mathrm{Correct}+\frac{1}{2}\;\mathrm{partial}}{\mathrm{Correct}+\mathrm{missing}+\;\mathrm{partial}}$
F1 score	$=\;\frac{2\times \mathrm{Precision}\times \mathrm{recall}}{\mathrm{Precision}+\mathrm{recall}}$

Partial, two annotations are partially compatible if they overlap and if the features of one (usually the ones from the key) are included in the features of the other (response); Spurious, A response annotation is spurious if either is not coextensive or overlapping, or if one or more features from the key are not included in the response annotation; Missing, a key annotation is missing if either it is not coextensive or overlapping, or if one or more features are not included in the response annotation.

The assessment of partially correct annotations can differ in GATE depending on the intended task of the pipeline (Table 1) (21). “Strict,” “average,” and “lenient” are graded approaches to deriving an F1 score depending on how well the clinician annotation matches or spans that of the data extracted by the algorithm. A “strict” F1 score considers only perfectly matching annotations to be correct, a “lenient” F1 score considers partially matching annotations as correct, whilst an “average” F1 score derives a value from the average of the strict and lenient F1 scores. We did not consider the span of the annotation as an important goal in the generation of our pipeline and so we used a lenient scoring approach to calculating the F-measure.

Each clinician's set of annotations was compared with one another using the Python programming language to calculate the (1) difference in the F1 score and (2) inter-annotator agreement (IAA). The single gold standard validation corpus was initially annotated by two independent and blinded expert clinicians involved in skin cancer care. Following this, a single gold standard validation corpus was then produced following a meeting between the clinician annotators who discussed and resolved disagreements in their annotations to achieve consensus through strict adherence to the annotation guideline and data dictionary. Statistical data analyses were performed using RStudio (R Core Team, R Foundation for Statistical Computing, Vienna, Austria).

## Results

### Training

A total of 2,591 items of information were identified in 41 histopathology reports with an overall precision, recall, and F1 score of 94.9% (95% CI: 88.9–100.0), 95.1% (95% CI: 89.9–100), and 94.8% (95% CI: 89.1–100.0), respectively, when assessed against a single clinician. Table 2a summarises the performance of the NLP pipeline in identifying these items of information.

**Table 2:** Information extracted from (a) training corpus of 41 basal cell carcinoma histopathology reports and (b) validation corpus of 200 BCC histopathology reports.

Entity	Number of annotations created per report by clinician		Number of features in clinician annotation		Mean number of features per clinician annotation		Number of annotations created per report by algorithm		Number of features extracted by algorithm		Mean number of features per algorithm extraction		Match		Missing		Spurious		Partial	
	Training (a)	Validation (b)	Training (a)	Validation (b)	Training (a)	Validation (b)	Training (a)	Validation (b)	Training (a)	Validation (b)	Training (a)	Validation (b)	Training (a)	Validation (b)	Training (a)	Validation (b)	Training (a)	Validation (b)	Training (a)	Validation (b)
Accession number	38	199	38	199	1.0	1.0	38	199	38	199	1.0	1.0	0	0	0	0	0	0	38	199
Excision date	19	5	57	15	3.0	3.0	19	5	57	15	3.0	3.0	0	0	0	0	0	0	19	5
Clinical details	89	345	234	1,054	2.6	3.1	86	330	258	915	3.0	2.8	49	37	7	117	4	101	33	191
Macroscopic details	134	660	503	2,811	3.8	4.3	136	752	546	2,787	4.0	3.7	51	12	24	227	30	309	59	431
Microscopic details	125	682	272	1,530	2.2	2.2	125	624	289	1,322	2.3	2.1	45	11	18	289	24	214	62	399
Microscopic measurements	170	757	538	2,433	3.2	3.2	170	648	626	3,032	3.7	4.7	85	6	10	213	12	94	75	548
Report details	40	157	103	172	2.6	1.1	40	152	103	167	2.6	1.1	27	0	0	43	0	33	13	119
Requestor	20	5	40	10	2.0	2.0	20	5	40	10	2.0	2.0	20	0	0	0	0	0	0	5
Supplementary report	0	10	0	38	0	3.8	0	18	0	44	0	2.4	0	0	0	4	0	12	0	6

Match, fully matching clinician annotation and algorithm extraction.

There was variance in performance amongst the canonical structure of the histopathology report signifying areas where the NLP algorithm was able to undertake NER with less error and reflecting feature complexity. Precision and recall for the categories were: accession number (100.0%, 100.0%), excision date (100.0%, 100.0%), clinical details (96.5%, 93.1%), macroscopic details (81.1%, 83.4%), microscopic details (87.0%, 89.1%), microscopic measurements (94.8%, 95.2%), report details (100.0%, 100.0%), and requestor (100.0%, 100.0%) (Table 3a).

**Table 3:** Performance of the natural language processing pipeline on (a) training and (b) corpus compared to clinician assessment. Values calculated per document across annotation types and averaged across the corpus, displayed with 95% confidence intervals.

Entity	Precision %		Recall %		F1 score %	
	Training (a)	Validation (b)	Training (a)	Validation (b)	Training (a)	Validation (b)
Accession number	100.0	100.0	100.0	100.0	100.0	100.0
Excision date	100.0	100.0	100.0	100.0	100.0	100.0
Clinical details	96.5 (90.3–100)	75.4 (70.2–80.7)	93.1 (86.8–99.4)	73.3 (68.0–78.5)	94.1 (88.2–100)	73.6 (68.4–78.84)
Macroscopic details	81.1 (72.3–89.9)	60.8 (56.3–65.3)	83.4 (75.1–92.3)	66.4 (62.3–70.4)	82.1 (73.4–90.8)	63.0 (58.7–67.3)
Microscopic details	87.0 (79.8–94.3)	74.2 (69.8–78.6)	89.1 (82.7–95.5)	66.9 (62.5–71.3)	87.5 (80.7–94.3)	69.1 (64.8–73.5)
Microscopic measurements	94.8 (91.2–98.5)	83.0 (79.1–86.8)	95.2 (91.2–99.3)	72.8 (68.5–77.1)	94.8 (91.0–98.5)	75.9 (71.8–79.9)
Report details	100.0	83.5 (78.3–88.7)	100.0	81.4 (76.1–86.7)	100.0	81.7 (76.4–87.0)
Requestor	100.0	100.0	100.0	100.0	100.0	100.0

### Validation

A total of 11,224 items of information were identified across 200 histopathology reports in the validation set. Table 2b summarises the performance of the NLP pipeline in identifying these items of information, with Table 3b showing the performance relative to the gold standard. The mean precision, recall, and F1 scores were 86.0% (95% CI: 75.1–96.9), 84.2% (95% CI: 72.8–96.1), and 84.5% (95% CI: 73.0–95.1), respectively. The overall difference between mean clinician annotator F1 scores was 7.9% (Table 4a) in comparison with 15.5% between the NLP pipeline and the gold standard corpus. A confusion matrix was used to identify tokens identified by one annotator but not by the other (Supplementary Table S2). The IAA between each clinician and the gold standard corpus F1 scores was calculated using a specific agreement (Table 4b). A token was characterised by line range, entity, and attributes and assigned to the component of the histology report it belonged to, e.g., clinical details. The overall Cohen's Kappa score on annotated tokens and the F1 score, calculated using pairwise comparisons, were 0.85 and 0.88, respectively.

**Table 4:** Differences in (a) F1 score between annotators on the validation corpus and (b) inter-annotator agreement on the validation corpus.

Entity	(a) Difference in F1 score (%)	(b) Specific agreement
Accession number	0.0	1.00
Excision date	0.0	1.00
Clinical details	1.7	0.95
Macroscopic details	3.9	0.90
Microscopic details	9.5	0.89
Microscopic measurements	2.6	0.97
Report details	0.9	0.99
Requestor	0.0	1.00
Supplementary report	52.4	0.44

**Figure 3:**
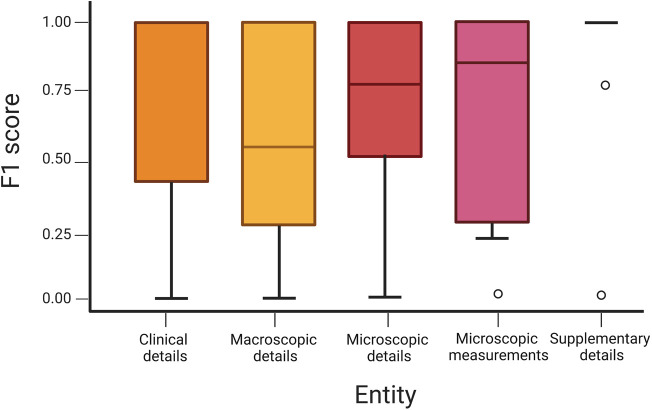
Boxplot of an F1 score across entities demonstrating variance with 95% confidence intervals.

A sub-analysis of the features extracted within the different report entities shows variance in performance by which these data are extracted (Figure 3). For example, if the pipeline is tasked to look at the “tag” feature within the microscopic details entity to calculate BCC incidence and surgical volume, an F1 score of 86.3% (95% CI: 83.7–88.9) is achieved. However, if looking at values for “tumour thickness,” “tumour diameter,” “peripheral clearance,” and “deep clearance” within the entity of microscopic measurements, a lower F1 score of 78.5% (95% CI: 74.6–82.3) is achieved.

Post-hoc analysis revealed that 14 reports (7%) used a form of structured template in the validation corpus, whilst none were used in the training corpus. There were three forms of template used, but no report utilised the “cutaneous basal cell carcinoma removed with therapeutic intent” proforma produced by The Royal College of Pathologists (RCPath) in their 2014 minimum dataset reporting guideline (26). The difference in the performance of our pipeline with and without a template is shown in (Supplementary Table S3). A marginal overall increase in precision (2.5%), recall (1.8%), and F1 score (1.1%) with proforma use is demonstrated.

## Discussion

We have developed and validated a novel NLP pipeline for BCC histopathology reports. This is the first reported use of NLP for NMSC NER, which differs from other more primitive NLP models used for text classification and case identification.

The overall performance of this pipeline was good, and most importantly, it compared well with clinician review (15.5% vs. 7.9%). As to be expected, the pipeline performed better at certain tasks compared with others. For example, when extracting data on accession number, excision date, report details, and requestor, an F1 score of 100% was achieved. Given that these fields consist of fixed format dates that are easier to extract, this is to be expected. More complex entities performed less well, although still with performance close to that expected of an experienced clinician. In terms of disease-specific information, the pipeline performed best in identifying microscopic measurements with an F1 score of 75.9%. These items are frequently mentioned and presented in a relatively standard format, e.g., peripheral clearance 1 mm at 12 O'clock. The entities with the highest drop off in F1 score between validation and training were clinical details, macroscopic details, and microscopic details. In training, the F1 scores were 94.1%, 82.1%, and 87.5%, respectively; however, during validation, they fell to 73.6%, 63.0%, and 69.1%, respectively.

There are a number of explanations for this. A model that performs poorly on internal validation can be described as under-fit or having high bias, commonly caused by insufficient data, or an overly simplistic model (26). It is generally accepted that more data produce better accuracy and higher quality data (closer domain, less noise) (27, 28). Exactly how much data or what quality of data is required to achieve a given performance goal, however, is unclear in the context of NLP as an engineering discipline. Specifically, there are no universally agreed criteria for the required number of documents needed for a gold standard validation corpus (23–25). The training set was generated in 2015, whilst the validation set represented data from 2016 to 2018. Additionally, histopathology reports were written by a group of ten individuals in the training set and 20 in the validation set. This may account for linguistic differences in sentence structure and performance of the JAPE rules that we designed. The population characteristics of the test set, i.e., the reporting style of the pathology reports, should mirror the target population for the algorithm (29). While population characteristics were generally similar, subtle differences are inevitable, and it is difficult to account for this phenomenon whilst reducing selection bias. Despite no proforma being used in the training corpus, a *post-hoc* analysis of the validation data suggests that a structured reporting template such as that developed by the RCPath could improve the performance of our pipeline (26). It is intuitive that designing JAPE rules on a larger training set that includes variants of such a proforma should result in better performance.

Studies assessing error rates in manual clinical data entry demonstrate that rates vary between 0.2% and 26.9% depending on the complexity of interpretation and abstraction of individual data elements (30, 31). Current NMSC-based NLP pipelines report validation on smaller subsets and are centred around text classification and case identification rather than true NER. Lott et al. used NLP on 80,368 histopathology reports to investigate the frequency and percentage of NMSC vs. melanocytic histologic diagnoses and frequency and percentage of melanocytic proliferations classified according to the Melanocytic Pathology Assessment Tool and Hierarchy for Diagnosis (MPATH-Dx) reporting schema (32). A total of 289 original histopathologies were independently reviewed and classified into the MPATH-Dx system by two dermatologists, and any cases with disagreements were reviewed in conjunction to reach consensus. This NLP system yielded a PPV of 82.4%, a sensitivity of 81.7%, and an F1 score of 82%. Eide et al. used NLP to validate NMSC claims-data cases at a large health care system provider and its affiliated health maintenance organisation (33). They set out to define NMSC case volume only and did not report any feature extraction from their NLP pipeline. A comparison of 909 electronic pathology reports to the NLP pipeline showed a sensitivity of 98.3%, a specificity of 99.6%, a negative predictive value of 99.6%, and a PPV of 98.2% for this task. The performance of both these different pipelines reflects how performance can vary depending on the complexity of the intended system task—calculating incidence is a much simpler task compared with pathological diagnosis. We designed our pipeline with the intended purpose of improving the accuracy and to enrich data contained in cancer registries. The remit of the JAPE rules that we created was therefore quite broad, which inherently means that a narrower focus of system task on the same validation set would perform better.

One disadvantage with rule-based NLP is the significant time investment in developing and validating such a pipeline. Building rules, testing, refining, and retesting is a significant undertaking, with the potential for a large amount of further work if crucial aspects are either not included or poorly thought out from the beginning. Therefore, there has been growing interest in the use of ML, instead of rule-based NLP, for information extraction. ML can be broadly categorised into supervised, unsupervised, reinforcement learning, and deep learning. Supervised ML encompasses a model based on a dataset with labelled examples that can be used to solve a classification problem, unsupervised ML is based on a dataset with no labelled examples, and reinforcement learning is a branch of artificial intelligence concerned with the generation of models that aim to maximise the receipt of rewards from an environment by learning to perform specific actions (29). ML can be used in NLP for the purposes of classification (group instances into predefined categories), clustering (group instances into undefined categories), and regression (predict numeric variables) (12). However, other authors have commented how ML algorithms have not been able to demonstrate superior performance in comparison with rule-based techniques as described in this study, are poorly reported, and raise concerns about interpretability and external generalisability (12).

### Strengths and limitations

A significant strength of this study is that a comprehensive NLP algorithm has been developed for NER on 134 features from 139 possible entities in BCC histopathology reports. Additionally, we have gone beyond basic NER since we also capture entity attributes—enriching quality of the data collected. Therefore, a vast amount of data can be extracted from a single histopathology report. The diagnoses within these reports are linked to Unified Medical Language System terminologies mapped to Concept Unique Identifier codes. This platform can be mapped to external coding vocabularies such as the International Classification of Diseases (ICD), Medical Subject Headings (MeSH), and Systematized Nomenclature of Medicine Clinical Terms (SNOMED-CT). As with any early-stage innovation, there are some limitations. Data for training and validation were obtained only from one health board in a single country and therefore the external generalisability of the current pipeline to other hospitals in the UK or differing health services around the world may be limited. We plan to iteratively retest and redevelop our pipeline on larger internal and external datasets to enable future iterations to exhibit improvements in accuracy and allow validation across differing healthcare settings.

## Conclusion

This project is novel within the field of skin oncology in the UK. The information extracted with this system such as tumour subtype, prognostic factors, and margin status are often missing from routinely collected data. We propose that our algorithm has the promise to bridge this data gap enabling further skin cancer research opportunities and in clinical practice to record patient information in a structured manner. Future work will require large-scale external validation of this system with blinded clinician assessment on a gold standard corpus with high inter-annotator agreement.

## Data Availability

The datasets presented in this article are not readily available because all data were anonymised prior to collection. We do not have a data-sharing agreement for the data; however, we are exploring ways of obtaining patient consent and endeavour to produce a minimum dataset for cross-platform testing. Requests to access the datasets should be directed to Stephen.ali@wales.nhs.uk.
