# Supplementary material for: Development and validation of an automated basal cell carcinoma histopathology information extraction system using natural language processing
Source: Front Surg. 2022 Aug 24;9:870494. doi: 10.3389/fsurg.2022.870494 (PMC9683031; doi:10.3389/fsurg.2022.870494)
Supplement: Supplementary file 1 [file Table1.docx]

**Table 1:** Summary of extracted features from BCC histopathology reports.

| **Entity** | **Feature** |
| --- | --- |
| **Accession number** | Value |
| **Excision date** | Day date |
|  | Month date |
|  | Year date |
| **Clinical details** | Tag |
|  | Pre-op diagnosis |
|  | Pre-op diagnosis 2 |
|  | Pre-op diagnosis class |
|  | Pre-op diagnosis class 2 |
|  | Excision type |
|  | Excision type2 |
|  | Excision nature |
|  | Biopsy proven |
|  | Ulcerated |
|  | Body part |
|  | Body part2 |
|  | Multiple scalp |
|  | Lateralization |
|  | Upper or lower |
|  | Anterior or posterior |
|  | Proximal or distal |
|  | Medial or lateral |
|  | Peripheral margin value |
|  | Peripheral margin unit |
|  | Supplemental peripheral margin clock range |
|  | Supplemental peripheral margin clock range 2 |
|  | Deep margin |
|  | Supplemental deep margin clock range |
|  | Supplemental deep margin clock range 2 |
| **Macroscopic details** | Tag |
|  | Pre-op diagnosis |
|  | Pre-op diagnosis 2 |
|  | Ulcerated |
|  | Excision type |
|  | Excision type 2 |
|  | Excision nature |
|  | Body part |
|  | Body part2 |
|  | Multiple scalp |
|  | Lateralization |
|  | Upper or lower |
|  | Anterior or posterior |
|  | Proximal or distal |
|  | Medial or lateral |
|  | 3D specimen size |
|  | 3D specimen size 2 |
|  | 3D specimen size 3 |
|  | 3D specimen size 4 |
|  | Specimen measurement unit |
|  | Max macro tumour diameter |
|  | Max macro measurement unit |
| **Microscopic details** | Tag |
|  | Excision type |
|  | Excision type 2 |
|  | Cancer type |
|  | Cancer type2 |
|  | BCC class |
|  | BCC class 2 |
|  | BCC class 3 |
|  | BCC class 4 |
|  | Differentiation |
|  | Differentiation 2 |
|  | Ulcerated |
|  | Lymphovascular invasion |
|  | Perineural invasion |
|  | Excision completed |
|  | Re-excision outcome |
|  | Recurrent outcome |
|  | Level of invasion |
|  | Clark level |
|  | Stage |
|  | Peripheral clear |
|  | Peripheral clear but close |
|  | Peripheral close |
|  | Supplemental peripheral |
|  | Deep clear |
|  | Deep clear but close |
|  | Deep close |
|  | Supplemental deep |
|  | Frozen section outcome |
| **Microscopic measurements** | Tag |
|  | Measurement type |
|  | Measurement value |
|  | Measurement unit |
|  | Peripheral clock position |
|  | Peripheral clock position 2 |
|  | Peripheral clock position 3 |
|  | Peripheral clock position 4 |
|  | Deep clock position |
|  | Deep clock position 2 |
|  | Deep clock position 3 |
|  | Deep clock position 4 |
| **Requestor** | Clinician name |
|  | Speciality of clinician |
| **Report Details** | Day date |
|  | Month date |
|  | Year date |
|  | Pathologist |
| **Supplementary report** | Tag |
|  | Cancer type |
|  | Cancer type2 |
|  | BCC class |
|  | BCC class 2 |
|  | BCC class 3 |
|  | BCC class 4 |
|  | Differentiation |
|  | Differentiation 2 |
|  | Ulcerated |
|  | Lymphovascular invasion |
|  | Perineural invasion |
|  | Excision completed |
|  | Re-excision outcome |
|  | Recurrent outcome |
|  | Level of invasion |
|  | Clark level |
|  | Stage |
|  | Supplemental peripheral margin |
|  | Supplemental deep margin |
|  | Peripheral clear |
|  | Peripheral clear but close |
|  | Peripheral close |
|  | Deep clear |
|  | Deep clear but close |
|  | Deep close |
|  | Measurement Type |
|  | Measurement Value |
|  | Measurement Unit |
|  | Peripheral clock position |
|  | Peripheral clock position 2 |
|  | Peripheral clock position 3 |
|  | Peripheral clock position 4 |
|  | Deep clock position |
|  | Deep clock position 2 |
|  | Deep clock position 3 |
|  | Deep clock position 4 |
|  | Day date |
|  | Month date |
|  | Year date |
|  | Pathologist |
