# Supplementary material for: Development and validation of an automated basal cell carcinoma histopathology information extraction system using natural language processing
Source: Front Surg. 2022 Aug 24;9:870494. doi: 10.3389/fsurg.2022.870494 (PMC9683031; doi:10.3389/fsurg.2022.870494)
Supplement: Supplementary file 2 [file Table2.docx]

**Table 2:** Confusion matrix from validation corpus data with columns representing annotations by the first clinician and the rows representing annotations by the second clinician**.** Not identified label is used to label a token identified by one annotator but not by the other.

| **Entity** | **Accession number** | **Excision date** | **Clinical details** | **Macroscopic details** | **Microscopic details** | **Microscopic measurements** | **Report details** | **Requestor** | **Supplementary report** | **Not identified** |
| --- | --- | --- | --- | --- | --- | --- | --- | --- | --- | --- |
| **Accession number** | 199 | 0 | 0 | 0 | 0 | 0 | 0 | 0 | 0 | 0 |
| **Excision date** | 0 | 4 | 0 | 0 | 0 | 0 | 0 | 0 | 0 | 0 |
| **Clinical details** | 0 | 0 | 302 | 0 | 0 | 0 | 0 | 0 | 0 | 23 |
| **Macroscopic details** | 0 | 0 | 0 | 602 | 0 | 0 | 0 | 0 | 0 | 80 |
| **Microscopic details** | 0 | 0 | 0 | 0 | 589 | 0 | 0 | 0 | 0 | 58 |
| **Microscopic measurements** | 0 | 0 | 0 | 0 | 0 | 734 | 0 | 0 | 0 | 25 |
| **Report details** | 0 | 0 | 0 | 0 | 0 | 0 | 156 | 0 | 0 | 2 |
| **Requestor** | 0 | 0 | 0 | 0 | 0 | 0 | 0 | 5 | 0 | 0 |
| **Supplementary report** | 0 | 0 | 0 | 0 | 0 | 0 | 0 | 0 | 2 | 2 |
| **Not identified** | 0 | 0 | 10 | 61 | 88 | 18 | 2 | 0 | 3 | 0 |
