# Supplementary material for: Development and validation of an automated basal cell carcinoma histopathology information extraction system using natural language processing
Source: Front Surg. 2022 Aug 24;9:870494. doi: 10.3389/fsurg.2022.870494 (PMC9683031; doi:10.3389/fsurg.2022.870494)
Supplement: Supplementary file 3 [file Table3.docx]

**Table 3:** Performance (a) with and (b) without the use of a report template during validation.

| **Entity** | **Precision %** | | **Recall %** | | **F1 score (%)** | |
| --- | --- | --- | --- | --- | --- | --- |
|  | **With template (a)** | **Without template (b)** | **With template (a)** | **Without template (b)** | **With template (a)** | **Without template (b)** |
| Accession number | 100.0 | 100.0 | 100.0 | 100.0 | 100.0 | 100.0 |
| Excision date | - | 99.7 (99.2-100.0) | - | 100.0 | - | 99.8 (99.5-100.0) |
| Clinical details | 98.2 (94.4-100.0) | 81.8 (77.2-86.4) | 98.2 (94.4-100.0) | 83.6 (79.2- 88.1) | 98.2 (94.4 -100.0) | 81.6 (77.1-86.1) |
| Macroscopic details | 91.2 (82.0-100.0) | 85.8 (82.2-89.4) | 77.9 (66.7-89.1) | 76.4 (72.9-79.9) | 83.4 (73.8-93.0) | 79.8 (76.5-83.2) |
| Microscopic details | 64.7 (49.7-79.7) | 74.8 (70.8.8-78.8) | 72.0 (58.2-85.9) | 82.1 (78.3-85.8) | 66.6 (51.7-81.5) | 76.7 (73.0-80.5) |
| Microscopic measurements | 86.9 (77.6-96.2) | 71.5 (66.7-76.2) | 91.1 (83.3-98.9) | 82.4 (78.1-86.7) | 87.7 (80.6-94.7) | 74.9 (70.4-79.4) |
| Report details | 85.8 (64.7-100.0) | 95.3 (92.5-98.1) | 100.0 | 96.8 (94.2-99.3) | 85.8 (64.7-100.0) | 95.7 (93.0-98.4) |
| Requestor | - | 99.7 (99.2-100.0) | - | 100.0 | - | 99.8 (99.5-100.0) |
| Supplementary report | 100.0 | 100.0 | 92.9 (77.4-100) | 98.2 (96.4-100) | 92.9 (77.4-100) | 98.2 (96.4-100) |
