# Supplementary material for: Development and validation of an automated basal cell carcinoma histopathology information extraction system using natural language processing
Source: Front Surg. 2022 Aug 24;9:870494. doi: 10.3389/fsurg.2022.870494 (PMC9683031; doi:10.3389/fsurg.2022.870494)
Supplement: Supplementary file 4 [file Datasheet1.docx]

**Markup Annotation Protocol v 3.0 June 2021**

Table of Contents

[Introduction 2](#_Toc74568848)

[Sentence Structure 2](#_Toc74568849)

[Clinician Name 2](#_Toc74568850)

[Accession Number 2](#_Toc74568851)

[Clinical Details 3](#_Toc74568852)

[Macro Details 6](#_Toc74568853)

[Micro Details 7](#_Toc74568854)

[Macro Measurements 10](#_Toc74568855)

[Excision Date 11](#_Toc74568856)

[Report Details 12](#_Toc74568857)

[Supplementary Report 12](#_Toc74568858)

[Appendices 16](#_Toc74568859)

[Appendix A: Triggers gazetteer list 16](#_Toc74568860)

[Appendix B: Blocks gazetteer list 18](#_Toc74568861)

[Appendix C: Document details gazetteer list 18](#_Toc74568862)

[Appendix D: Paragraph titles gazetteer list 19](#_Toc74568863)

[Appendix E: Case sensitive gazetteer list 19](#_Toc74568864)

# **Introduction**

Here is the configuration file hosted by https://www.getmarkup.com. All the possible options from the dropdown menus are here. These instructions assume some familiarity with Markup and are aimed to aid the annotation of basal cell carcinoma (BCC) histopathology reports as part of establishing an example annotation set (gold standard) for information extraction from BCC histopathology reports using EXTRACT-BCC. The appendix and lists are linked to help in looking up terms and concept features. This is a list of the entities we are trying to extract data from.

## **Sentence Structure**

Punctuation plays an important part as to whether a sentence should be annotated piecemeal as two separate annotations or en bloc as a whole chunk.

- When quotation marks, hyphens, full stops, semi colons colon and colons are encountered a sentence should be annotated piecemeal as two separate annotations.
- Commas and question marks on the other hand do not count as a sentence break.
- If micro- or macro-measurements are interposed between one another then they should be annotated separate annotations.

## **General Comments**

- The histopathology report is broken down into nine subcategories:
  - Clinician name
  - Accession number
  - Clinical details
  - Macro details
  - Micro details
  - Micro measurements
  - Excision date
  - Report details
  - Supplementary report
- Not all of these subcategories will be commented on in every report.
- With the exception of micro details and micro measurements you can only use the entities and events from the specific colour coded subcategories of the histopathology report to annotate text belonging to that subcategories of the report e.g. do not use laterality from clinical details to annotate laterality reported in macro details.
- Conclusions and summaries of reports are considered supplemental and are therefore annotated as per the supplementary report subcategory.

## **Data Dictionary**

A list of definitions that power the GATE pipeline have been created. If you come across a term and feel there could be ambiguity in its definition please consult this list of terms which will guide your decision in how to annotate.

# **Clinician Name**

| **Entities** | **Events** | **Attributes** |
| --- | --- | --- |
| CliniciansName Arg:Requestor | Value:MarkCooper\|MaxMurison\|DeanBoyce\|PeterDrew\|IanJosty\|LeongHiew\|NicholasWJones\|DaiNguyen\|SarahHGorse\|RichardKaroo\|AmarGhattaura\|MohammedHBasheer\|IainWhitaker\|HywelDafydd\|JeremyYarrow\|ErnestAzzopardi\|TomasTickunas\|TomasONeill\|SophiePJones\|LozHarry\|AhmedEmam\|NickMarsden\|JamesWSmith\|MuhammadJavaid\|JonathanCubitt\|LydiaTang\|ThomasBragg\|RobDuncan\|FredSchreuder\|TomPotokar\|BillDixon\|HamishLaing\|SharonBlackford\|AvadMughal\|SairanWhitaker | Do not need to annotate “Requestor” or the title of the clinician prefixes |
| Speciality Arg:Requestor | Value:Plastics\|Dermatology\|OMFS\|ENT\|GeneralSurgery | This annotation is applied at the same time as the CliniciansName |
| **Accession Number** | | |
| **Entities** | **Events** | **Attributes** |
| AccessionNumber |  | Do not need to annotate the “AccessionNumber” prefix |
| **Clinical Details** | | |
| **Entities** | **Events** | **Attributes** |
| Tag Arg:ClinicalDetails | Value:TypeValue | This function is for reports with multiple samples and to link details from the top of the report to information later on. You will see the tag function in ClinicalDetails, MicroDetails and MicroMeasurements linking entities for each lesion together. Value to be used would be Letter (keep capitalisation) or a Number). If lesions are tagged in a way other than A,B,C... or 1,2,3 but they have been by size (small/large) or proximity (distal/proximal) use the equivalent throughout. If interchangeable terms have been used for each lesion throughout the report use the tag function to tag larger as ‘Tag = Big’ and the other as ‘Tag=Small’, then do the same with superior and inferior. This will keep the features unique for both, and link them to other features in the document. If tag labels are incorrectly labelled in the report then always use labels provided in report rather than impute the correct values |
| Pre-opDiagnosis Arg: ClinicalDetails | Value:BasalCellCarcinoma\|OtherCancerous\|OtherInSitu\|OtherIntermediate\|OtherBenign\|Non-specific |  |
| Pre-opDiagnosis2 Arg: ClinicalDetails | Value:BasalCellCarcinoma\|OtherCancerous\|OtherInSitu\|OtherIntermediate\|OtherBenign\|Non-specific |  |
| Pre-opDiagnosisClass Arg:ClinicalDetails | Value: Nodular\|Superficial\|Infiltrative\|Morphoeic\|Micronodular\|Multinodular\|Pigmented\|Cystic\|Nodulocystic\|Mixed\|Fibroepithelial\|Basosquamous\|Other | These classes relate to BCC only. Therefore, nodule in the following sentence example would not be annotated e.g. “nodular pearly lesion left temple/eyebrow” |
| Pre-opDiagnosisClass2 Arg:ClinicalDetails | Value: Nodular\|Superficial\|Infiltrative\|Morphoeic\|Micronodular\|Multinodular\|Pigmented\|Cystic\|Nodulocystic\|Mixed\|Fibroepithelial\|Basosquamous\|Other |  |
| ExcisionType Arg:ClinicalDetails | Value:ExcisionBiopsy\|WideLocalExcision\|Re-excision\|Mohs\|WedgeExcision\|PunchBiopsy\|IncisionBiopsy\|ShaveBiopsy\|Curettage\|SupplementalDeepMarginSpecimen\|SupplementalPeripheralMarginSpecimen | Re-excision = a further operation on a lesion due to it being incompletely excised/very close margins from the first formal excision |
| ExcisionType2 Arg:ClinicalDetails | Value:ExcisionBiopsy\|WideLocalExcision\|Re-excision\|Mohs\|WedgeExcision\|PunchBiopsy\|IncisionBiopsy\|ShaveBiopsy\|Curettage\|SupplementalDeepMarginSpecimen\|SupplementalPeripheralMarginSpecimen |  |
| ExcisionNature Arg: ClinicalDetails | Value:Primary\|Recurrent\|PreviouslyIncomplete | Primary = the first surgical excision of the lesion (if there had previously been a punch/incision biopsy of the lesion but no formal excision with margins then this is still a 'primary' excision). Note that recurrent ≠ re-excision (see definition in ExcisionType). See Data Dictionary, ExcisionNature.lst for triggers terms of primary, recurrent and previously incomplete. |
| BiopsyProven Arg:ClinicalDetails | Value:Yes\|No |  |
| Ulcerated Arg:ClinicalDetails | Value:Yes\|No |  |
| BodyPart Arg:ClinicalDetails | Value:Face\|Ear\|Pre-auricular\|Post-auricular\|Nose\|Scalp\|Temple\|Cheek\|Eyelid\|MedialCanthus\|LateralCanthus\|Forehead\|Eyebrow\|Chin\|Lip\|Neck\|Chest\|Abdomen\|Back\|Arm\|Leg\|Hand\|Foot\|NailComplex\|Thigh\|Genitourinary |  |
| BodyPart2 Arg:ClinicalDetails | Value:Face\|Ear\|Pre-auricular\|Post-auricular\|Nose\|Scalp\|Temple\|Cheek\|Eyelid\|MedialCanthus\|LateralCanthus\|Forehead\|Eyebrow\|Chin\|Lip\|Neck\|Chest\|Abdomen\|Back\|Arm\|Leg\|Hand\|Foot\|NailComplex\|Thigh\|Genitourinary |  |
| MultipleScalp Arg: ClinicalDetails | Value:Frontal\|Vertex\|Temporal\|Parietal\|Occipital\|Nuchal |  |
| Lateralization Arg:ClinicalDetails | Value:Left\|Right\|Midline |  |
| UpperOrLower Arg:ClinicalDetails | Value:Upper\|Lower |  |
| AnteriorOrPosterior Arg:ClinicalDetails | Value:Anterior\|Posterior |  |
| ProximalOrDistal Arg:ClinicalDetails | Value:Proximal\|Distal |  |
| MedialOrLateral Arg:ClinicalDetails | Value:Medial\|Lateral |  |
| PeripheralMarginValue Arg:ClinicalDetails | Value:TypeNumberOnly |  |
| PeripheralMarginUnit Arg:ClinicalDetails | Value:mm |  |
| SupplementalPeripheralMarginClockRange Arg:ClinicalDetails | Value:TypeNumberOnly | If a second sample of the peripheral margin has been taken |
| SupplementalPeripheralMarginClockRange2 Arg:ClinicalDetails | Value:TypeNumberOnly |  |
| DeepMargin Arg:ClinicalDetails | Value: Dermis\|Fat\|Fascia\|SuperficialFascia\|DeepFascia\|Galea\|Periosteum\|SMAS\|Muscle\|Bone\|Cartilage\|Perichondrium\|Other | This is an exclusive function e.g. scalp lesion excised down to periosteum then periosteum would be selected as the deep margin |
| SupplementalDeepMarginClockRange Arg:ClinicalDetails | Value:TypeNumberOnly | If a second sample of the deep margin has been taken |
| SupplementalDeepMarginClockRange2 Arg:ClinicalDetails | Value:TypeNumberOnly |  |
| **Macro Details** | | |
| **Entities** | **Events** | **Attributes** |
| Tag Arg:MacroDetails | Value:TypeValue | Remember to tag each of these features if multiple lesions |
| Pre-opDiagnosis Arg: MacroDetails | Value:BasalCellCarcinoma\|OtherCancerous\|OtherInSitu\|OtherIntermediate\|OtherBenign\|Non-specific |  |
| Pre-opDiagnosis2 Arg: MacroDetails | Value:BasalCellCarcinoma\|OtherCancerous\|OtherInSitu\|OtherIntermediate\|OtherBenign\|Non-specific |  |
| Ulcerated Arg:MacroDetails | Value:Yes\|No |  |
| ExcisionType Arg:MacroDetails | Value:ExcisionBiopsy\|WideLocalExcision\|Re-excision\|Mohs\|WedgeExcision\|PunchBiopsy\|IncisionBiopsy\|ShaveBiopsy\|Curettage\|SupplementalDeepMarginSpecimen\|SupplementalPeripheralMarginSpecimen |  |
| ExcisionType2 Arg:MacroDetails | Value:ExcisionBiopsy\|WideLocalExcision\|Re-excision\|Mohs\|WedgeExcision\|PunchBiopsy\|IncisionBiopsy\|ShaveBiopsy\|Curettage\|SupplementalDeepMarginSpecimen\|SupplementalPeripheralMarginSpecimen |  |
| ExcisionNature Arg: MacroDetails | Value:Primary\|Recurrent\|PreviouslyIncomplete |  |
| BodyPart Arg:MacroDetails | Value:Face\|Ear\|Pre-auricular\|Post-auricular\|Nose\|Scalp\|Temple\|Cheek\|Eyelid\|MedialCanthus\|LateralCanthus\|Forehead\|Eyebrow\|Chin\|Lip\|Neck\|Chest\|Abdomen\|Back\|Arm\|Leg\|Hand\|Foot\|NailComplex\|Thigh\|Genitourinary |  |
| BodyPart2 Arg:MacroDetails | Value:Face\|Ear\|Pre-auricular\|Post-auricular\|Nose\|Scalp\|Temple\|Cheek\|Eyelid\|MedialCanthus\|LateralCanthus\|Forehead\|Eyebrow\|Chin\|Lip\|Neck\|Chest\|Abdomen\|Back\|Arm\|Leg\|Hand\|Foot\|NailComplex\|Thigh\|Genitourinary |  |
| MultipleScalp Arg: MacroDetails | Value:Frontal\|Vertex\|Temporal\|Parietal\|Occipital\|Nuchal\|Fronto-temporal\|Fronto-parietal\|PanScalp |  |
| Lateralization Arg:MacroDetails | Value:Left\|Right\|Midline |  |
| UpperOrLower Arg:MacroDetails | Value:Upper\|Lower |  |
| AnteriorOrPosterior Arg:MacroDetails | Value:Anterior\|Posterior |  |
| ProximalOrDistal Arg:MacroDetails | Value:Proximal\|Distal |  |
| MedialOrLateral Arg:MacroDetails | Value:Medial\|Lateral |  |
| 3DSpecimenSize Arg: MacroDetails | Value:TypeNumberOnly | Specimen size not tumour size. If two specimen hourglass shaped or irregular in any other way, record the largest of each dimension |
| 3DSpecimenSize2 Arg: MacroDetails | Value:TypeNumberOnly |  |
| 3DSpecimenSize3 Arg: MacroDetails | Value:TypeNumberOnly |  |
| 3DSpecimenSize4 Arg: MacroDetails | Value:TypeNumberOnly |  |
| SpecimenMeasurementUnit Arg:MacroDetails | Value:mm |  |
| MaxMacroTumourDiameter Arg:MacroDetails | Value:TypeNumberOnly | Tumour size not specimen size |
| MaxMacroMeasurementUnit Arg:MacroDetails | Value:mm |  |
| **Micro Details** | | |
| **Entities** | **Events** | **Attributes** |
| Tag Arg:MicroDetails | Value:TypeValue | Remember to tag each of these features if multiple lesions |
| ExcisionType Arg:MicroDetails | Value:ExcisionBiopsy\|WideLocalExcision\|Re-excision\|Mohs\|WedgeExcision\|PunchBiopsy\|IncisionBiopsy\|ShaveBiopsy\|Curettage\|SupplementalDeepMarginSpecimen\|SupplementalPeripheralMarginSpecimen |  |
| ExcisionType2 Arg:MicroDetails | Value:ExcisionBiopsy\|WideLocalExcision\|Re-excision\|Mohs\|WedgeExcision\|PunchBiopsy\|IncisionBiopsy\|ShaveBiopsy\|Curettage\|SupplementalDeepMarginSpecimen\|SupplementalPeripheralMarginSpecimen |  |
| CancerType Arg:MicroDetails | Value: BasalCellCarcinoma\|OtherCancerous\|OtherInSitu\|OtherIntermediate\|OtherBenign\|Non-specific |  |
| CancerType2 Arg:MicroDetails | Value: BasalCellCarcinoma\|OtherCancerous\|OtherInSitu\|OtherIntermediate\|OtherBenign\|Non-specific |  |
| BCCClass Arg:MicroDetails | Value: Nodular\|Superficial\|Infiltrative\|Morphoeic\|Micronodular\|Multinodular\|\|Multifocal\|Pigmented\|Cystic\|Nodulocystic\|Mixed\|Fibroepithelial\|Basosquamous\|Other | BCC subtypes need to be annotated in order of appearance. Use “mixed” as a discrete entity in addition to the other entities in the report |
| BCCClass2 Arg:MicroDetails | Value: Nodular\|Superficial\|Infiltrative\|Morphoeic\|Micronodular\|Multinodular\|\|Multifocal\|Pigmented\|Cystic\|Nodulocystic\|Mixed\|Fibroepithelial\|Basosquamous\|Other |  |
| BCCClass3 Arg:MicroDetails | Value: Nodular\|Superficial\|Infiltrative\|Morphoeic\|Micronodular\|Multinodular\|\|Multifocal\|Pigmented\|Cystic\|Nodulocystic\|Mixed\|Fibroepithelial\|Basosquamous\|Other |  |
| BCCClass4 Arg:MicroDetails | Value: Nodular\|Superficial\|Infiltrative\|Morphoeic\|Micronodular\|Multinodular\|\|Multifocal\|Pigmented\|Cystic\|Nodulocystic\|Mixed\|Fibroepithelial\|Basosquamous\|Other |  |
| Differentiation Arg:MicroDetails | Value: Adamantinoid\|GranularCell\|ClearCell\|GiantCell\|SignetCell\|Adenoid\|Keratotic\|Pleiomorphic\|Ductal\|Glandular\|Adnexal\|Eccrine\|Apocrine\|Sebaceous\|Infundibulocystic\|Follicular\|Matricial\|Myoepithelial\|Neuroendocrine\|Squamous | If the pathologist has reported a differential diagnosis |
| Differentiation2 Arg:MicroDetails | Value: Adamantinoid\|GranularCell\|ClearCell\|GiantCell\|SignetCell\|Adenoid\|Keratotic\|Pleiomorphic\|Ductal\|Glandular\|Adnexal\|Eccrine\|Apocrine\|Sebaceous\|Infundibulocystic\|Follicular\|Matricial\|Myoepithelial\|Neuroendocrine\|Squamous |  |
| Ulcerated Arg:MicroDetails | Value:Yes\|No |  |
| LymphovascularInvasion Arg:MicroDetails | Value:Yes\|No |  |
| PerineuralInvasion Arg:MicroDetails | Value:Yes\|No |  |
| ExcisionCompleted Arg:MicroDetails | Value:Yes\|No |  |
| Re-excisionOutcome Arg:MicroDetails | Value: ResidualDiseasePresent\|NoResidualDiseasePresent |  |
| RecurrentOutcome Arg:MicroDetails | Value: RecurrentDiseasePresent\|NoRecurrentDiseasePresent |  |
| LevelOfInvasion Arg:MicroDetails | Value: Dermis\|Fat\|Fascia\|SuperficialFascia\|DeepFascia\|Galea\|Periosteum\|SMAS\|Muscle\|Bone\|Cartilage\|Perichondrium\|Other |  |
| ClarkLevel Arg:MicroDetails | Value: I\|II\|III\|IV\|V |  |
| Stage Arg:MicroDetails | Value: pT1\|pT1a\|pT1b\|pT2\|pT2a\|pT2b\|pT3\|pT3a\|pT3b\|pT4\|pT4a\|pT4b |  |
| PeripheralClear Arg:MicroDetails | Value:Yes\|No | A positive peripheral margin should be referenced with a numeric value but if “clear” or “>1mm” has been used as a descriptive term select and apply this annotation |
| PeripheralClearButClose Arg:MicroDetails | Value:Yes\|No | If "clear but close" has been used as a descriptive term then select and apply this annotation |
| PeripheralClose Arg:MicroDetails | Value:Yes\|No | If “close”, “<1mm” or “less than 1mm” have been has been used as a descriptive term then select and apply this annotation |
| SupplementalPeripheral Arg:MicroDetails | Value:Positive\|Negative |  |
| DeepClear Arg:MicroDetails | Value:Yes\|No | A positive deep margin should be referenced with a numeric value but if “clear” or “>1mm” has been used as a descriptive term select and apply this annotation |
| DeepClearButClose Arg:MicroDetails | Value:Yes\|No | If "clear but close" has been used as a descriptive term then select and apply this annotation |
| DeepClose Arg:MicroDetails | Value:Yes\|No | If “close”, “<1mm” or “less than 1mm” have been used as a descriptive term then select and apply this annotation |
| DeepClearButClose Arg:MicroDetails | Value:Yes\|No |  |
| SupplementalDeep Arg:MicroDetails | Value:Positive\|Negative |  |
| FrozenSectionOutcome Arg:MicroDetails | Value:BasalCellCarcinoma\|OtherCancerous\|OtherInSitu\|OtherIntermediate\|OtherBenign\|Non-specific |  |
| **Micro Measurements** | | |
| **Entities** | **Events** | **Attributes** |
| Tag Arg:MicroMeasurements | Value:TypeValue | Remember to tag each of these features if multiple lesions |
| MeasurementType Arg:MicroMeasurements | Value:TumourThickness\|DeepClearance\|PeripheralClearance\|Diameter | Tumour depth equates to tumour depth |
| MeasurementValue Arg:MicroMeasurements | Value:TypeNumberOnly | If the report says “involved” or “present” for peripheral or deep margin impute 0mm instead see PeripheralClear or DeepClear Yes\|No |
| MeasurementUnit Arg:MicroMeasurements | Value:mm |  |
| PeripheralClockPosition Arg:MicroMeasurements | Value:TypeNumberOnly | If there is a scenario with multiple peripheral or deep margins (involved or uninvolved) cited, sequentially annotate in order of appearance i.e. in essence record all margins regardless of clearance status |
| PeripheralClockPosition2 Arg:MicroMeasurements | Value:TypeNumberOnly |  |
| PeripheralClockPosition3 Arg:MicroMeasurements | Value:TypeNumberOnly |  |
| PeripheralClockPosition4 Arg:MicroMeasurements | Value:TypeNumberOnly |  |
| DeepClockPosition Arg:MicroMeasurements | Value:TypeNumberOnly | If there is a scenario of equivalent positive deep margins. Sequentially annotate in order of appearance in addition to “DeepClearance” in “MeasurementType Arg:MicroMeasurements". If multiple margins are reported please record all of these values |
| DeepClockPosition2 Arg:MicroMeasurements | Value:TypeNumberOnly |  |
| DeepClockPosition3 Arg:MicroMeasurements | Value:TypeNumberOnly |  |
| DeepClockPosition4 Arg:MicroMeasurements | Value:TypeNumberOnly |  |
| **Excision Date** | | |
| **Entities** | **Events** | **Attributes** |
| DayDate Arg:ExcisionDate | Value:0\|1\|2\|3\|4\|5\|6\|7\|8\|9\|10\|11\|12\|13\|14\|15\|16\|17\|18\|19\|20\|21\|22\|23\|24\|25\|26\|27\|28\|29\|30\|31 | Annotate the “Date/Time Collected” with the earliest timestamp. Ignore Date/Time Received”  Ensure annotated as 01/02/2020 **and not** 1/2/2020 |
| MonthDate Arg:ExcisionDate | Value:0\|1\|2\|3\|4\|5\|6\|7\|8\|9\|10\|11\|12 |  |
| YearDate Arg:ExcisionDate | Value:0\|2020\|2019\|2018\|2017\|2016\|2015\|2014\|2013\|2012\|2011\|2010\|2009\|2008\|2007\|2006\|2005\|2004\|2003\|2002\|2001\|2000 |  |
| **Report Details** | | |
| **Entities** | **Events** | **Attributes** |
| DayDate Arg:ReportDetails | Value:0\|1\|2\|3\|4\|5\|6\|7\|8\|9\|10\|11\|12\|13\|14\|15\|16\|17\|18\|19\|20\|21\|22\|23\|24\|25\|26\|27\|28\|29\|30\|31 | When the following text is encountered please annotate the underlined text below only e.g. miss the first instance and only annotate the last.  “Reported and authorised by Dr S. Roberts  Surgical Histology: Authorised by PATH: Dr Shaun Roberts on 03/09/15 at 22:23’  Ensure annotated as 01/02/2020 **and not** 1/2/2020 |
| MonthDate Arg:ReportDetails | Value:0\|1\|2\|3\|4\|5\|6\|7\|8\|9\|10\|11\|12 |  |
| YearDate Arg:ReportDetails | Value:0\|2020\|2019\|2018\|2017\|2016\|2015\|2014\|2013\|2012\|2011\|2010\|2009\|2008\|2007\|2006\|2005\|2004\|2003\|2002\|2001\|2000 |  |
| Pathologist Arg:ReportDetails | Value:ShaunRoberts\|AllanDawson\|MaurizioBrotto\|PaulGriffiths\|NamorWilliams\|SusannahHowarth\|PeterDavis\|NadineBurke\|NasimaTofazzal\|DannyParker\|AlisonFinall\|GarethLeopold\|LeonidSemkin\|MargaretCotter\|OlexandraKozyar\|PeterDavis\|RhiannonTrefor\|SelwynNg\|TawfikElazzabi\|VarshaShah\|DavidWilliams\|Other |  |
| **Supplementary Report** | | |
| **Entities** | **Events** | **Attributes** |
| Tag Arg:SupplementaryReport | Value:TypeValue | Remember to tag each of these features if multiple lesions |
| SRCancerType Arg:SupplementaryReport | Value: BasalCellCarcinoma\|OtherCancerous\|OtherInSitu\|OtherIntermediate\|OtherBenign\|Non-specific |  |
| SRCancerType2 Arg:SupplementaryReport | Value: BasalCellCarcinoma\|OtherCancerous\|OtherInSitu\|OtherIntermediate\|OtherBenign\|Non-specific |  |
| SRBCCClass Arg:SupplementaryReport | Value: Nodular\|Superficial\|Infiltrative\|Morphoeic\|Micronodular\|Multinodular\|\|Multifocal\|Pigmented\|Cystic\|Nodulocystic\|Mixed\|Fibroepithelial\|Basosquamous\|Polypoid\|Other |  |
| SRBCCClass2 Arg:SupplementaryReport | Value: Nodular\|Superficial\|Infiltrative\|Morphoeic\|Micronodular\|Multinodular\|\|Multifocal\|Pigmented\|Cystic\|Nodulocystic\|Mixed\|Fibroepithelial\|Basosquamous\|Polypoid\|Other |  |
| SRBCCClass3 Arg:SupplementaryReport | Value: Nodular\|Superficial\|Infiltrative\|Morphoeic\|Micronodular\|Multinodular\|\|Multifocal\|Pigmented\|Cystic\|Nodulocystic\|Mixed\|Fibroepithelial\|Basosquamous\|Polypoid\|Other |  |
| SRBCCClass4 Arg:SupplementaryReport | Value: Nodular\|Superficial\|Infiltrative\|Morphoeic\|Micronodular\|Multinodular\|\|Multifocal\|Pigmented\|Cystic\|Nodulocystic\|Mixed\|Fibroepithelial\|Basosquamous\|Polypoid\|Other |  |
| SRDifferentiation Arg:SupplementaryReport | Value: Adamantinoid\|GranularCell\|ClearCell\|GiantCell\|SignetCell\|Adenoid\|Keratotic\|Pleiomorphic\|Ductal\|Glandular\|Adnexal\|Eccrine\|Apocrine\|Sebaceous\|Infundibulocystic\|Follicular\|Matricial\|Myoepithelial\|Neuroendocrine\|Squamous | If the pathologist has reported a differential diagnosis |
| SRDifferentiation2 Arg:SupplementaryReport | Value: Adamantinoid\|GranularCell\|ClearCell\|GiantCell\|SignetCell\|Adenoid\|Keratotic\|Pleiomorphic\|Ductal\|Glandular\|Adnexal\|Eccrine\|Apocrine\|Sebaceous\|Infundibulocystic\|Follicular\|Matricial\|Myoepithelial\|Neuroendocrine\|Squamous |  |
| SRUlcerated Arg:SupplementaryReport | Value:Yes\|No |  |
| SRLymphovascularInvasion Arg:SupplementaryReport | Value:Yes\|No |  |
| SRPerineuralInvasion Arg:SupplementaryReport | Value:Yes\|No |  |
| SRExcisionCompleted Arg:SupplementaryReport | Value:Yes\|No |  |
| SRRe-excisionOutcome Arg:SupplementaryReport | Value: ResidualDiseasePresent\|NoResidualDiseasePresent |  |
| SRRecurrentOutcome Arg:SupplementaryReport | Value: RecurrentDiseasePresent\|NoRecurrentDiseasePresent |  |
| SRLevelOfInvasion Arg:SupplementaryReport | Value: Dermis\|Fat\|Fascia\|SuperficialFascia\|DeepFascia\|Galea\|Periosteum\|SMAS\|Muscle\|Bone\|Cartilage\|Perichondrium\|Other |  |
| SRClarkLevel Arg:SupplementaryReport | Value: I\|II\|III\|IV\|V |  |
| SRStage Arg:SupplementaryReport | Value: pT1\|pT1a\|pT1b\|pT2\|pT2a\|pT2b\|pT3\|pT3a\|pT3b\|pT4\|pT4a\|pT4b |  |
| SRPeripheralClear Arg:SupplementaryReport | Value:Yes\|No |  |
| SRPeripheralClearButClose Arg:SupplementaryReport | Value:Yes\|No |  |
| SRSupplementalPeripheral Arg:SupplementaryReport | Value:Positive\|Negative |  |
| SRDeepClear Arg:SupplementaryReport | Value:Yes\|No |  |
| SRDeepClearButClose Arg:SupplementaryReport | Value:Yes\|No |  |
| SRSupplementalDeep Arg:SupplementaryReport | Value:Positive\|Negative |  |
| SRFrozenSectionOutcome Arg:MicroDetails | Value:BasalCellCarcinoma\|OtherCancerous\|OtherInSitu\|OtherIntermediate\|OtherBenign\|Non-specific |  |
| SRDayDate Arg:SupplementaryReport | Value:0\|1\|2\|3\|4\|5\|6\|7\|8\|9\|10\|11\|12\|13\|14\|15\|16\|17\|18\|19\|20\|21\|22\|23\|24\|25\|26\|27\|28\|29\|30\|31 |  |
| SRMonthDate Arg:SupplementaryReport | Value:0\|1\|2\|3\|4\|5\|6\|7\|8\|9\|10\|11\|12 |  |
| SRYearDate Arg:SupplementaryReport | Value:0\|2020\|2019\|2018\|2017\|2016\|2015\|2014\|2013\|2012\|2011\|2010\|2009\|2008\|2007\|2006\|2005\|2004\|2003\|2002\|2001\|2000 |  |
| SRPathologist Arg:SupplementaryReport | Value:ShaunRoberts\|AllanDawson\|MaurizioBrotto\|PaulGriffiths\|NamorWilliams\|SusannahHowarth\|PeterDavis\|NadineBurke\|NasimaTofazzal\|DannyParker\|AlisonFinall\|GarethLeopold\|LeonidSemkin\|MargaretCotter\|OlexandraKozyar\|PeterDavis\|RhiannonTrefor\|SelwynNg\|TawfikElazzabi\|VarshaShah\|DavidWilliams\|Other |  |

# **Appendices**

## **Appendix A:** Triggers gazetteer list

| **List name** | **Major type** | **Minor type** | **Language** | **Annotation type** |
| --- | --- | --- | --- | --- |
| AnteriorOrPosterior.lst |  |  |  | AnteriorOrPosterior |
| BCCClass.lst |  |  |  | BCCClass |
| BCCClassLinkTerms.lst |  |  |  | BCCClassLinkTerms |
| BCCStage.lst |  |  |  | BCCStage |
| BiopsyProven.lst |  |  |  | BiopsyProven |
| BodyParts.lst |  |  |  | BodyParts |
| ClarkLevel.lst |  |  |  | ClarkLevel |
| ClarkLevelOfInvasionTrigger.lst |  |  |  | LevelOfInvasionTrigger |
| Clear.lst |  |  |  | Clear |
| ClearButClose.lst |  |  |  | ClearButClose |
| ClinicalDetailCleaningTrigger.lst |  |  |  | ClinicalDetailCleaningTrigger |
| ClinicalDetailMargin.lst |  |  |  | ClinicalDetailMargin |
| Close.lst |  |  |  | Close |
| DeepMargin.lst |  |  |  | DeepMargin |
| DeepMarginTrigger.lst |  |  |  | DeepMarginTrigger |
| Diagnosis.lst |  |  |  | Diagnosis |
| Diameter.lst |  |  |  | Diameter |
| Differentiation.lst |  |  |  | Differentiation |
| Dimensions.lst |  |  |  | Dimensions |
| Distance_to.lst |  |  |  | Distance_to |
| doubleTag.lst |  |  |  | doubleTag |
| ExcisionCompleted.lst |  |  |  | ExcisionCompleted |
| ExcisionNature.lst |  |  |  | ExcisionNature |
| ExcisionType.lst |  |  |  | ExcisionType |
| Frozen_Section_Triggers.lst |  |  |  | Frozen_Section_Triggers |
| Frozen_Section.lst |  |  |  | Frozen_Section |
| Lesion.lst |  |  |  | Lesion |
| LevelOfInvasionTrigger.lst |  |  |  | LevelOfInvasionTrigger |
| LVI.lst |  |  |  | LVI |
| LVIorPNI.lst |  |  |  | LVIorPNI |
| MarginWordDistances.lst |  |  |  | MarginWordDistances |
| MedialOrLateral.lst |  |  |  | MedialOrLateral |
| Micro deep margin.lst | DeepClearance |  |  | Micro_deep_margin |
| Micro peripheral margin.lst | PeripheralClearance |  |  | Micro_peripheral_margin |
| Micro_deep_margin_maybe.lst |  |  |  | Micro_deep_margin_maybe |
| Micro_peripheral_margin_maybe.lst |  |  |  | Micro_peripheral_margin_maybe |
| MultipleScalp.lst |  |  |  | MultipleScalp |
| o'clock.lst |  |  |  | Clock |
| PNI.lst |  |  |  | PNI |
| ProximalOrDistal.lst |  |  |  | ProximalOrDistal |
| RecurrentDisease.lst |  |  |  | RecurrentDisease |
| ResidualDisease.lst |  |  |  | ResidualDisease |
| SingleMargin.lst |  |  |  | SingleMargin |
| Specimen.lst |  |  |  | Specimen |
| StageTrigger.lst |  |  |  | StageTrigger |
| SupplementalExcisionTypeOutcome.lst |  |  |  | SupplementalExcisionTypeOutcome |
| Thickness.lst |  |  |  | Micro_thickness |
| Ulcerated.lst |  |  |  | Ulcerated |
| UlceratedTrigger.lst |  |  |  | UlceratedTrigger |
| UpperOrLower.lst |  |  |  | UpperOrLower |

## **Appendix B:** Blocks gazetteer list

| **List name** | **Major type** | **Minor type** | **Language** | **Annotation type** |
| --- | --- | --- | --- | --- |
| AdditionalSentenceBreak.lst |  |  |  | LocalisationSkipTerms |
| FrozenSectionBlocks.lst |  |  |  | AdditionalSentenceBreak |
| LocalisationSkipTerms.lst |  |  |  | FrozenSectionBlocks |

## **Appendix C:** Document details gazetteer list

| **List name** | **Major type** | **Minor type** | **Language** | **Annotation type** |
| --- | --- | --- | --- | --- |
| AccessionNumber.lst |  |  |  | AccessionNumber_trigger |
| Excisiondate_trigger.lst |  |  |  | Excisiondate_trigger |
| Reported_triggers.lst |  |  |  | Reported_triggers |
| Reporter.lst | Person | Reporter |  | VIP |
| Requestor_triggers.lst |  |  |  | Requestor_triggers |
| Requestor.lst | Person | Speciality |  | VIP |
| SupplementaryReport_trigger.lst |  |  |  | SupplementaryReport_trigger |
| Titles.lst |  |  |  | Titles |

## **Appendix D:** Paragraph titles gazetteer list

| **List name** | **Major type** | **Minor type** | **Language** | **Annotation type** |
| --- | --- | --- | --- | --- |
| ParagraphTitles.lst |  |  |  | ParagraphTitles |

## **Appendix E:** Case sensitive gazetteer list

| **List name** | **Major type** | **Minor type** | **Language** | **Annotation type** |
| --- | --- | --- | --- | --- |
| CaseSensitiveDiagnosis.lst |  |  |  | CaseSensitiveDiagnosis |
