# Supplementary material for: Development and validation of an automated basal cell carcinoma histopathology information extraction system using natural language processing
Source: Front Surg. 2022 Aug 24;9:870494. doi: 10.3389/fsurg.2022.870494 (PMC9683031; doi:10.3389/fsurg.2022.870494)
Supplement: Supplementary file 5 [file Datasheet2.docx]

**Markup Data Dictionary**

The definitions here have used the 2020 World Health Organisation Classification of Tumors of Soft Tissue and have attempted to be exhaustive but if you notice any missing terms then please contact the corresponding author.

Table of Contents

[Triggers 2](#_Toc74567224)

[*AnteriorOrPosterior.lst* 2](#_Toc74567225)

[*BCCClass.lst* 2](#_Toc74567226)

[*BCCClassLinkTerms.lst* 4](#_Toc74567227)

[*BCCStage.lst* 4](#_Toc74567228)

[*BiopsyProven.lst* 4](#_Toc74567229)

[*BodyParts.lst* 5](#_Toc74567230)

[*ClarkLevel.lst* 22](#_Toc74567231)

[*ClarkLevelOfInvasionTrigger.lst* 23](#_Toc74567232)

[*Clear.lst* 23](#_Toc74567233)

[*ClearButClose.lst* 23](#_Toc74567234)

[*ClinicalDetailCleaningTrigger.lst* 23](#_Toc74567235)

[*ClinicalDetailMargin.lst* 23](#_Toc74567236)

[*Close.lst* 23](#_Toc74567237)

[*DeepMargin.lst* 24](#_Toc74567238)

[*DeepMarginTrigger.lst* 31](#_Toc74567239)

[*Diagnosis.lst* 31](#_Toc74567240)

[*Diameter.lst* 50](#_Toc74567241)

[*Differentiation.lst* 50](#_Toc74567242)

[*Dimensions.lst* 51](#_Toc74567243)

[*Distance_to.lst* 51](#_Toc74567244)

[*doubleTag.lst* 52](#_Toc74567245)

[*ExcisionCompleted.lst* 52](#_Toc74567246)

[*ExcisionNature.lst* 53](#_Toc74567247)

[*ExcisionType.lst* 54](#_Toc74567248)

[*Frozen_Section_Triggers.lst* 57](#_Toc74567249)

[*Frozen_Section.lst* 57](#_Toc74567250)

[*Lesion.lst* 57](#_Toc74567251)

[*LevelOfInvasionTrigger.lst* 58](#_Toc74567252)

[*LVI.lst* 59](#_Toc74567253)

[*LVIorPNI.lst* 60](#_Toc74567254)

[*MarginWordDistances.lst* 60](#_Toc74567255)

[*MedialOrLateral.lst* 60](#_Toc74567256)

[*Micro deep margin.lst* 60](#_Toc74567257)

[*Micro peripheral margin.lst* 61](#_Toc74567258)

[*Micro_deep_margin_maybe.lst* 62](#_Toc74567259)

[*Micro_peripheral_margin_maybe.lst* 62](#_Toc74567260)

[*MultipleScalp.lst* 62](#_Toc74567261)

[*o'clock.lst* 63](#_Toc74567262)

[*PNI.lst* 63](#_Toc74567263)

[*ProximalOrDistal.lst* 64](#_Toc74567264)

[*RecurrentDisease.lst* 64](#_Toc74567265)

[*ResidualDisease.lst* 65](#_Toc74567266)

[*SingleMargin.lst* 66](#_Toc74567267)

[*Specimen.lst* 66](#_Toc74567268)

[*StageTrigger.lst* 66](#_Toc74567269)

[*SupplementalExcisionTypeOutcome.lst* 67](#_Toc74567270)

[*Thickness.lst* 67](#_Toc74567271)

[*Ulcerated.lst* 68](#_Toc74567272)

[*UlceratedTrigger.lst* 69](#_Toc74567273)

[*UpperOrLower.lst* 69](#_Toc74567274)

[Blocks 69](#_Toc74567275)

[*AdditionalSentenceBreak.lst* 69](#_Toc74567276)

[*FrozenSectionBlocks.lst* 69](#_Toc74567277)

[*LocalisationSkipTerms.lst* 70](#_Toc74567278)

[Document details 70](#_Toc74567279)

[*AccessionNumber.lst* 70](#_Toc74567280)

[*Excisiondate_trigger.lst* 70](#_Toc74567281)

[*Reported_triggers.lst* 70](#_Toc74567282)

[*Reporter.lst* 70](#_Toc74567283)

[*Requestor_triggers.lst* 72](#_Toc74567284)

[*Requestor.lst* 72](#_Toc74567285)

[*SupplementaryReport_trigger.lst* 76](#_Toc74567286)

[*Titles.lst* 76](#_Toc74567287)

[Paragraph titles 76](#_Toc74567288)

[*ParagraphTitles.lst* 76](#_Toc74567289)

[Case sensitive 77](#_Toc74567290)

[*CaseSensitiveDiagnosis.lst* 77](#_Toc74567291)

# **Triggers**

## *AnteriorOrPosterior.lst*

| **Value** | **Feature 1** | **Value 1** |
| --- | --- | --- |
| Anterior | AnteriorOrPosterior | Anterior |
| Dorsal | AnteriorOrPosterior | Posterior |
| Posterior | AnteriorOrPosterior | Posterior |
| Ventral | AnteriorOrPosterior | Anterior |

## *BCCClass.lst*

| **Value** | **Feature 1** | **Value 1** |
| --- | --- | --- |
| Basal/squamous | BCCClass | Basosquamous |
| Basal/ squamous | BCCClass | Basosquamous |
| Basal/squamous cell carcinoma | BCCClass | Basosquamous |
| Basalsquamous | BCCClass | Basosquamous |
| Basal-squamous | BCCClass | Basosquamous |
| Basal- squamous | BCCClass | Basosquamous |
| Basalsquamous cell carcinoma | BCCClass | Basosquamous |
| Basal-squamous cell carcinoma | BCCClass | Basosquamous |
| Basal- squamous cell carcinoma | BCCClass | Basosquamous |
| Basilloma | BCCClass | Basosquamous |
| Basill -oma | BCCClass | Basosquamous |
| Basill-oma | BCCClass | Basosquamous |
| Basill- oma | BCCClass | Basosquamous |
| Basiloma | BCCClass | Basosquamous |
| Basil -oma | BCCClass | Basosquamous |
| Basil-oma | BCCClass | Basosquamous |
| Basil- oma | BCCClass | Basosquamous |
| Basisquamous | BCCClass | Basosquamous |
| Basi squamous | BCCClass | Basosquamous |
| Basi -squamous | BCCClass | Basosquamous |
| Basi - squamous | BCCClass | Basosquamous |
| Basi-squamous | BCCClass | Basosquamous |
| Basi- squamous | BCCClass | Basosquamous |
| Basosquamous | BCCClass | Basosquamous |
| Baso squamous | BCCClass | Basosquamous |
| Baso -squamous | BCCClass | Basosquamous |
| Baso - squamous | BCCClass | Basosquamous |
| Baso-squamous | BCCClass | Basosquamous |
| Baso- squamous | BCCClass | Basosquamous |
| Basosquamous cell carcinoma | BCCClass | Basosquamous |
| Baso squamous cell carcinoma | BCCClass | Basosquamous |
| Baso -squamous cell carcinoma | BCCClass | Basosquamous |
| Baso-squamous cell carcinoma | BCCClass | Basosquamous |
| Baso- squamous cell carcinoma | BCCClass | Basosquamous |
| Cystic | BCCClass | Cystic |
| Desmoplastic | BCCClass | Morphoeic |
| Fibroepithelial | BCCClass | Fibroepithelial |
| Fibro epithelial | BCCClass | Fibroepithelial |
| Fibro -epithelial | BCCClass | Fibroepithelial |
| Fibro - epithelial | BCCClass | Fibroepithelial |
| Fibro-epithelial | BCCClass | Fibroepithelial |
| Fibro- epithelial | BCCClass | Fibroepithelial |
| Fibroepithelioma of pinkus | BCCClass | Fibroepithelial |
| Infiltrating | BCCClass | Infiltrative |
| Infiltrating (high risk) | BCCClass | Infiltrative |
| Infiltrative | BCCClass | Infiltrative |
| Infiltrative (high risk) | BCCClass | Infiltrative |
| Infiltrative (high-risk) | BCCClass | Infiltrative |
| Micronodular | BCCClass | Micronodular |
| Micro nodular | BCCClass | Micronodular |
| Micro -nodular | BCCClass | Micronodular |
| Micro - nodular | BCCClass | Micronodular |
| Micro-nodular | BCCClass | Micronodular |
| Micro- nodular | BCCClass | Micronodular |
| Mixed | BCCClass | Mixed |
| Morphea | BCCClass | Morphoeic |
| Morphea (high risk) | BCCClass | Morphoeic |
| Morphea (high-risk) | BCCClass | Morphoeic |
| Morpheic | BCCClass | Morphoeic |
| Morpheic (high risk) | BCCClass | Morphoeic |
| Morpheic (high-risk) | BCCClass | Morphoeic |
| Morphoea | BCCClass | Morphoeic |
| Morphoea (high risk) | BCCClass | Morphoeic |
| Morphoea (high-risk) | BCCClass | Morphoeic |
| Morphoeic | BCCClass | Morphoeic |
| Morphoeic (high risk) | BCCClass | Morphoeic |
| Morphoeic (high-risk) | BCCClass | Morphoeic |
| Multicentric | BCCClass | Multifocal |
| Multi centric | BCCClass | Superficial |
| Multi -centric | BCCClass | Multifocal |
| Multi - centric | BCCClass | Multifocal |
| Multi-centric | BCCClass | Multifocal |
| Multi- centric | BCCClass | Multifocal |
| Multifocal | BCCClass | Multifocal |
| Multi focal | BCCClass | Multifocal |
| Multi -focal | BCCClass | Multifocal |
| Multi - focal | BCCClass | Multifocal |
| Multi-focal | BCCClass | Multifocal |
| Multi- focal | BCCClass | Multifocal |
| Multinodular | BCCClass | Multinodular |
| Multi Nodular | BCCClass | Multifocal |
| Multi -Nodular | BCCClass | Multinodular |
| Multi - Nodular | BCCClass | Multinodular |
| Multi-Nodular | BCCClass | Multinodular |
| Multi- Nodular | BCCClass | Multinodular |
| Nodular | BCCClass | Nodular |
| Nodulocystic | BCCClass | Nodulocystic |
| Nodulo cystic | BCCClass | Nodulocystic |
| Nodulo-cystic | BCCClass | Nodulocystic |
| Pigment | BCCClass | Pigmented |
| Pigmented | BCCClass | Pigmented |
| Polypoid | BCCClass | Polypoid |
| Sclerosing | BCCClass | Morphoeic |
| Sclerotic | BCCClass | Morphoeic |
| Squamous/basal cell carcinoma | BCCClass | Basosquamous |
| Superficial | BCCClass | Superficial |
| Superficial multifocal | BCCClass | Superficial |

## *BCCClassLinkTerms.lst*

| **Value** |
| --- |
| Containing |
| That has a |

## *BCCStage.lst*

| **Feature 1** | **Value 1** | **Feature 1** |
| --- | --- | --- |
| pT1 | BCCStage | pT1 |
| pT1a | BCCStage | pT1 |
| pT1b | BCCStage | pT1 |
| pT2 | BCCStage | pT2 |
| pT2a | BCCStage | pT2 |
| pT2b | BCCStage | pT2 |
| pT3 | BCCStage | pT3 |
| pT3a | BCCStage | pT3 |
| pT3b | BCCStage | pT3 |
| pT4 | BCCStage | pT4 |
| pT4a | BCCStage | pT4a |
| pT4b | BCCStage | pT4b |

## *BiopsyProven.lst*

| **Value** |
| --- |
| Biopsy proven |
| Previous biopsy |
| Previous Curettage |
| Previous Incision Biopsy |
| Previous Incision Bx |
| Previous Punch |
| Previous Punch Biopsy |
| Previous Punch Bx |
| Previous Shave |
| Previous Shave Biopsy |
| Previous Shave Bx |
| Proven on biopsy |

## *BodyParts.lst*

| **Value** | **Feature 1** | **Value 1** |
| --- | --- | --- |
| Abdomen | BodyPart | Abdomen |
| Abdominal | BodyPart | Abdomen |
| Abdominal wall | BodyPart | Abdomen |
| Abdomninal | BodyPart | Abdomen |
| Abdomninal wall | BodyPart | Abdomen |
| Abductor digiti minimi | BodyPart | Foot |
| Abductor hallucis | BodyPart | Foot |
| Abductor pollicis brevis | BodyPart | Hand |
| Abductor pollicis longus | BodyPart | Arm |
| ACF | BodyPart | Arm |
| Adductor brevis | BodyPart | Thigh |
| Adductor hallucis | BodyPart | Foot |
| Adductor longus | BodyPart | Thigh |
| Adductor magnus | BodyPart | Thigh |
| Adductor pollicis | BodyPart | Hand |
| Adductors | BodyPart | Thigh |
| ADM | BodyPart | Foot |
| Ala | BodyPart | Nose |
| Ala base | BodyPart | Nose |
| Ala lobule | BodyPart | Nose |
| Alar | BodyPart | Nose |
| Alar base | BodyPart | Nose |
| Alar lobule | BodyPart | Nose |
| Ampulla | BodyPart | MedialCanthus |
| Anal | BodyPart | Genito-urinary |
| Anal margin | BodyPart | Genito-urinary |
| Anatomical snuffbox | BodyPart | Hand |
| Anconeus | BodyPart | Arm |
| Ankle | BodyPart | Leg |
| Antecubital fossa | BodyPart | Arm |
| Ante cubital fossa | BodyPart | Arm |
| Ante -cubital fossa | BodyPart | Arm |
| Ante - cubital fossa | BodyPart | Arm |
| Ante- cubital fossa | BodyPart | Arm |
| Anterior abdominal wall | BodyPart | Abdomen |
| Anterior chest | BodyPart | Chest |
| Anterior chestwall | BodyPart | Chest |
| Anterior chest wall | BodyPart | Chest |
| Anterior triangle neck | BodyPart | Neck |
| Anterior triangle neck | BodyPart | Neck |
| Antihelical rim | BodyPart | Ear |
| Anti helical rim | BodyPart | Ear |
| Anti -helical rim | BodyPart | Ear |
| Anti - helical rim | BodyPart | Ear |
| Anti-helical rim | BodyPart | Ear |
| Anti- helical rim | BodyPart | Ear |
| Antihelix | BodyPart | Ear |
| Anti helix | BodyPart | Ear |
| Anti -helix | BodyPart | Ear |
| Anti - helix | BodyPart | Ear |
| Anti-helix | BodyPart | Ear |
| Anti- helix | BodyPart | Ear |
| Anus | BodyPart | Genito-urinary |
| APB | BodyPart | Hand |
| APL | BodyPart | Hand |
| Areolar | BodyPart | Chest |
| Arm | BodyPart | Arm |
| Articularis genu | BodyPart | Thigh |
| Arytenoid | BodyPart | Neck |
| Auricular | BodyPart | Ear |
| Auricularis | BodyPart | Ear |
| Auricular sulcus | BodyPart | Ear |
| Axillary tail | BodyPart | Chest |
| Back | BodyPart | Back |
| Biceps | BodyPart | Arm |
| Biceps femoris | BodyPart | Thigh |
| Brachialis | BodyPart | Arm |
| Brachioradialis | BodyPart | Arm |
| Breast | BodyPart | Chest |
| Buccinator | BodyPart | Cheek |
| Buttock | BodyPart | Back |
| Buttocks | BodyPart | Back |
| Calcaneum | BodyPart | Foot |
| Calf | BodyPart | Leg |
| Canaliculi | BodyPart | MedialCanthus |
| Canaliculus | BodyPart | MedialCanthus |
| Capitate | BodyPart | Hand |
| Carotid triangle | BodyPart | Neck |
| Carpal tunnel | BodyPart | Hand |
| Cervical | BodyPart | Back |
| Cervical spine | BodyPart | Neck |
| Cervical vertebra | BodyPart | Neck |
| Cheek | BodyPart | Cheek |
| Chest | BodyPart | Chest |
| Chin | BodyPart | Chin |
| Chondroglossus | BodyPart | Neck |
| Clavicle | BodyPart | Chest |
| Coccygeal | BodyPart | Back |
| Coccyx | BodyPart | Back |
| Collarbone | BodyPart | Chest |
| Collar bone | BodyPart | Chest |
| Collar -bone | BodyPart | Chest |
| Collar - bone | BodyPart | Chest |
| Collar-bone | BodyPart | Chest |
| Collar- bone | BodyPart | Chest |
| Columella | BodyPart | Nose |
| Commissure | BodyPart | Lip |
| Concha | BodyPart | Ear |
| Conchal bowl | BodyPart | Ear |
| Conjunctiva | BodyPart | Eyelid |
| Coracobrachialis | BodyPart | Arm |
| Corpus | BodyPart | Genito-urinary |
| Corrugator | BodyPart | Forehead |
| Costal | BodyPart | Chest |
| Cricoarytenoid | BodyPart | Neck |
| Cricothyroid | BodyPart | Neck |
| Crura | BodyPart | Ear |
| Crura of antihelix | BodyPart | Ear |
| Crura of anti helix | BodyPart | Ear |
| Crura of anti -helix | BodyPart | Ear |
| Crura of anti - helix | BodyPart | Ear |
| Crura of anti-helix | BodyPart | Ear |
| Crura of anti- helix | BodyPart | Ear |
| Crus | BodyPart | Ear |
| Crus of helix | BodyPart | Ear |
| Cubital fossa | BodyPart | Arm |
| Cuboid | BodyPart | Foot |
| Cuneiform | BodyPart | Foot |
| Cymba | BodyPart | Ear |
| Deltoid | BodyPart | Arm |
| Depressor anguli oris | BodyPart | Chin |
| Depressor labii inferioris | BodyPart | Chin |
| Depressor septi nasi | BodyPart | Nose |
| Depressor supercilii | BodyPart | Forehead |
| Digastric | BodyPart | Neck |
| Digastric triangle | BodyPart | Neck |
| Digit | BodyPart | Hand |
| Dilatator naris | BodyPart | Nose |
| DIPJ | BodyPart | Hand |
| Distal interphalangeal joint | BodyPart | Hand |
| Distal RadialUlnar Joint | BodyPart | Hand |
| Distal Radial Ulnar Joint | BodyPart | Hand |
| Distal -Radial -Ulnar -Joint | BodyPart | Hand |
| Distal - Radial - Ulnar - Joint | BodyPart | Hand |
| Distal-Radial-Ulnar-Joint | BodyPart | Hand |
| Distal- Radial- Ulnar- Joint | BodyPart | Hand |
| Distal RadioUlnar Joint | BodyPart | Hand |
| Distal -Radio -Ulnar -Joint | BodyPart | Hand |
| Distal - Radio - Ulnar - Joint | BodyPart | Hand |
| Distal-Radio-Ulnar-Joint | BodyPart | Hand |
| Distal- Radio- Ulnar- Joint | BodyPart | Hand |
| Dorsal interossei | BodyPart | Hand |
| Dorsal nose | BodyPart | Nose |
| Dorsum nose | BodyPart | Nose |
| DRUJ | BodyPart | Hand |
| Dry mucosa | BodyPart | Lip |
| EAM | BodyPart | Ear |
| Ear | BodyPart | Ear |
| Earlobe | BodyPart | Ear |
| Ear lobe | BodyPart | Ear |
| ECR | BodyPart | Arm |
| ECRB | BodyPart | Arm |
| ECRL | BodyPart | Arm |
| EDB | BodyPart | Arm |
| EDL | BodyPart | Arm |
| EDM | BodyPart | Arm |
| EHB | BodyPart | Arm |
| EHL | BodyPart | Arm |
| EI | BodyPart | Arm |
| EIP | BodyPart | Arm |
| Elbow | BodyPart | Arm |
| EPB | BodyPart | Arm |
| Epigastric | BodyPart | Abdomen |
| Epigastrium | BodyPart | Abdomen |
| EPL | BodyPart | Arm |
| Eponychial fold | BodyPart | NailComplex |
| Eponychium | BodyPart | NailComplex |
| Erector spinae | BodyPart | Back |
| Extensor carpi radialis brevis | BodyPart | Arm |
| Extensor carpi radialis longus | BodyPart | Arm |
| Extensor carpi ulnaris | BodyPart | Arm |
| Extensor digiti minimi | BodyPart | Arm |
| Extensor digitorum | BodyPart | Arm |
| Extensor digitorum brevis | BodyPart | Foot |
| Extensor digitorum longus | BodyPart | Leg |
| Extensor hallucis brevis | BodyPart | Foot |
| Extensor hallucis longus | BodyPart | Leg |
| Extensor indicis | BodyPart | Arm |
| Extensor pollicis brevis | BodyPart | Arm |
| Extensor pollicis longus | BodyPart | Arm |
| External auditory meatus | BodyPart | Ear |
| External oblique | BodyPart | Abdomen |
| Eyebrow | BodyPart | Eyebrow |
| Eye brow | BodyPart | Eyebrow |
| Eye brow | BodyPart | Eyebrow |
| Eyelid | BodyPart | Eyelid |
| Eye lid | BodyPart | Eyelid |
| Eye -lid | BodyPart | Eyelid |
| Eye - lid | BodyPart | Eyelid |
| Eye-lid | BodyPart | Eyelid |
| Eye- lid | BodyPart | Eyelid |
| Face | BodyPart | Face |
| Facial | BodyPart | Face |
| FCR | BodyPart | Arm |
| FCU | BodyPart | Arm |
| FDP | BodyPart | Arm |
| FDS | BodyPart | Arm |
| Femoral | BodyPart | Thigh |
| Femur | BodyPart | Thigh |
| Fibula | BodyPart | Leg |
| Finger | BodyPart | Hand |
| Finger tip | BodyPart | Hand |
| Flank | BodyPart | Abdomen |
| Flexor carpi radialis | BodyPart | Arm |
| Flexor carpi ulnaris | BodyPart | Arm |
| Flexor digiti minimi brevis | BodyPart | Foot |
| Flexor digiti minimi brevis | BodyPart | Hand |
| Flexor digitorum brevis | BodyPart | Foot |
| Flexor digitorum longus | BodyPart | Leg |
| Flexor digitorum profundus | BodyPart | Arm |
| Flexor digitorum superficialis | BodyPart | Arm |
| Flexor hallucis brevis | BodyPart | Foot |
| Flexor hallucis longus | BodyPart | Leg |
| Flexor pollicis brevis | BodyPart | Hand |
| Flexor pollicis longus | BodyPart | Arm |
| Foot | BodyPart | Foot |
| Forearm | BodyPart | Arm |
| Forefoot | BodyPart | Foot |
| Forehead | BodyPart | Forehead |
| Fore head | BodyPart | Forehead |
| FPB | BodyPart | Arm |
| FPL | BodyPart | Arm |
| Gastrocnemius | BodyPart | Leg |
| Gemelli | BodyPart | Thigh |
| Gemellus | BodyPart | Thigh |
| Genioglossus | BodyPart | Neck |
| Geniohyoid | BodyPart | Neck |
| Genito-urinary | BodyPart | Genito-urinary |
| Glabellar | BodyPart | Forehead |
| Glabellar area | BodyPart | Forehead |
| Glabellar region | BodyPart | Forehead |
| Glans | BodyPart | Genito-urinary |
| Gluteal | BodyPart | Back |
| Gluteus | BodyPart | Back |
| Gluteus maximus muscle | BodyPart | Thigh |
| Gluteus medius muscle | BodyPart | Thigh |
| Gracilis | BodyPart | Thigh |
| Greater Trochanter | BodyPart | Thigh |
| Great toe | BodyPart | Foot |
| Grey line | BodyPart | Eyelid |
| Groin | BodyPart | Leg |
| Hairline | BodyPart | Scalp |
| Hair line | BodyPart | Scalp |
| Hamate | BodyPart | Hand |
| Hand | BodyPart | Hand |
| Heel | BodyPart | Foot |
| Helical | BodyPart | Ear |
| Helical rim | BodyPart | Ear |
| Helix | BodyPart | Ear |
| Helix rim | BodyPart | Ear |
| Hindfoot | BodyPart | Foot |
| Hip | BodyPart | Thigh |
| Humerus | BodyPart | Arm |
| Hyoglossus | BodyPart | Neck |
| Hyoid | BodyPart | Neck |
| Hypochondriac | BodyPart | Abdomen |
| Hypochondrial | BodyPart | Abdomen |
| Hypochondric | BodyPart | Abdomen |
| Hypochondrium | BodyPart | Abdomen |
| Hypogastric | BodyPart | Abdomen |
| Hypogastrium | BodyPart | Abdomen |
| Hyponychial fold | BodyPart | NailComplex |
| Hyponychium | BodyPart | NailComplex |
| Hypothenar eminence | BodyPart | Hand |
| Hypo-thenar eminenceBodyPart=Hand |  |  |
| Iliac | BodyPart | Abdomen |
| Iliacus | BodyPart | Thigh |
| Iliocostalis | BodyPart | Back |
| Iliopsoas | BodyPart | Thigh |
| Ilium | BodyPart | Abdomen |
| Illiac | BodyPart | Abdomen |
| Illiac fossa | BodyPart | Abdomen |
| Infraclavicular | BodyPart | Chest |
| Infra clavicular | BodyPart | Chest |
| Infra-clavicular | BodyPart | Chest |
| Inframammary fold | BodyPart | Chest |
| Infra mammary fold | BodyPart | Chest |
| Infra -mammary fold | BodyPart | Chest |
| Infra - mammary fold | BodyPart | Chest |
| Infra- mammary fold | BodyPart | Chest |
| Infraorbital | BodyPart | Cheek |
| Infra -orbital | BodyPart | Cheek |
| Infra - orbital | BodyPart | Cheek |
| Infra-orbital | BodyPart | Cheek |
| Infra- orbital | BodyPart | Cheek |
| Infraorbital rim | BodyPart | Cheek |
| Infra-orbital rim | BodyPart | Cheek |
| Infraspinatus | BodyPart | Arm |
| Infra temporal fossa | BodyPart | Temple |
| Infra -temporal fossa | BodyPart | Temple |
| Infra - temporal fossa | BodyPart | Temple |
| Infra-temporal fossa | BodyPart | Temple |
| Infra- temporal fossa | BodyPart | Temple |
| Inguinal | BodyPart | Thigh |
| Intercostal | BodyPart | Back |
| Intercostals | BodyPart | Back |
| Internal oblique | BodyPart | Abdomen |
| Interphalangeal joint | BodyPart | Hand |
| Inter phalangeal joint | BodyPart | Hand |
| Inter -phalangeal joint | BodyPart | Hand |
| Inter - phalangeal joint | BodyPart | Hand |
| Inter-phalangeal joint | BodyPart | Hand |
| Inter- phalangeal joint | BodyPart | Hand |
| Interspinales | BodyPart | Back |
| Intertransversarii | BodyPart | Back |
| IPJ | BodyPart | Hand |
| Ischial | BodyPart | Thigh |
| Ischial | BodyPart | Thigh |
| Ischium | BodyPart | Thigh |
| Knee | BodyPart | Leg |
| Labia | BodyPart | Genito-urinary |
| Labial | BodyPart | Genito-urinary |
| Labia majora | BodyPart | Genito-urinary |
| Labia minora | BodyPart | Genito-urinary |
| Lacrimal duct | BodyPart | MedialCanthus |
| Lacrimal sac | BodyPart | MedialCanthus |
| Lateral canthus | BodyPart | LateralCanthus |
| Lateral malleolus | BodyPart | Leg |
| Lateral nail fold | BodyPart | NailComplex |
| Lateral nasal wall | BodyPart | Nose |
| Lateral wall | BodyPart | Nose |
| Latissimus dorsi | BodyPart | Back |
| Leg | BodyPart | Leg |
| Lesser Trochanter | BodyPart | Thigh |
| Levator anguli oris | BodyPart | Cheek |
| Levator ani | BodyPart | Genito-urinary |
| Levatores costarum | BodyPart | Back |
| Levator labii superioris | BodyPart | Cheek |
| Levator scapulae | BodyPart | Neck |
| Levator veli palatini | BodyPart | Neck |
| Level 1 neck | BodyPart | Neck |
| Level 1 neck | BodyPart | Neck |
| Level 2 neck | BodyPart | Neck |
| Level 2 neck | BodyPart | Neck |
| Level 3 neck | BodyPart | Neck |
| Level 3 neck | BodyPart | Neck |
| Level 4 neck | BodyPart | Neck |
| Level 4 neck | BodyPart | Neck |
| Level 5 neck | BodyPart | Neck |
| Level 5 neck | BodyPart | Neck |
| Level III neck | BodyPart | Neck |
| Level III neck | BodyPart | Neck |
| Level II neck | BodyPart | Neck |
| Level II neck | BodyPart | Neck |
| Level I neck | BodyPart | Neck |
| Level I neck | BodyPart | Neck |
| Level IV neck | BodyPart | Neck |
| Level IV neck | BodyPart | Neck |
| Level V | BodyPart | Neck |
| Level V | BodyPart | Neck |
| Lip | BodyPart | Lip |
| Lobe | BodyPart | Ear |
| Lobule | BodyPart | Ear |
| Longissimus | BodyPart | Back |
| Longissimus capitis | BodyPart | Neck |
| Lower lip | BodyPart | Lip |
| Lumbar | BodyPart | Back |
| Lumbar spine | BodyPart | Back |
| Lumbar vertebra | BodyPart | Back |
| Lumbar vertebrae | BodyPart | Back |
| Lumbrical | BodyPart | Hand |
| Lumbricals | BodyPart | Hand |
| Lunate | BodyPart | Hand |
| Malar | BodyPart | Cheek |
| Malleolus | BodyPart | Leg |
| Mandible | BodyPart | Chin |
| Manubrium | BodyPart | Chest |
| Masseter | BodyPart | Cheek |
| Mastoid | BodyPart | Neck |
| Mastoid area | BodyPart | Neck |
| Mastoid process | BodyPart | Neck |
| Mastoid region | BodyPart | Neck |
| Maxilla | BodyPart | Cheek |
| MCPJ | BodyPart | Hand |
| Medial canthus | BodyPart | MedialCanthus |
| Medial Malleolus | BodyPart | Leg |
| Melolabial | BodyPart | Cheek |
| Melo labial | BodyPart | Cheek |
| Melo -labial | BodyPart | Cheek |
| Melo - labial | BodyPart | Cheek |
| Melo-labial | BodyPart | Cheek |
| Melo- labial | BodyPart | Cheek |
| Mental | BodyPart | Chin |
| Mentalis | BodyPart | Chin |
| Metacarpal | BodyPart | Hand |
| metacarpophalangeal joint | BodyPart | Hand |
| Metatarsal | BodyPart | Foot |
| Meta tarso phalangeal joint | BodyPart | Foot |
| Meta -tarso -phalangeal joint | BodyPart | Foot |
| Meta - tarso - phalangeal joint | BodyPart | Foot |
| Meta-tarso-phalangeal joint | BodyPart | Foot |
| Meta- tarso- phalangeal joint | BodyPart | Foot |
| Midfoot | BodyPart | Foot |
| Mobile wad | BodyPart | Arm |
| Mons | BodyPart | Abdomen |
| Mons pubis | BodyPart | Abdomen |
| MTPJ | BodyPart | Foot |
| Multifidus | BodyPart | Back |
| Muscular triangle | BodyPart | Neck |
| Mylohyoid | BodyPart | Neck |
| NAC | BodyPart | Chest |
| Nail | BodyPart | NailComplex |
| Nail | BodyPart | NailComplex |
| Nail bed | BodyPart | NailComplex |
| NailComplex | BodyPart | NailComplex |
| Nail Complex | BodyPart | NailComplex |
| Nasal | BodyPart | Nose |
| Nasalis | BodyPart | Nose |
| Nasal sidewall | BodyPart | Nose |
| Nasal side wall | BodyPart | Nose |
| Nasal side -wall | BodyPart | Nose |
| Nasal side - wall | BodyPart | Nose |
| Nasal side-wall | BodyPart | Nose |
| Nasal side- wall | BodyPart | Nose |
| Nasal tip | BodyPart | Nose |
| Nasal wall | BodyPart | Nose |
| Nasojugal | BodyPart | Cheek |
| Naso jugal | BodyPart | Cheek |
| Naso -jugal | BodyPart | Cheek |
| Naso - jugal | BodyPart | Cheek |
| Naso-jugal | BodyPart | Cheek |
| Naso- jugal | BodyPart | Cheek |
| Nasolabial | BodyPart | Cheek |
| Naso labial | BodyPart | Cheek |
| Naso -labial | BodyPart | Cheek |
| Naso - labial | BodyPart | Cheek |
| Naso-labial | BodyPart | Cheek |
| Naso- labial | BodyPart | Cheek |
| Nasolacrimal duct | BodyPart | MedialCanthus |
| Navel | BodyPart | Abdomen |
| Navicular | BodyPart | Foot |
| Neck | BodyPart | Neck |
| Nipple | BodyPart | Chest |
| Nipple-areola complex | BodyPart | Chest |
| Nipple areolar complex | BodyPart | Chest |
| Nipple -areolar -complex | BodyPart | Chest |
| Nipple - areolar complex | BodyPart | Chest |
| Nipple - areolar - complex | BodyPart | Chest |
| Nipple - areolar- complex | BodyPart | Chest |
| Nipple-areolar-complex | BodyPart | Chest |
| Nipple- areolar complex | BodyPart | Chest |
| Nipple- areolar- complex | BodyPart | Chest |
| Nose | BodyPart | Nose |
| Obliquus capitis superior | BodyPart | Neck |
| Obturator externus | BodyPart | Thigh |
| Obturator internus | BodyPart | Thigh |
| Omohyoid | BodyPart | Neck |
| Opponens digiti minimi | BodyPart | Hand |
| Opponens pollicis | BodyPart | Hand |
| Oral commissure | BodyPart | Lip |
| Orbicularis oculi | BodyPart | Eyelid |
| Orbicularis oris | BodyPart | Lip |
| Palatoglossus | BodyPart | Neck |
| Palatopharyngeus | BodyPart | Neck |
| Palm | BodyPart | Hand |
| Palmar interossei | BodyPart | Hand |
| Palmaris brevis | BodyPart | Hand |
| Palmaris longus | BodyPart | Arm |
| Paronychial fold | BodyPart | NailComplex |
| Paronychium | BodyPart | NailComplex |
| Patella | BodyPart | Leg |
| Pectineus | BodyPart | Thigh |
| Pectoralis major | BodyPart | Chest |
| Pectoralis minor | BodyPart | Chest |
| Penile | BodyPart | Genito-urinary |
| Penis | BodyPart | Genito-urinary |
| Per auricular | BodyPart | Pre-auricular |
| Per - auricular | BodyPart | Pre -auricular |
| Per - auricular | BodyPart | Pre-auricular |
| Per-auricular | BodyPart | Pre-auricular |
| Per- auricular | BodyPart | Pre-auricular |
| Peri auricular area | BodyPart | Ear |
| Peri -auricular area | BodyPart | Ear |
| Peri - auricular area | BodyPart | Ear |
| Peri-auricular area | BodyPart | Ear |
| Peri- auricular area | BodyPart | Ear |
| Perineum | BodyPart | Genito-urinary |
| Periumbilical | BodyPart | Abdomen |
| Peri umbilical | BodyPart | Abdomen |
| Peri-umbilical | BodyPart | Abdomen |
| Per left auricular | BodyPart | Pre-auricular |
| Per - left - auricular | BodyPart | Pre -auricular |
| Per - left - auricular | BodyPart | Pre-auricular |
| Per-left-auricular | BodyPart | Pre-auricular |
| Per- left- auricular | BodyPart | Pre-auricular |
| Peroneus brevis | BodyPart | Leg |
| Peroneus longus | BodyPart | Leg |
| Peroneus tertius | BodyPart | Leg |
| Per right auricular | BodyPart | Pre-auricular |
| Per - right - auricular | BodyPart | Pre -auricular |
| Per - right - auricular | BodyPart | Pre-auricular |
| Per-right-auricular | BodyPart | Pre-auricular |
| Per- right- auricular | BodyPart | Pre-auricular |
| Philtral | BodyPart | Lip |
| Philtral column | BodyPart | Lip |
| Philtrum | BodyPart | Lip |
| Pinna | BodyPart | Ear |
| PIPJ | BodyPart | Hand |
| Piriformis | BodyPart | Thigh |
| Pisiform | BodyPart | Hand |
| Plantar foot | BodyPart | Foot |
| Plantaris | BodyPart | Leg |
| Platysma | BodyPart | Neck |
| Popliteal | BodyPart | Leg |
| Popliteal fossa | BodyPart | Leg |
| Popliteal fossa | BodyPart | Leg |
| Popliteus | BodyPart | Leg |
| Postauricular | BodyPart | Post-auricular |
| Postauricular | BodyPart | Post-auricular |
| Post auricular | BodyPart | Post-auricular |
| Post auricular | BodyPart | Post-auricular |
| Post - auricular | BodyPart | Post -auricular |
| Post - auricular | BodyPart | Post-auricular |
| Post-auricular | BodyPart | Post-auricular |
| Post-auricular | BodyPart | Post-auricular |
| Post- auricular | BodyPart | Post-auricular |
| Postauricular area | BodyPart | Post-auricular |
| Post-auricular area | BodyPart | Post-auricular |
| Postauricular region | BodyPart | Post-auricular |
| Post-auricular region | BodyPart | Post-auricular |
| Postauricular sulcus | BodyPart | Post-auricular |
| Post auricular sulcus | BodyPart | Post-auricular |
| Post-auricular sulcus | BodyPart | Post-auricular |
| Posterior auricular | BodyPart | Post-auricular |
| Posterior-auricular | BodyPart | Post-auricular |
| Posterior auricular sulcus | BodyPart | Post-auricular |
| Posterior-auricular sulcus | BodyPart | Post-auricular |
| Posterior chest | BodyPart | Back |
| Posterior chestwall | BodyPart | Back |
| Posterior chest wall | BodyPart | Back |
| Posterior triangle neck | BodyPart | Neck |
| Posterior triangle neck | BodyPart | Neck |
| Post left auricular | BodyPart | Post-auricular |
| Post - left - auricular | BodyPart | Post -auricular |
| Post - left - auricular | BodyPart | Post-auricular |
| Post-left-auricular | BodyPart | Post-auricular |
| Post- left- auricular | BodyPart | Post-auricular |
| Post right auricular | BodyPart | Post-auricular |
| Post - right - auricular | BodyPart | Post -auricular |
| Post - right - auricular | BodyPart | Post-auricular |
| Post-right-auricular | BodyPart | Post-auricular |
| Post- right- auricular | BodyPart | Post-auricular |
| Preauricular | BodyPart | Pre-auricular |
| Preauricular | BodyPart | Pre-auricular |
| Pre auricular | BodyPart | Pre-auricular |
| Pre auricular | BodyPart | Pre-auricular |
| Pre - auricular | BodyPart | Pre -auricular |
| Pre - auricular | BodyPart | Pre-auricular |
| Pre-auricular | BodyPart | Pre-auricular |
| Pre-auricular | BodyPart | Pre-auricular |
| Pre- auricular | BodyPart | Pre-auricular |
| Preauricular area | BodyPart | Pre-auricular |
| Pre auricular area | BodyPart | Pre-auricular |
| Pre-auricular area | BodyPart | Pre-auricular |
| Preauricular region | BodyPart | Pre-auricular |
| Pre auricular region | BodyPart | Pre-auricular |
| Pre-auricular region | BodyPart | Pre-auricular |
| Preauricular sulcus | BodyPart | Pre-auricular |
| Pre auricular sulcus | BodyPart | Pre-auricular |
| Pre-auricular sulcus | BodyPart | Pre-auricular |
| Pre left auricular | BodyPart | Pre-auricular |
| Pre - left auricular | BodyPart | Pre -auricular |
| Pre - left - auricular | BodyPart | Pre-auricular |
| Pre-left-auricular | BodyPart | Pre-auricular |
| Pre- left auricular | BodyPart | Pre-auricular |
| Pre right auricular | BodyPart | Pre-auricular |
| Pre - right auricular | BodyPart | Pre -auricular |
| Pre - right - auricular | BodyPart | Pre-auricular |
| Pre-right-auricular | BodyPart | Pre-auricular |
| Pre- right auricular | BodyPart | Pre-auricular |
| Pretibia | BodyPart | Leg |
| Pre tibia | BodyPart | Leg |
| Pre -tibia | BodyPart | Leg |
| Pre - tibia | BodyPart | Leg |
| Pre-tibia | BodyPart | Leg |
| Pre- tibia | BodyPart | Leg |
| Pretibial | BodyPart | Leg |
| Pretibial | BodyPart | Leg |
| Pre tibial | BodyPart | Leg |
| Pre-tibial | BodyPart | Leg |
| Procerus | BodyPart | Forehead |
| Pronator quadratus | BodyPart | Arm |
| Pronator teres | BodyPart | Arm |
| Proximal interphalangeal joint | BodyPart | Hand |
| Psoas major | BodyPart | Thigh |
| Psoas minor | BodyPart | Thigh |
| Pterygoid | BodyPart | Cheek |
| Pterygopalatine fossa | BodyPart | Temple |
| Pterygo palatine fossa | BodyPart | Temple |
| Pterygo -palatine fossa | BodyPart | Temple |
| Pterygo - palatine fossa | BodyPart | Temple |
| Pterygo-palatine fossa | BodyPart | Temple |
| Pterygo- palatine fossa | BodyPart | Temple |
| Pubic | BodyPart | Abdomen |
| Punctum | BodyPart | MedialCanthus |
| Pyramidalis | BodyPart | Abdomen |
| Quadratus femoris | BodyPart | Thigh |
| Quadratus plantae | BodyPart | Foot |
| Quadriceps femoris | BodyPart | Thigh |
| Radius | BodyPart | Arm |
| Ray | BodyPart | Hand |
| Rectus abdominis | BodyPart | Abdomen |
| Rectus capitis | BodyPart | Neck |
| Rectus capitis lateralis | BodyPart | Neck |
| Rectus femoris | BodyPart | Thigh |
| Rhomboid major | BodyPart | Back |
| Rhomboid minor | BodyPart | Back |
| Rhomboids | BodyPart | Back |
| Rib | BodyPart | Chest |
| Ribs | BodyPart | Chest |
| Risorius | BodyPart | Cheek |
| Rotator cuff | BodyPart | Arm |
| Rotatores | BodyPart | Back |
| Sacral | BodyPart | Back |
| Sacral spine | BodyPart | Back |
| Sacral vertebra | BodyPart | Back |
| Sacral vertebrae | BodyPart | Back |
| Sacrum | BodyPart | Back |
| Salpingopharyngeus | BodyPart | Neck |
| Sartorius | BodyPart | Thigh |
| Scalene | BodyPart | Neck |
| Scalenes | BodyPart | Neck |
| Scalp | BodyPart | Scalp |
| Scaphoid | BodyPart | Hand |
| Scapula | BodyPart | Back |
| Scrotal | BodyPart | Genito-urinary |
| Scrotum | BodyPart | Genito-urinary |
| Semimembranosus | BodyPart | Thigh |
| Semispinalis | BodyPart | Back |
| Semispinalis capitis | BodyPart | Neck |
| Semitendinosus | BodyPart | Thigh |
| Serratus | BodyPart | Chest |
| Shin | BodyPart | Leg |
| Shoulder | BodyPart | Arm |
| Soft triangle | BodyPart | Nose |
| Soleus | BodyPart | Leg |
| Sphenoid | BodyPart | Temple |
| Spinalis | BodyPart | Back |
| Splenius capitis | BodyPart | Back |
| Splenius capitis | BodyPart | Neck |
| Splenius cervicis | BodyPart | Back |
| Stapedius | BodyPart | Ear |
| Sternocleidomastoid | BodyPart | Neck |
| Sternohyoid | BodyPart | Neck |
| Sternothyroid | BodyPart | Neck |
| Sternum | BodyPart | Chest |
| Styloglossus | BodyPart | Neck |
| Stylohyoid | BodyPart | Neck |
| Stylopharyngeus | BodyPart | Neck |
| Subclavius | BodyPart | Chest |
| Subcostal | BodyPart | Back |
| Subcostals | BodyPart | Back |
| Submandibular | BodyPart | Neck |
| Sub mandibular | BodyPart | Neck |
| Sub -mandibular | BodyPart | Neck |
| Sub - mandibular | BodyPart | Neck |
| Sub-mandibular | BodyPart | Neck |
| Sub- mandibular | BodyPart | Neck |
| Submental | BodyPart | Neck |
| Sub mental | BodyPart | Neck |
| Sub -mental | BodyPart | Neck |
| Sub - mental | BodyPart | Neck |
| Sub-mental | BodyPart | Neck |
| Sub- mental | BodyPart | Neck |
| Subscapularis | BodyPart | Arm |
| Supinator | BodyPart | Arm |
| Supraclavicular | BodyPart | Neck |
| Supraclavicular fossa | BodyPart | Neck |
| Supra clavicular fossa | BodyPart | Neck |
| Supra -clavicular fossa | BodyPart | Neck |
| Supra - clavicular fossa | BodyPart | Neck |
| Supra-clavicular fossa | BodyPart | Neck |
| Supra- clavicular fossa | BodyPart | Neck |
| Supra-clavicular neck | BodyPart | Neck |
| Suprahyoid | BodyPart | Neck |
| Supra hyoid | BodyPart | Neck |
| Supra -hyoid | BodyPart | Neck |
| Supra - hyoid | BodyPart | Neck |
| Supra-hyoid | BodyPart | Neck |
| Supra- hyoid | BodyPart | Neck |
| Supra orbital | BodyPart | Forehead |
| Supra -orbital | BodyPart | Forehead |
| Supra - orbital | BodyPart | Forehead |
| Supra-orbital | BodyPart | Forehead |
| Supra- orbital | BodyPart | Forehead |
| Supra orbital rim | BodyPart | Forehead |
| Supra-orbital rim | BodyPart | Forehead |
| Suprapubic | BodyPart | Abdomen |
| Supra pubic | BodyPart | Abdomen |
| Supra -pubic | BodyPart | Abdomen |
| Supra - pubic | BodyPart | Abdomen |
| Supra- pubic | BodyPart | Abdomen |
| Supraspinatus | BodyPart | Arm |
| Supratip | BodyPart | Nose |
| Supra tip | BodyPart | Nose |
| Supra -tip | BodyPart | Nose |
| Supra - tip | BodyPart | Nose |
| Supra-tip | BodyPart | Nose |
| Supra- tip | BodyPart | Nose |
| Talus | BodyPart | Foot |
| Tarsal plate | BodyPart | Eyelid |
| Tarsus | BodyPart | Eyelid |
| Tear trough | BodyPart | Cheek |
| Temple | BodyPart | Temple |
| Temporal fossa | BodyPart | Temple |
| Temporalis | BodyPart | Temple |
| Temporoparietalis | BodyPart | Ear |
| Tensor fascia lata | BodyPart | Thigh |
| Tensor fascia latae | BodyPart | Thigh |
| Tensor tympani | BodyPart | Ear |
| Tensor veli palatini | BodyPart | Neck |
| Teres major | BodyPart | Arm |
| Teres minor | BodyPart | Arm |
| Thenar eminence | BodyPart | Hand |
| Thigh | BodyPart | Thigh |
| Thoracic | BodyPart | Back |
| Thoracic spine | BodyPart | Back |
| Thoracic vertebra | BodyPart | Back |
| Thoracic vertebrae | BodyPart | Back |
| Thumb | BodyPart | Hand |
| Thyroarytenoid | BodyPart | Neck |
| Thyrohyoid | BodyPart | Neck |
| Tibia | BodyPart | Leg |
| Tibialis | BodyPart | Leg |
| Tip of nose | BodyPart | Nose |
| Toe | BodyPart | Foot |
| Toes | BodyPart | Foot |
| Tragal notch | BodyPart | Ear |
| Tragus | BodyPart | Ear |
| Transversospinales | BodyPart | Back |
| Transversus abdominis | BodyPart | Abdomen |
| Transversus thoracis | BodyPart | Back |
| Trapezium | BodyPart | Hand |
| Trapezius | BodyPart | Back |
| Trapezoid | BodyPart | Hand |
| Triangular fossa | BodyPart | Ear |
| Triceps | BodyPart | Arm |
| Triquetral | BodyPart | Hand |
| Triquetrum | BodyPart | Hand |
| Trochanter | BodyPart | Thigh |
| Trochanteric | BodyPart | Thigh |
| Ulna | BodyPart | Arm |
| Umbilical | BodyPart | Abdomen |
| Umbilicus | BodyPart | Abdomen |
| Vagina | BodyPart | Genito-urinary |
| Vaginal | BodyPart | Genito-urinary |
| Vastus intermedius | BodyPart | Thigh |
| Vastus lateralis | BodyPart | Thigh |
| Vastus medialis | BodyPart | Thigh |
| Vermilion | BodyPart | Lip |
| Verticalis | BodyPart | Neck |
| Vestibule | BodyPart | Nose |
| Vulva | BodyPart | Genito-urinary |
| Vulval | BodyPart | Genito-urinary |
| Wet mucosa | BodyPart | Lip |
| White roll | BodyPart | Lip |
| Wrist | BodyPart | Hand |
| Xiphisternum | BodyPart | Chest |
| Xiphoid process | BodyPart | Chest |
| Zygoma | BodyPart | Cheek |
| Zygomaticus | BodyPart | Cheek |
| Zygomaticus major | BodyPart | Cheek |
| Zygomaticus minor | BodyPart | Cheek |

## *ClarkLevel.lst*

| **Value** | **Feature 1** | **Value 1** |
| --- | --- | --- |
| Clark's level 1 | Clark'sLevel | I |
| Clark's level 2 | Clark'sLevel | II |
| Clark's level 3 | Clark'sLevel | III |
| Clark's level 4 | Clark'sLevel | IV |
| Clark's level 5 | Clark'sLevel | V |
| Clark's level I | Clark'sLevel | I |
| Clark's level II | Clark'sLevel | II |
| Clark's level III | Clark'sLevel | III |
| Clark's level IV | Clark'sLevel | IV |
| Clark's level V | Clark'sLevel | V |
| Clarke level 1 | ClarkeLevel | I |
| Clarke level 2 | ClarkeLevel | II |
| Clarke level 3 | ClarkeLevel | III |
| Clarke level 4 | ClarkeLevel | IV |
| Clarke level 5 | ClarkeLevel | V |
| Clarke level I | ClarkeLevel | I |
| Clarke level II | ClarkeLevel | II |
| Clarke level III | ClarkeLevel | III |
| Clarke level IV | ClarkeLevel | IV |
| Clarke level V | ClarkeLevel | V |
| Clarkes level 1 | ClarkesLevel | I |
| Clarkes level 2 | ClarkesLevel | II |
| Clarkes level 3 | ClarkesLevel | III |
| Clarkes level 4 | ClarkesLevel | IV |
| Clarkes level 5 | ClarkesLevel | V |
| Clarkes level I | ClarkesLevel | I |
| Clarkes level II | ClarkesLevel | II |
| Clarkes level III | ClarkesLevel | III |
| Clarkes level IV | ClarkesLevel | IV |
| Clarkes level V | ClarkesLevel | V |
| Clark level 1 | ClarkLevel | I |
| Clark level 2 | ClarkLevel | II |
| Clark level 3 | ClarkLevel | III |
| Clark level 4 | ClarkLevel | IV |
| Clark level 5 | ClarkLevel | V |
| Clark level I | ClarkLevel | I |
| Clark level II | ClarkLevel | II |
| Clark level III | ClarkLevel | III |
| Clark level IV | ClarkLevel | IV |
| Clark level V | ClarkLevel | V |
| Clarks level 1 | ClarksLevel | I |
| Clarks level 2 | ClarksLevel | II |
| Clarks level 3 | ClarksLevel | III |
| Clarks level 4 | ClarksLevel | IV |
| Clarks level 5 | ClarksLevel | V |
| Clarks level I | ClarksLevel | I |
| Clarks level II | ClarksLevel | II |
| Clarks level III | ClarksLevel | III |
| Clarks level IV | ClarksLevel | IV |
| Clarks level V | ClarksLevel | V |

## *ClarkLevelOfInvasionTrigger.lst*

| **Value** |
| --- |
| Level of invasion |

## *Clear.lst*

| **Value** | **Feature 1** | **Value 1** |
| --- | --- | --- |
| Negative | Negatives | true |
| Clear of the | Negatives | true |
| Clear | Negatives | true |

## *ClearButClose.lst*

| **Value** | **Feature 1** | **Value 1** |
| --- | --- | --- |
| Clear but close | Negatives | true |

## *ClinicalDetailCleaningTrigger.lst*

| **Value** |
| --- |
| Last |

## *ClinicalDetailMargin.lst*

| **Value** |
| --- |
| Margins |
| Margin |
| Edge |
| Clearance |

## *Close.lst*

| **Value** | **Feature 1** | **Value 1** |
| --- | --- | --- |
| Closest | Negatives | true |
| Close | Negatives | true |

## *DeepMargin.lst*

| **Value** | **Feature 1** | **Value 1** | **Feature 2** | **Value 2** |
| --- | --- | --- | --- | --- |
| Abductor digiti minimi | DeepMargin | Muscle |  |  |
| Abductor digiti minimi | DeepMargin | Muscle |  |  |
| Abductor hallucis | DeepMargin | Muscle |  |  |
| Abductor pollicis brevis | DeepMargin | Muscle |  |  |
| Abductor pollicis longus | DeepMargin | Muscle |  |  |
| Adductor | DeepMargin | Muscle |  |  |
| Adductor brevis | DeepMargin | Muscle |  |  |
| Adductor hallucis | DeepMargin | Muscle |  |  |
| Adductor longus | DeepMargin | Muscle |  |  |
| Adductor magnus | DeepMargin | Muscle |  |  |
| Adductor pollicis | DeepMargin | Muscle |  |  |
| Adductors | DeepMargin | Muscle |  |  |
| ADM | DeepMargin | Muscle |  |  |
| Anconeus | DeepMargin | Muscle |  |  |
| APB | DeepMargin | Muscle |  |  |
| APL | DeepMargin | Muscle |  |  |
| Articularis genu | DeepMargin | Muscle |  |  |
| Biceps | DeepMargin | Muscle |  |  |
| Biceps femoris | DeepMargin | Muscle |  |  |
| Bone | CUI | C0262950 | DeepMargin | Bone |
| Brachialis | DeepMargin | Muscle |  |  |
| Brachioradialis | DeepMargin | Muscle |  |  |
| Buccinator | DeepMargin | Muscle |  |  |
| Calcaneum | DeepMargin | Bone |  |  |
| Camper's fascia | DeepMargin | SuperficialFascia |  |  |
| Camper fascia | DeepMargin | SuperficialFascia |  |  |
| Capitate | DeepMargin | Bone |  |  |
| Cartilage | CUI | C0007301 | DeepMargin | Cartilage |
| Chondroglossus | DeepMargin | Muscle |  |  |
| Clavicle | DeepMargin | Bone |  |  |
| Coccyx | DeepMargin | Bone |  |  |
| Collarbone | DeepMargin | Bone |  |  |
| Coracobrachialis | DeepMargin | Muscle |  |  |
| Corpus | DeepMargin | Muscle |  |  |
| Corrugator | DeepMargin | Muscle |  |  |
| Cortex | CUI | C0262950 | DeepMargin | Bone |
| Cuboid | DeepMargin | Bone |  |  |
| Cuff fat | CUI | C0222331 | DeepMargin | Fat |
| Cuff of fat | CUI | C0222331 | DeepMargin | Fat |
| Cuneiform | DeepMargin | Bone |  |  |
| Deep cervical fascia | DeepMargin | DeepFascia |  |  |
| Deep fascia | DeepMargin | DeepFascia |  |  |
| Deep fascial | DeepMargin | DeepFascia |  |  |
| Deep investing cervical fascia | DeepMargin | DeepFascia |  |  |
| Deep investing layer | DeepMargin | DeepFascia |  |  |
| Deep investing layer cervical fascia | DeepMargin | DeepFascia |  |  |
| Deep investing layer of cervical fascia | DeepMargin | DeepFascia |  |  |
| Deep temporal fascia | DeepMargin | DeepFascia |  |  |
| Deltoid | DeepMargin | Muscle |  |  |
| Depressor | DeepMargin | Muscle |  |  |
| Depressors | DeepMargin | Muscle |  |  |
| Dermal | CUI | C0011646 | DeepMargin | Dermis |
| Dermal/subcutaneous | DeepMargin | Fat |  |  |
| Dermal subcutaneous | DeepMargin | Fat |  |  |
| Dermal - subcutaneous | DeepMargin | Fat |  |  |
| Dermal-subcutaneous | DeepMargin | Fat |  |  |
| Dermis | CUI | C0011646 | DeepMargin | Dermis |
| Digastric | DeepMargin | Muscle |  |  |
| Digastric triangle | DeepMargin | Muscle |  |  |
| Dilatator naris | DeepMargin | Muscle |  |  |
| Diploe | CUI | C0262950 | DeepMargin | Bone |
| Diploic space | CUI | C0262950 | DeepMargin | Bone |
| ECR | DeepMargin | Muscle |  |  |
| ECRB | DeepMargin | Muscle |  |  |
| ECRL | DeepMargin | Muscle |  |  |
| EDB | DeepMargin | Muscle |  |  |
| EDL | DeepMargin | Muscle |  |  |
| EDM | DeepMargin | Muscle |  |  |
| EHB | DeepMargin | Muscle |  |  |
| EHL | DeepMargin | Muscle |  |  |
| EI | DeepMargin | Muscle |  |  |
| EIP | DeepMargin | Muscle |  |  |
| Elbow | DeepMargin | Bone |  |  |
| EPB | DeepMargin | Muscle |  |  |
| EPL | DeepMargin | Muscle |  |  |
| Erector spinae | DeepMargin | Muscle |  |  |
| Extensor carpi radialis brevis | DeepMargin | Muscle |  |  |
| Extensor carpi radialis longus | DeepMargin | Muscle |  |  |
| Extensor carpi ulnaris | DeepMargin | Muscle |  |  |
| Extensor digiti minimi | DeepMargin | Muscle |  |  |
| Extensor digitorum | DeepMargin | Muscle |  |  |
| Extensor digitorum brevis | DeepMargin | Muscle |  |  |
| Extensor digitorum longus | DeepMargin | Muscle |  |  |
| Extensor hallucis brevis | DeepMargin | Muscle |  |  |
| Extensor hallucis longus | DeepMargin | Muscle |  |  |
| Extensor indicis | DeepMargin | Muscle |  |  |
| Extensor pollicis brevis | DeepMargin | Muscle |  |  |
| Extensor pollicis longus | DeepMargin | Muscle |  |  |
| External oblique | DeepMargin | Muscle |  |  |
| Fascia | CUI | C0015641 | DeepMargin | Fascia |
| Fascial | CUI | C0015641 | DeepMargin | Fascia |
| Fat | CUI | C0222331 | DeepMargin | Fat |
| FCR | DeepMargin | Muscle |  |  |
| FCU | DeepMargin | Muscle |  |  |
| FDP | DeepMargin | Muscle |  |  |
| FDS | DeepMargin | Muscle |  |  |
| Femur | DeepMargin | Bone |  |  |
| Fibula | DeepMargin | Bone |  |  |
| Flexor carpi radialis | DeepMargin | Muscle |  |  |
| Flexor carpi ulnaris | DeepMargin | Muscle |  |  |
| Flexor digiti minimi brevis | DeepMargin | Muscle |  |  |
| Flexor digiti minimi brevis | DeepMargin | Muscle |  |  |
| Flexor digitorum brevis | DeepMargin | Muscle |  |  |
| Flexor digitorum longus | DeepMargin | Muscle |  |  |
| Flexor digitorum profundus | DeepMargin | Muscle |  |  |
| Flexor digitorum superficialis | DeepMargin | Muscle |  |  |
| Flexor hallucis brevis | DeepMargin | Muscle |  |  |
| Flexor hallucis longus | DeepMargin | Muscle |  |  |
| Flexor pollicis brevis | DeepMargin | Muscle |  |  |
| Flexor pollicis longus | DeepMargin | Muscle |  |  |
| FPB | DeepMargin | Muscle |  |  |
| FPL | DeepMargin | Muscle |  |  |
| Galea | CUI | C0225210 | DeepMargin | Galea |
| Galea aponeurotica | CUI | C0225210 | DeepMargin | Galea |
| Galeal | CUI | C0225210 | DeepMargin | Galea |
| Gastrocnemius | DeepMargin | Muscle |  |  |
| Gemelli | DeepMargin | Muscle |  |  |
| Gemellus | DeepMargin | Muscle |  |  |
| Genioglossus | DeepMargin | Muscle |  |  |
| Geniohyoid | DeepMargin | Muscle |  |  |
| Gluteus | DeepMargin | Muscle |  |  |
| Gracilis | DeepMargin | Muscle |  |  |
| Hyoglossus | DeepMargin | Muscle |  |  |
| Hypodermal | DeepMargin | SuperficialFascia |  |  |
| Hypodermis | DeepMargin | SuperficialFascia |  |  |
| Subcutis | DeepMargin | SuperficialFascia |  |  |
| Iliac | DeepMargin | Bone |  |  |
| Iliacus | DeepMargin | Muscle |  |  |
| Iliocostalis | DeepMargin | Muscle |  |  |
| Iliopsoas | DeepMargin | Muscle |  |  |
| Illiac | DeepMargin | Bone |  |  |
| Infraspinatus | DeepMargin | Muscle |  |  |
| Inner table | CUI | C0262950 | DeepMargin | Bone |
| Intercostals | DeepMargin | Muscle |  |  |
| Internal oblique | DeepMargin | Muscle |  |  |
| Interspinales | DeepMargin | Muscle |  |  |
| Intertransversarii | DeepMargin | Muscle |  |  |
| Ischium | DeepMargin | Bone |  |  |
| Latissimus dorsi | DeepMargin | Muscle |  |  |
| Levator anguli oris | DeepMargin | Muscle |  |  |
| Levator ani | DeepMargin | Muscle |  |  |
| Levatores costarum | DeepMargin | Muscle |  |  |
| Levator labii superioris | DeepMargin | Muscle |  |  |
| Levator scapulae | DeepMargin | Muscle |  |  |
| Levator veli palatini | DeepMargin | Muscle |  |  |
| Longissimus | DeepMargin | Muscle |  |  |
| Longissimus capitis | DeepMargin | Muscle |  |  |
| Lumbrical | DeepMargin | Muscle |  |  |
| Lumbricals | DeepMargin | Muscle |  |  |
| Lunate | DeepMargin | Bone |  |  |
| Mandible | DeepMargin | Bone |  |  |
| Manubrium | DeepMargin | Bone |  |  |
| Masseter | DeepMargin | Muscle |  |  |
| Mastoid | DeepMargin | Bone |  |  |
| Medulla | CUI | C0262950 | DeepMargin | Bone |
| Mentalis | DeepMargin | Muscle |  |  |
| Metacarpal | DeepMargin | Bone |  |  |
| Metatarsal | DeepMargin | Bone |  |  |
| Mobile wad | DeepMargin | Muscle |  |  |
| Multifidus | DeepMargin | Muscle |  |  |
| Muscle | CUI | C0026845 | DeepMargin | Muscle |
| Mylohyoid | DeepMargin | Muscle |  |  |
| Nasalis | DeepMargin | Muscle |  |  |
| Navicular | DeepMargin | Bone |  |  |
| Obliquus capitis superior | DeepMargin | Muscle |  |  |
| Obturator externus | DeepMargin | Muscle |  |  |
| Obturator internus | DeepMargin | Muscle |  |  |
| Omohyoid | DeepMargin | Muscle |  |  |
| Opponens digiti minimi | DeepMargin | Muscle |  |  |
| Opponens pollicis | DeepMargin | Muscle |  |  |
| Orbicularis oculi | DeepMargin | Muscle |  |  |
| Orbicularis oris | DeepMargin | Muscle |  |  |
| Outer table | CUI | C0262950 | DeepMargin | Bone |
| Palatoglossus | DeepMargin | Muscle |  |  |
| Palatopharyngeus | DeepMargin | Muscle |  |  |
| Palmar interossei | DeepMargin | Muscle |  |  |
| Palmaris brevis | DeepMargin | Muscle |  |  |
| Palmaris longus | DeepMargin | Muscle |  |  |
| Parotid fascia | DeepMargin | DeepFascia |  |  |
| Parotido masseteric fascia | DeepMargin | DeepFascia |  |  |
| Parotido -masseteric fascia | DeepMargin | DeepFascia |  |  |
| Parotido-masseteric fascia | DeepMargin | DeepFascia |  |  |
| Parotido- masseteric fascia | DeepMargin | DeepFascia |  |  |
| Pectineus | DeepMargin | Muscle |  |  |
| Pectoralis major | DeepMargin | Muscle |  |  |
| Pectoralis minor | DeepMargin | Muscle |  |  |
| Perichondrial | CUI | C0225361 | DeepMargin | Perichondrium |
| Perichondrium | CUI | C0225361 | DeepMargin | Perichondrium |
| Pericranial | CUI | C0031110 | DeepMargin | Periosteum |
| Pericranium | CUI | C0031110 | DeepMargin | Periosteum |
| Periosteal | CUI | C0031110 | DeepMargin | Periosteum |
| Periosteum | CUI | C0031110 | DeepMargin | Periosteum |
| Peroneus brevis | DeepMargin | Muscle |  |  |
| Peroneus longus | DeepMargin | Muscle |  |  |
| Peroneus tertius | DeepMargin | Muscle |  |  |
| Piriformis | DeepMargin | Muscle |  |  |
| Pisiform | DeepMargin | Bone |  |  |
| Plantaris | DeepMargin | Muscle |  |  |
| Platysma | DeepMargin | Muscle |  |  |
| Procerus | DeepMargin | Muscle |  |  |
| Pronator quadratus | DeepMargin | Muscle |  |  |
| Pronator teres | DeepMargin | Muscle |  |  |
| Psoas major | DeepMargin | Muscle |  |  |
| Psoas minor | DeepMargin | Muscle |  |  |
| Pterygoid | DeepMargin | Muscle |  |  |
| Pyramidalis | DeepMargin | Muscle |  |  |
| Quadratus femoris | DeepMargin | Muscle |  |  |
| Quadratus plantae | DeepMargin | Muscle |  |  |
| Quadriceps femoris | DeepMargin | Muscle |  |  |
| Radius | DeepMargin | Bone |  |  |
| Rectus abdominis | DeepMargin | Muscle |  |  |
| Rectus capitis | DeepMargin | Muscle |  |  |
| Rectus capitis lateralis | DeepMargin | Muscle |  |  |
| Rectus femoris | DeepMargin | Muscle |  |  |
| Rhomboid major | DeepMargin | Muscle |  |  |
| Rhomboid minor | DeepMargin | Muscle |  |  |
| Rhomboids | DeepMargin | Muscle |  |  |
| Rib | DeepMargin | Bone |  |  |
| Ribs | DeepMargin | Bone |  |  |
| Risorius | DeepMargin | Muscle |  |  |
| Rotator cuff | DeepMargin | Muscle |  |  |
| Rotatores | DeepMargin | Muscle |  |  |
| Sacrum | DeepMargin | Bone |  |  |
| Salpingopharyngeus | DeepMargin | Muscle |  |  |
| Sartorius | DeepMargin | Muscle |  |  |
| Scalene | DeepMargin | Muscle |  |  |
| Scalenes | DeepMargin | Muscle |  |  |
| Scaphoid | DeepMargin | Bone |  |  |
| Scapula | DeepMargin | Bone |  |  |
| Scarpa's fascia | DeepMargin | SuperficialFascia |  |  |
| Scarpas fascia | DeepMargin | SuperficialFascia |  |  |
| Semimembranosus | DeepMargin | Muscle |  |  |
| Semispinalis | DeepMargin | Muscle |  |  |
| Semispinalis capitis | DeepMargin | Muscle |  |  |
| Semitendinosus | DeepMargin | Muscle |  |  |
| Serratus | DeepMargin | Muscle |  |  |
| Skeletal muscle | CUI | C0026845 | DeepMargin | Muscle |
| SMAS | CUI | C1831580 | DeepMargin | SMAS |
| Smooth muscle | CUI | C0026845 | DeepMargin | Muscle |
| Soleus | DeepMargin | Muscle |  |  |
| Sphenoid | DeepMargin | Bone |  |  |
| Spinalis | DeepMargin | Muscle |  |  |
| Splenius capitis | DeepMargin | Muscle |  |  |
| Splenius capitis | DeepMargin | Muscle |  |  |
| Splenius cervicis | DeepMargin | Muscle |  |  |
| Stapedius | DeepMargin | Muscle |  |  |
| Sternocleidomastoid | DeepMargin | Muscle |  |  |
| Sternohyoid | DeepMargin | Muscle |  |  |
| Sternothyroid | DeepMargin | Muscle |  |  |
| Sternum | DeepMargin | Bone |  |  |
| Striated muscle | CUI | C0026845 | DeepMargin | Muscle |
| Styloglossus | DeepMargin | Muscle |  |  |
| Stylohyoid | DeepMargin | Muscle |  |  |
| Stylopharyngeus | DeepMargin | Muscle |  |  |
| Subclavius | DeepMargin | Muscle |  |  |
| Subcutaneous adipose tissue | CUI | C0222331 | DeepMargin | Fat |
| Subcutaneous fat | CUI | C0222331 | DeepMargin | Fat |
| Subcutaneous tissue | DeepMargin | SuperficialFascia |  |  |
| Subcut fat | CUI | C0222331 | DeepMargin | Fat |
| Subscapularis | DeepMargin | Muscle |  |  |
| Superficial cervical fascia | DeepMargin | SuperficialFascia |  |  |
| Superficial fascia | DeepMargin | SuperficialFascia |  |  |
| Superficial fascial | DeepMargin | SuperficialFascia |  |  |
| Superficial investing layer | DeepMargin | SuperficialFascia |  |  |
| Superficial investing layer cervical fascia | DeepMargin | SuperficialFascia |  |  |
| Superficial investing layer of cervical fascia | DeepMargin | SuperficialFascia |  |  |
| Superficial layer of cervical fascia | DeepMargin | SuperficialFascia |  |  |
| Superficial musculoaponeurotic system | CUI | C1831580 | DeepMargin | SMAS |
| Superficial musculo aponeurotic system | CUI | C1831580 | DeepMargin | SMAS |
| Superficial musculo -aponeurotic system | CUI | C1831580 | DeepMargin | SMAS |
| Superficial musculo - aponeurotic system | CUI | C1831580 | DeepMargin | SMAS |
| Superficial musculo- aponeurotic system | CUI | C1831580 | DeepMargin | SMAS |
| Superficial temporal fascia | DeepMargin | SuperficialFascia |  |  |
| Supinator | DeepMargin | Muscle |  |  |
| Supraspinatus | DeepMargin | Muscle |  |  |
| Talus | DeepMargin | Bone |  |  |
| Temporalis | DeepMargin | Muscle |  |  |
| Temporoparietalis | DeepMargin | Muscle |  |  |
| Tensor | DeepMargin | Muscle |  |  |
| Teres | DeepMargin | Muscle |  |  |
| Thyroarytenoid | DeepMargin | Muscle |  |  |
| Thyrohyoid | DeepMargin | Muscle |  |  |
| Tibia | DeepMargin | Bone |  |  |
| Tibialis | DeepMargin | Muscle |  |  |
| Transversospinales | DeepMargin | Muscle |  |  |
| Transversus abdominis | DeepMargin | Muscle |  |  |
| Transversus thoracis | DeepMargin | Muscle |  |  |
| Trapezium | DeepMargin | Bone |  |  |
| Trapezius | DeepMargin | Muscle |  |  |
| Trapezoid | DeepMargin | Bone |  |  |
| Triceps | DeepMargin | Muscle |  |  |
| Triquetral | DeepMargin | Bone |  |  |
| Triquetrum | DeepMargin | Bone |  |  |
| Trochanter | DeepMargin | Bone |  |  |
| Ulna | DeepMargin | Bone |  |  |
| Vastus | DeepMargin | Muscle |  |  |
| Verticalis | DeepMargin | Muscle |  |  |
| Xiphisternum | DeepMargin | Bone |  |  |
| Zygoma | DeepMargin | Bone |  |  |
| Zygomaticus | DeepMargin | Muscle |  |  |

## *DeepMarginTrigger.lst*

| **Value** |
| --- |
| Down to |
| Excised down to |
| Excised with |
| Excision down to |
| Excision with |
| Included |
| Including |
| Inclusion of |
| Taken down to |
| Taken with |

## *Diagnosis.lst*

| **Value** | **Feature 1** | **Value 1** | **Feature 2** | **Value 2** |
| --- | --- | --- | --- | --- |
| Acquired tufted haemangioma | CUI | C1266161 | CancerType | OtherBenign |
| Acral malignant melanoma | CUI | C0346037 | CancerType | OtherCancerous |
| Acral melanoma | CUI | C0346037 | CancerType | OtherCancerous |
| Actinic keratosis | CUI | C0006079 | CancerType | OtherInSitu |
| Adenoid cystic carcinoma | CUI | C0346017 | CancerType | OtherCancerous |
| Adenoid cystic eccrine carcinoma | CUI | C0346017 | CancerType | OtherCancerous |
| Amelanotic malignant melanoma | CUI | C0206735 | CancerType | OtherCancerous |
| Amelanotic melanoma | CUI | C0206735 | CancerType | OtherCancerous |
| Angiofibroma | CUI | C0206731 | CancerType | OtherBenign |
| Angiofibr oma | CUI | C0206731 | CancerType | OtherBenign |
| Angiofibr -oma | CUI | C0206731 | CancerType | OtherBenign |
| Angiofibr-oma | CUI | C0206731 | CancerType | OtherBenign |
| Angiofibr- oma | CUI | C0206731 | CancerType | OtherBenign |
| Angiokeratoma | CUI | C0002985 | CancerType | OtherBenign |
| Angiosarcoma | CUI | C0018923 | CancerType | OtherCancerous |
| Angiosarcoma | CUI | C0346081 | CancerType | OtherCancerous |
| Apocrine adenocarcinoma | CUI | C0334346 | CancerType | OtherCancerous |
| Apocrine adenoma | CUI | C0334345 | CancerType | OtherBenign |
| Apocrine aden oma | CUI | C0334345 | CancerType | OtherBenign |
| Apocrine aden -oma | CUI | C0334345 | CancerType | OtherBenign |
| Apocrine aden-oma | CUI | C0334345 | CancerType | OtherBenign |
| Apocrine aden- oma | CUI | C0334345 | CancerType | OtherBenign |
| Atypical fibroxanthoma | CUI | C0346053 | CancerType | OtherCancerous |
| Atypical melanocytic naevus | CancerType | OtherBenign |  |  |
| Atypical melanocytic nevus | CancerType | OtherBenign |  |  |
| Balloon cell naevus | CUI | C0334425 | CancerType | OtherBenign |
| Balloon cell naevus | CUI | C0346097 | CancerType | OtherBenign |
| Balloon cell nevus | CUI | C0334425 | CancerType | OtherBenign |
| Balloon cell nevus | CUI | C0346097 | CancerType | OtherBenign |
| Basal/squamous | CUI | C0007117 | CancerType | BasalCellCarcinoma |
| Basal/ squamous | CUI | C0007117 | CancerType | BasalCellCarcinoma |
| Basal/squamous cell carcinoma | CUI | C0007117 | CancerType | BasalCellCarcinoma |
| Basal cell carcinoma | CUI | C0007117 | CancerType | BasalCellCarcinoma |
| Basalsquamous | CUI | C0007117 | CancerType | BasalCellCarcinoma |
| Basal-squamous | CUI | C0007117 | CancerType | BasalCellCarcinoma |
| Basal- squamous | CUI | C0007117 | CancerType | BasalCellCarcinoma |
| Basalsquamous cell carcinoma | CUI | C0007117 | CancerType | BasalCellCarcinoma |
| Basal-squamous cell carcinoma | CUI | C0007117 | CancerType | BasalCellCarcinoma |
| Basal- squamous cell carcinoma | CUI | C0007117 | CancerType | BasalCellCarcinoma |
| Basilloma | CUI | C0007117 | CancerType | BasalCellCarcinoma |
| Basill -oma | CUI | C0007117 | CancerType | BasalCellCarcinoma |
| Basill-oma | CUI | C0007117 | CancerType | BasalCellCarcinoma |
| Basill- oma | CUI | C0007117 | CancerType | BasalCellCarcinoma |
| Basiloma | CUI | C0007117 | CancerType | BasalCellCarcinoma |
| Basil -oma | CUI | C0007117 | CancerType | BasalCellCarcinoma |
| Basil-oma | CUI | C0007117 | CancerType | BasalCellCarcinoma |
| Basil- oma | CUI | C0007117 | CancerType | BasalCellCarcinoma |
| Basisquamous | CUI | C0007117 | CancerType | BasalCellCarcinoma |
| Basi squamous | CUI | C0007117 | CancerType | BasalCellCarcinoma |
| Basi -squamous | CUI | C0007117 | CancerType | BasalCellCarcinoma |
| Basi - squamous | CUI | C0007117 | CancerType | BasalCellCarcinoma |
| Basi-squamous | CUI | C0007117 | CancerType | BasalCellCarcinoma |
| Basi- squamous | CUI | C0007117 | CancerType | BasalCellCarcinoma |
| Basosquamous | CUI | C0007117 | CancerType | BasalCellCarcinoma |
| Baso squamous | CUI | C0007117 | CancerType | BasalCellCarcinoma |
| Baso -squamous | CUI | C0007117 | CancerType | BasalCellCarcinoma |
| Baso - squamous | CUI | C0007117 | CancerType | BasalCellCarcinoma |
| Baso-squamous | CUI | C0007117 | CancerType | BasalCellCarcinoma |
| Baso- squamous | CUI | C0007117 | CancerType | BasalCellCarcinoma |
| Basosquamous cell carcinoma | CUI | C0007117 | CancerType | BasalCellCarcinoma |
| Baso squamous cell carcinoma | CUI | C0007117 | CancerType | BasalCellCarcinoma |
| Baso -squamous cell carcinoma | CUI | C0007117 | CancerType | BasalCellCarcinoma |
| Baso-squamous cell carcinoma | CUI | C0007117 | CancerType | BasalCellCarcinoma |
| Baso- squamous cell carcinoma | CUI | C0007117 | CancerType | BasalCellCarcinoma |
| BCC | CUI | C0007117 | CancerType | BasalCellCarcinoma |
| BCCV | CUI | C0007117 | CancerType | BasalCellCarcinoma |
| BCCX | CUI | C0007117 | CancerType | BasalCellCarcinoma |
| Becker's naevus | CUI | C0263579 | CancerType | OtherBenign |
| Becker's nevus | CUI | C0263579 | CancerType | OtherBenign |
| Becker naevus | CUI | C0263579 | CancerType | OtherBenign |
| Becker nevus | CUI | C0263579 | CancerType | OtherBenign |
| Benign haemangiopericytoma | CUI | C0334541 | CancerType | OtherIntermediate |
| Benign haem angio pericytoma | CUI | C0334541 | CancerType | OtherIntermediate |
| Benign haem angio pericyt oma | CUI | C0334541 | CancerType | OtherIntermediate |
| Benign haem -angio -pericytoma | CUI | C0334541 | CancerType | OtherIntermediate |
| Benign haem -angio -pericyt -oma | CUI | C0334541 | CancerType | OtherIntermediate |
| Benign haem-angio-pericytoma | CUI | C0334541 | CancerType | OtherIntermediate |
| Benign haem-angio-pericyt-oma | CUI | C0334541 | CancerType | OtherIntermediate |
| Benign haem- angio- pericytoma | CUI | C0334541 | CancerType | OtherIntermediate |
| Benign haem- angio- pericyt- oma | CUI | C0334541 | CancerType | OtherIntermediate |
| Benign melanocytic naevus | CUI | C0027962 | CancerType | OtherBenign |
| Benign melanocytic nevus | CUI | C0027962 | CancerType | OtherBenign |
| Blue naevus | CUI | C0206736 | CancerType | OtherBenign |
| Blue nevus | CUI | C0206736 | CancerType | OtherBenign |
| Bowen's disease | CUI | C0006079 | Type | OtherInSitu |
| Cafeaulait patch | CUI | C0221263 | CancerType | OtherBenign |
| Cafe au lait patch | CUI | C0221263 | CancerType | OtherBenign |
| Cafe -au -lait patch | CUI | C0221263 | CancerType | OtherBenign |
| Café -au -lait patch | CUI | C0221263 | CancerType | OtherBenign |
| Café-au-lait patch | CUI | C0221263 | CancerType | OtherBenign |
| Café- au- lait patch | CUI | C0221263 | CancerType | OtherBenign |
| Cafe-au-lait patch | CUI | C0221263 | CancerType | OtherBenign |
| Cafe- au- lait patch | CUI | C0221263 | CancerType | OtherBenign |
| Campbell de Morgan spot | CUI | C0343082 | CancerType | OtherBenign |
| Campbell -de -Morgan spot | CUI | C0343082 | CancerType | OtherBenign |
| Campbell-de-Morgan spot | CUI | C0343082 | CancerType | OtherBenign |
| Campbell- de- Morgan spot | CUI | C0343082 | CancerType | OtherBenign |
| Capillary haemangioma | CUI | C4317089 | CancerType | OtherBenign |
| Capillary haemangi oma | CUI | C4317089 | CancerType | OtherBenign |
| Capillary haemangi -oma | CUI | C4317089 | CancerType | OtherBenign |
| Capillary haemangi-oma | CUI | C4317089 | CancerType | OtherBenign |
| Capillary haemangi- oma | CUI | C4317089 | CancerType | OtherBenign |
| Capillary lymphangioma | CUI | C0334543 | CancerType | OtherCancerous |
| Carcinoma cuniculatum | CUI | C0206706 | CancerType | OtherCancerous |
| Cavernous lymphangioma | CUI | C0346080 | CancerType | OtherCancerous |
| Cellular angiofibroma | CUI | C1367534 | CancerType | OtherBenign |
| Cellular angiofibr oma | CUI | C1367534 | CancerType | OtherBenign |
| Cellular angiofibr -oma | CUI | C1367534 | CancerType | OtherBenign |
| Cellular angiofibr-oma | CUI | C1367534 | CancerType | OtherBenign |
| Cellular angiofibr- oma | CUI | C1367534 | CancerType | OtherBenign |
| Ceruminous adenocarcinoma | CUI | C0334353 | CancerType | OtherCancerous |
| Ceruminous adenoma | CUI | C0334352 | CancerType | OtherBenign |
| Ceruminous aden oma | CUI | C0334352 | CancerType | OtherBenign |
| Ceruminous aden -oma | CUI | C0334352 | CancerType | OtherBenign |
| Ceruminous aden-oma | CUI | C0334352 | CancerType | OtherBenign |
| Ceruminous aden- oma | CUI | C0334352 | CancerType | OtherBenign |
| Choristoma | CUI | C0011649 | CancerType | OtherBenign |
| Chorist oma | CUI | C0011649 | CancerType | OtherBenign |
| Chorist -oma | CUI | C0011649 | CancerType | OtherBenign |
| Chorist-oma | CUI | C0011649 | CancerType | OtherBenign |
| Chorist- oma | CUI | C0011649 | CancerType | OtherBenign |
| Clear cell acanthoma | CUI | C0333992 | CancerType | OtherBenign |
| Clear cell hidradenoma | CUI | C1370701 | CancerType | OtherBenign |
| Clear cell hidraden oma | CUI | C1370701 | CancerType | OtherBenign |
| Clear cell hidraden -oma | CUI | C1370701 | CancerType | OtherBenign |
| Clear cell hidraden-oma | CUI | C1370701 | CancerType | OtherBenign |
| Clear cell hidraden- oma | CUI | C1370701 | CancerType | OtherBenign |
| CMN | CUI | C1318558 | CancerType | OtherBenign |
| Common wart | CUI | C0043037 | CancerType | OtherBenign |
| Composite haemangioendothelioma | CUI | C1304513 | CancerType | OtherIntermediate |
| Composite haem angio endothelioma | CUI | C1304513 | CancerType | OtherIntermediate |
| Composite haem angio endotheli oma | CUI | C1304513 | CancerType | OtherIntermediate |
| Composite haem -angio -endothelioma | CUI | C1304513 | CancerType | OtherIntermediate |
| Composite haem -angio -endotheli -oma | CUI | C1304513 | CancerType | OtherIntermediate |
| Composite haem-angio-endothelioma | CUI | C1304513 | CancerType | OtherIntermediate |
| Composite haem-angio-endotheli-oma | CUI | C1304513 | CancerType | OtherIntermediate |
| Composite haem- angio- endothelioma | CUI | C1304513 | CancerType | OtherIntermediate |
| Composite haem- angio- endotheli- oma | CUI | C1304513 | CancerType | OtherIntermediate |
| Compound naevus | CUI | C0259781 | CancerType | OtherBenign |
| Compound nevus | CUI | C0259781 | CancerType | OtherBenign |
| Congenital haemangioma | CUI | C0235753 | CancerType | OtherBenign |
| Congenital haemangi oma | CUI | C0235753 | CancerType | OtherBenign |
| Congenital haemangi -oma | CUI | C0235753 | CancerType | OtherBenign |
| Congenital haemangi-oma | CUI | C0235753 | CancerType | OtherBenign |
| Congenital haemangi- oma | CUI | C0235753 | CancerType | OtherBenign |
| Congenital melanocytic naevus | CUI | C1318558 | CancerType | OtherBenign |
| Congenital melanocytic nevus | CUI | C1318558 | CancerType | OtherBenign |
| Cutaneous adenoid cystic carcinoma | CUI | C0346017 | CancerType | OtherCancerous |
| Cutaneous fibrous histiocytoma | CUI | C0002991 | CancerType | OtherBenign |
| Cutaneous fibrous histiocyt oma | CUI | C0002991 | CancerType | OtherBenign |
| Cutaneous fibrous histiocyt -oma | CUI | C0002991 | CancerType | OtherBenign |
| Cutaneous fibrous histiocyt-oma | CUI | C0002991 | CancerType | OtherBenign |
| Cutaneous fibrous histiocyt- oma | CUI | C0002991 | CancerType | OtherBenign |
| Cutaneous horn | CUI | C0085664 | CancerType | OtherBenign |
| Cutaneous leiomyosarcoma | CUI | C0346067 | CancerType | OtherCancerous |
| Cutaneous lymphoma | CUI | C1276146 | CancerType | OtherCancerous |
| Cutaneous mucinous carcinoma | CUI | C1879641 | CancerType | OtherCancerous |
| Cylindroma | CUI | C1305968 | CancerType | OtherBenign |
| Cylindr oma | CUI | C1305968 | CancerType | OtherBenign |
| Cylindr -oma | CUI | C1305968 | CancerType | OtherBenign |
| Cylindr-oma | CUI | C1305968 | CancerType | OtherBenign |
| Cylindr- oma | CUI | C1305968 | CancerType | OtherBenign |
| Cystic hygroma | CUI | C0206620 | CancerType | OtherCancerous |
| Cystic lymphangioma | CUI | C0206620 | CancerType | OtherCancerous |
| Cystic trichoblastoma | CUI | C1299879 | CancerType | OtherBenign |
| Cystic tricho blastoma | CUI | C1299879 | CancerType | OtherBenign |
| Cystic tricho -blastoma | CUI | C1299879 | CancerType | OtherBenign |
| Cystic tricho-blastoma | CUI | C1299879 | CancerType | OtherBenign |
| Cystic tricho- blastoma | CUI | C1299879 | CancerType | OtherBenign |
| Dabska tumour | CUI | C0346087 | CancerType | OtherIntermediate |
| Dermatofibroma | CUI | C0002991 | CancerType | OtherBenign |
| Dermatofibr oma | CUI | C0002991 | CancerType | OtherBenign |
| Dermatofibr -oma | CUI | C0002991 | CancerType | OtherBenign |
| Dermatofibr-oma | CUI | C0002991 | CancerType | OtherBenign |
| Dermatofibr- oma | CUI | C0002991 | CancerType | OtherBenign |
| Dermatofibrosarcoma protuberans | CUI | C0392784 | CancerType | OtherCancerous |
| Dermoid cyst | CUI | C0011649 | CancerType | OtherBenign |
| Desmoplastic malignant melanoma | CUI | C0334439 | CancerType | OtherCancerous |
| Desmoplastic melanoma | CUI | C0334439 | CancerType | OtherCancerous |
| Diffuse melanocytosis | CUI | C1266112 | CancerType | OtherBenign |
| Digital mucous cyst | CUI | C1275289 | CancerType | OtherBenign |
| Digital myxoid pseudocyst | CUI | C1275289 | CancerType | OtherBenign |
| Digital papillary adenocarcinoma | CUI | C1367789 | CancerType | OtherCancerous |
| Digital papillary eccrine carcinoma | CUI | C1367789 | CancerType | OtherCancerous |
| Dysplastic naevus | CUI | C0205748 | CancerType | OtherBenign |
| Dysplastic nevus | CUI | C0205748 | CancerType | OtherBenign |
| ECC | CUI | C0007117 | CancerType | BasalCellCarcinoma |
| Eccrine acrospiroma | CUI | C0206671 | CancerType | OtherBenign |
| Eccrine acro spiroma | CUI | C0206671 | CancerType | OtherBenign |
| Eccrine acro spir oma | CUI | C0206671 | CancerType | OtherBenign |
| Eccrine acro -spiroma | CUI | C0206671 | CancerType | OtherBenign |
| Eccrine acro -spir -oma | CUI | C0206671 | CancerType | OtherBenign |
| Eccrine acro-spiroma | CUI | C0206671 | CancerType | OtherBenign |
| Eccrine acro-spir-oma | CUI | C0206671 | CancerType | OtherBenign |
| Eccrine acro- spiroma | CUI | C0206671 | CancerType | OtherBenign |
| Eccrine acro- spir- oma | CUI | C0206671 | CancerType | OtherBenign |
| Eccrine adenocarcinoma | CUI | C1266066 | CancerType | OtherCancerous |
| Eccrine dermal cylindroma | CUI | C1305968 | CancerType | OtherBenign |
| Eccrine dermal cylindr oma | CUI | C1305968 | CancerType | OtherBenign |
| Eccrine dermal cylindr -oma | CUI | C1305968 | CancerType | OtherBenign |
| Eccrine dermal cylindr-oma | CUI | C1305968 | CancerType | OtherBenign |
| Eccrine dermal cylindr- oma | CUI | C1305968 | CancerType | OtherBenign |
| Eccrine papillary adenocarcinoma | CUI | C2211427 | CancerType | OtherCancerous |
| Eccrine papillary adenoma | CUI | C0334350 | CancerType | OtherBenign |
| Eccrine porocarcinoma | CUI | C1266065 | CancerType | OtherCancerous |
| Eccrine poroma | CUI | C1533161 | CancerType | OtherBenign |
| Eccrine spiradenoma | CUI | C0334347 | CancerType | OtherBenign |
| Eccrine spiraden oma | CUI | C0334347 | CancerType | OtherBenign |
| Eccrine spiraden -oma | CUI | C0334347 | CancerType | OtherBenign |
| Eccrine spiraden-oma | CUI | C0334347 | CancerType | OtherBenign |
| Eccrine spiraden- oma | CUI | C0334347 | CancerType | OtherBenign |
| Eccrine spira denoma | CUI | C0334347 | CancerType | OtherBenign |
| Eccrine spira -denoma | CUI | C0334347 | CancerType | OtherBenign |
| Eccrine spira-denoma | CUI | C0334347 | CancerType | OtherBenign |
| Eccrine spira- denoma | CUI | C0334347 | CancerType | OtherBenign |
| Endovascular papillary angioendothelioma | CUI | C0346087 | CancerType | OtherIntermediate |
| Endovascular papillary angio -endothelioma | CUI | C0346087 | CancerType | OtherIntermediate |
| Endovascular papillary angio-endothelioma | CUI | C0346087 | CancerType | OtherIntermediate |
| Endovascular papillary angio- endothelioma | CUI | C0346087 | CancerType | OtherIntermediate |
| Endo vascular papillary angioendothelioma | CUI | C0346087 | CancerType | OtherIntermediate |
| Endo -vascular papillary angioendothelioma | CUI | C0346087 | CancerType | OtherIntermediate |
| Endo -vascular papillary angio endothelioma | CUI | C0346087 | CancerType | OtherIntermediate |
| Endo-vascular papillary angioendothelioma | CUI | C0346087 | CancerType | OtherIntermediate |
| Endo-vascular papillary angio endothelioma | CUI | C0346087 | CancerType | OtherIntermediate |
| Endo- vascular papillary angioendothelioma | CUI | C0346087 | CancerType | OtherIntermediate |
| Endo- vascular papillary angio endothelioma | CUI | C0346087 | CancerType | OtherIntermediate |
| Ephelis | CUI | C1055787 | CancerType | OtherBenign |
| Epidermoid cyst | CUI | C0014511 | CancerType | OtherBenign |
| Epithelial cyst | CUI | C0014511 | CancerType | OtherBenign |
| Epithelial inclusion cyst | CUI | C0259770 | CancerType | OtherBenign |
| Epithelioid and spindle cell naevus | CUI | C0206739 | CancerType | OtherBenign |
| Epithelioid and spindle cell nevus | CUI | C0206739 | CancerType | OtherBenign |
| Epithelioid cell nevus | CUI | C0259820 | CancerType | OtherBenign |
| Epithelioid haemangioendothelioma | CUI | C0206732 | CancerType | OtherCancerous |
| Epithelioid haemangioma | CUI | C0002989 | CancerType | OtherBenign |
| Epithelioid haemangi oma | CUI | C0002989 | CancerType | OtherBenign |
| Epithelioid haemangi -oma | CUI | C0002989 | CancerType | OtherBenign |
| Epithelioid haemangi-oma | CUI | C0002989 | CancerType | OtherBenign |
| Epithelioid haemangi- oma | CUI | C0002989 | CancerType | OtherBenign |
| Epithelioid sarcoma -like hemangioendothelioma | CUI | C3840252 | CancerType | OtherIntermediate |
| Epithelioid sarcoma-like hemangioendothelioma | CUI | C3840252 | CancerType | OtherIntermediate |
| Epithelioid sarcoma- like hemangioendothelioma | CUI | C3840252 | CancerType | OtherIntermediate |
| Extramammary Paget disease | CUI | C0346032 | CancerType | OtherInSitu |
| Fibroepithelial polyp | CUI | C0037293 | CancerType | OtherBenign |
| Fibrous histiocytoma | CUI | C0002991 | CancerType | OtherBenign |
| Fibrous histiocyt oma | CUI | C0002991 | CancerType | OtherBenign |
| Fibrous histiocyt -oma | CUI | C0002991 | CancerType | OtherBenign |
| Fibrous histiocyt-oma | CUI | C0002991 | CancerType | OtherBenign |
| Fibrous histiocyt- oma | CUI | C0002991 | CancerType | OtherBenign |
| Follicular fibroma | CUI | C0346011 | CancerType | OtherBenign |
| Follicular fibr oma | CUI | C0346011 | CancerType | OtherBenign |
| Follicular fibr -oma | CUI | C0346011 | CancerType | OtherBenign |
| Follicular fibr-oma | CUI | C0346011 | CancerType | OtherBenign |
| Follicular fibr- oma | CUI | C0346011 | CancerType | OtherBenign |
| Freckle | CUI | C1055787 | CancerType | OtherBenign |
| Giant cell angiofibroma | CUI | C1367539 | CancerType | OtherBenign |
| Giant cell angiofibr oma | CUI | C1367539 | CancerType | OtherBenign |
| Giant cell angiofibr -oma | CUI | C1367539 | CancerType | OtherBenign |
| Giant cell angiofibr-oma | CUI | C1367539 | CancerType | OtherBenign |
| Giant cell angiofibr- oma | CUI | C1367539 | CancerType | OtherBenign |
| Giant congenital melanocytic naevus | CUI | C1318558 | CancerType | OtherBenign |
| Giant congenital melanocytic nevus | CUI | C1318558 | CancerType | OtherBenign |
| Glomus tumour | CUI | C0017653 | CancerType | OtherBenign |
| Haemangioblastoma | CUI | C0206734 | CancerType | OtherIntermediate |
| Haem angio blastoma | CUI | C0206734 | CancerType | OtherIntermediate |
| Haem angio blast oma | CUI | C0206734 | CancerType | OtherIntermediate |
| Haem -angio -blastoma | CUI | C0206734 | CancerType | OtherIntermediate |
| Haem -angio -blast -oma | CUI | C0206734 | CancerType | OtherIntermediate |
| Haem-angio-blastoma | CUI | C0206734 | CancerType | OtherIntermediate |
| Haem-angio-blast-oma | CUI | C0206734 | CancerType | OtherIntermediate |
| Haem- angio- blastoma | CUI | C0206734 | CancerType | OtherIntermediate |
| Haem- angio- blast- oma | CUI | C0206734 | CancerType | OtherIntermediate |
| Haemangioendothelial sarcoma | CUI | C0018915 | CancerType | OtherCancerous |
| Haemangioendothelioma | CUI | C0018915 | CancerType | OtherBenign |
| Haemangioendothelioma | CUI | C0018915 | CancerType | OtherCancerous |
| Haemangioendothelioma | CUI | C0018915 | CancerType | OtherIntermediate |
| Haemangioendotheli oma | CUI | C0018915 | CancerType | OtherBenign |
| Haemangioendotheli -oma | CUI | C0018915 | CancerType | OtherBenign |
| Haemangioendotheli-oma | CUI | C0018915 | CancerType | OtherBenign |
| Haemangioendotheli- oma | CUI | C0018915 | CancerType | OtherBenign |
| Haem angioendothelioma | CUI | C0018915 | CancerType | OtherBenign |
| Haem angio endo thelioma | CUI | C0018915 | CancerType | OtherIntermediate |
| Haem angio endo theli oma | CUI | C0018915 | CancerType | OtherIntermediate |
| Haem -angioendotheli oma | CUI | C0018915 | CancerType | OtherBenign |
| Haem -angioendotheli -oma | CUI | C0018915 | CancerType | OtherBenign |
| Haem -angio -endothelioma | CUI | C0018915 | CancerType | OtherBenign |
| Haem -angio -endotheli -oma | CUI | C0018915 | CancerType | OtherBenign |
| Haem -angio -endo -thelioma | CUI | C0018915 | CancerType | OtherIntermediate |
| Haem -angio -endo -theli -oma | CUI | C0018915 | CancerType | OtherIntermediate |
| Haem-angioendotheli oma | CUI | C0018915 | CancerType | OtherBenign |
| Haem-angioendotheli-oma | CUI | C0018915 | CancerType | OtherBenign |
| Haem-angio-endothelioma | CUI | C0018915 | CancerType | OtherBenign |
| Haem-angio-endotheli-oma | CUI | C0018915 | CancerType | OtherBenign |
| Haem-angio-endo-thelioma | CUI | C0018915 | CancerType | OtherIntermediate |
| Haem-angio-endo-theli-oma | CUI | C0018915 | CancerType | OtherIntermediate |
| Haem- angioendotheli oma | CUI | C0018915 | CancerType | OtherBenign |
| Haem- angioendotheli- oma | CUI | C0018915 | CancerType | OtherBenign |
| Haem- angio- endothelioma | CUI | C0018915 | CancerType | OtherBenign |
| Haem- angio- endotheli- oma | CUI | C0018915 | CancerType | OtherBenign |
| Haem- angio- endo- thelioma | CUI | C0018915 | CancerType | OtherIntermediate |
| Haem- angio- endo- theli- oma | CUI | C0018915 | CancerType | OtherIntermediate |
| Haemangioma | CUI | C0018916 | CancerType | OtherBenign |
| Haemangi oma | CUI | C0018916 | CancerType | OtherBenign |
| Haemangi -oma | CUI | C0018916 | CancerType | OtherBenign |
| Haemangi-oma | CUI | C0018916 | CancerType | OtherBenign |
| Haemangi- oma | CUI | C0018916 | CancerType | OtherBenign |
| Haem -angioma | CUI | C0018916 | CancerType | OtherBenign |
| Haem-angioma | CUI | C0018916 | CancerType | OtherBenign |
| Haem- angioma | CUI | C0018916 | CancerType | OtherBenign |
| Haemangioma simplex | CUI | C4317089 | CancerType | OtherBenign |
| Haemangi oma simplex | CUI | C4317089 | CancerType | OtherBenign |
| Haemangi -oma simplex | CUI | C4317089 | CancerType | OtherBenign |
| Haemangi-oma simplex | CUI | C4317089 | CancerType | OtherBenign |
| Haemangi- oma simplex | CUI | C4317089 | CancerType | OtherBenign |
| Haemangiopericytoma | CUI | C0018922 | CancerType | OtherBenign |
| Haemangiopericyt oma | CUI | C0018922 | CancerType | OtherBenign |
| Haemangiopericyt -oma | CUI | C0018922 | CancerType | OtherBenign |
| Haemangiopericyt-oma | CUI | C0018922 | CancerType | OtherBenign |
| Haemangiopericyt- oma | CUI | C0018922 | CancerType | OtherBenign |
| Haem angiopericytoma | CUI | C0018922 | CancerType | OtherBenign |
| Haem -angiopericyt oma | CUI | C0018922 | CancerType | OtherBenign |
| Haem -angiopericyt -oma | CUI | C0018922 | CancerType | OtherBenign |
| Haem -angio -pericytoma | CUI | C0018922 | CancerType | OtherBenign |
| Haem -angio -pericyt -oma | CUI | C0018922 | CancerType | OtherBenign |
| Haem-angiopericyt oma | CUI | C0018922 | CancerType | OtherBenign |
| Haem-angiopericyt-oma | CUI | C0018922 | CancerType | OtherBenign |
| Haem-angio-pericytoma | CUI | C0018922 | CancerType | OtherBenign |
| Haem-angio-pericyt-oma | CUI | C0018922 | CancerType | OtherBenign |
| Haem- angiopericyt oma | CUI | C0018922 | CancerType | OtherBenign |
| Haem- angiopericyt- oma | CUI | C0018922 | CancerType | OtherBenign |
| Haem- angio- pericytoma | CUI | C0018922 | CancerType | OtherBenign |
| Haem- angio- pericyt- oma | CUI | C0018922 | CancerType | OtherBenign |
| Haemangiosarcoma | CUI | C0018923 | CancerType | OtherCancerous |
| Haemolymphangioma | CUI | C0334544 | CancerType | OtherCancerous |
| Hairy naevus | CUI | C0018508 | CancerType | OtherBenign |
| Hairy nevus | CUI | C0018508 | CancerType | OtherBenign |
| Halo naevus | CUI | C0474824 | CancerType | OtherBenign |
| Halo nevus | CUI | C0474824 | CancerType | OtherBenign |
| Hidradenoma papilliferum | CUI | C0334348 | CancerType | OtherBenign |
| Hidrocystoma | CUI | C0206672 | CancerType | OtherBenign |
| Hidrocyst oma | CUI | C0206672 | CancerType | OtherBenign |
| Hidro cystoma | CUI | C0206672 | CancerType | OtherBenign |
| Hidro cyst oma | CUI | C0206672 | CancerType | OtherBenign |
| Hidro cyst -oma | CUI | C0206672 | CancerType | OtherBenign |
| Hidro cyst-oma | CUI | C0206672 | CancerType | OtherBenign |
| Hidro cyst- oma | CUI | C0206672 | CancerType | OtherBenign |
| Hidro -cystoma | CUI | C0206672 | CancerType | OtherBenign |
| Hidro-cystoma | CUI | C0206672 | CancerType | OtherBenign |
| Hidro- cystoma | CUI | C0206672 | CancerType | OtherBenign |
| Histiocytoid haemangioma | CUI | C0205788 | CancerType | OtherBenign |
| Histiocytoid haemangi oma | CUI | C0205788 | CancerType | OtherBenign |
| Histiocytoid haemangi -oma | CUI | C0205788 | CancerType | OtherBenign |
| Histiocytoid haemangi-oma | CUI | C0205788 | CancerType | OtherBenign |
| Histiocytoid haemangi- oma | CUI | C0205788 | CancerType | OtherBenign |
| Hutchinson's melanotic freckle | CUI | C0149722 | CancerType | OtherInSitu |
| Inclusion cyst | CUI | C0259770 | CancerType | OtherBenign |
| Infantile haemangioma | CUI | C4317089 | CancerType | OtherBenign |
| Infantile haemangi oma | CUI | C4317089 | CancerType | OtherBenign |
| Infantile haemangi -oma | CUI | C4317089 | CancerType | OtherBenign |
| Infantile haemangi-oma | CUI | C4317089 | CancerType | OtherBenign |
| Infantile haemangi- oma | CUI | C4317089 | CancerType | OtherBenign |
| In situ malignant melanoma | CUI | C0854696 | CancerType | OtherInSitu |
| In -situ malignant melanoma | CUI | C0854696 | CancerType | OtherInSitu |
| In-situ malignant melanoma | CUI | C0854696 | CancerType | OtherInSitu |
| In- situ malignant melanoma | CUI | C0854696 | CancerType | OtherInSitu |
| In situ melanoma | CUI | C0854696 | CancerType | OtherInSitu |
| In -situ melanoma | CUI | C0854696 | CancerType | OtherInSitu |
| In-situ melanoma | CUI | C0854696 | CancerType | OtherInSitu |
| In- situ melanoma | CUI | C0854696 | CancerType | OtherInSitu |
| Intimal sarcoma | CUI | C1708550 | CancerType | OtherCancerous |
| Intradermal naevus | CUI | C0206737 | CancerType | OtherBenign |
| Intra dermal naevus | CUI | C0206737 | CancerType | OtherBenign |
| Intra -dermal naevus | CUI | C0206737 | CancerType | OtherBenign |
| Intra-dermal naevus | CUI | C0206737 | CancerType | OtherBenign |
| Intra- dermal naevus | CUI | C0206737 | CancerType | OtherBenign |
| Intradermal nevus | CUI | C0206737 | CancerType | OtherBenign |
| Intra dermal nevus | CUI | C0206737 | CancerType | OtherBenign |
| Intra -dermal nevus | CUI | C0206737 | CancerType | OtherBenign |
| Intra-dermal nevus | CUI | C0206737 | CancerType | OtherBenign |
| Intra- dermal nevus | CUI | C0206737 | CancerType | OtherBenign |
| Intramuscular haemangioma | CUI | C0205789 | CancerType | OtherBenign |
| Intra muscular haemangioma | CUI | C0205789 | CancerType | OtherBenign |
| Intra muscular haem angioma | CUI | C0205789 | CancerType | OtherBenign |
| Intra muscular haem angi oma | CUI | C0205789 | CancerType | OtherBenign |
| Intra muscular haem angi -oma | CUI | C0205789 | CancerType | OtherBenign |
| Intra muscular haem angi-oma | CUI | C0205789 | CancerType | OtherBenign |
| Intra muscular haem angi- oma | CUI | C0205789 | CancerType | OtherBenign |
| Intra -muscular haemangioma | CUI | C0205789 | CancerType | OtherBenign |
| Intra -muscular haem angioma | CUI | C0205789 | CancerType | OtherBenign |
| Intra -muscular haem angi oma | CUI | C0205789 | CancerType | OtherBenign |
| Intra -muscular haem angi -oma | CUI | C0205789 | CancerType | OtherBenign |
| Intra-muscular haemangioma | CUI | C0205789 | CancerType | OtherBenign |
| Intra-muscular haem angioma | CUI | C0205789 | CancerType | OtherBenign |
| Intra-muscular haem angi oma | CUI | C0205789 | CancerType | OtherBenign |
| Intra-muscular haem angi-oma | CUI | C0205789 | CancerType | OtherBenign |
| Intra- muscular haemangioma | CUI | C0205789 | CancerType | OtherBenign |
| Intra- muscular haem angioma | CUI | C0205789 | CancerType | OtherBenign |
| Intra- muscular haem angi oma | CUI | C0205789 | CancerType | OtherBenign |
| Intra- muscular haem angi- oma | CUI | C0205789 | CancerType | OtherBenign |
| Junctional naevus | CUI | C0334433 | CancerType | OtherBenign |
| Junctional nevus | CUI | C0334433 | CancerType | OtherBenign |
| Juvenile haemangioma | CUI | C4317089 | CancerType | OtherBenign |
| Juvenile haemangi oma | CUI | C4317089 | CancerType | OtherBenign |
| Juvenile haemangi -oma | CUI | C4317089 | CancerType | OtherBenign |
| Juvenile haemangi-oma | CUI | C4317089 | CancerType | OtherBenign |
| Juvenile haemangi- oma | CUI | C4317089 | CancerType | OtherBenign |
| Kaposiform haemangioendothelioma | CUI | C1367420 | CancerType | OtherBenign |
| Kaposiform haemangioendothelioma | CUI | C1367420 | CancerType | OtherIntermediate |
| Kaposiform haem angioendothelioma | CUI | C1367420 | CancerType | OtherIntermediate |
| Kaposiform haem angio endothelioma | CUI | C1367420 | CancerType | OtherIntermediate |
| Kaposiform haem -angioendothelioma | CUI | C1367420 | CancerType | OtherIntermediate |
| Kaposiform haem -angio -endothelioma | CUI | C1367420 | CancerType | OtherIntermediate |
| Kaposiform haem-angioendothelioma | CUI | C1367420 | CancerType | OtherIntermediate |
| Kaposiform haem-angio-endothelioma | CUI | C1367420 | CancerType | OtherIntermediate |
| Kaposiform haem- angioendothelioma | CUI | C1367420 | CancerType | OtherIntermediate |
| Kaposiform haem- angio- endothelioma | CUI | C1367420 | CancerType | OtherIntermediate |
| Kaposi sarcoma | CUI | C0153560 | CancerType | OtherCancerous |
| Keratin horn | CUI | C0085664 | CancerType | OtherBenign |
| Keratoacanthoma | CUI | C0022572 | CancerType | OtherBenign |
| Kerato acanthoma | CUI | C0022572 | CancerType | OtherBenign |
| Kerato -acanthoma | CUI | C0022572 | CancerType | OtherBenign |
| Kerato-acanthoma | CUI | C0022572 | CancerType | OtherBenign |
| Kerato- acanthoma | CUI | C0022572 | CancerType | OtherBenign |
| Kupffer cell sarcoma | CUI | C0334534 | CancerType | OtherCancerous |
| Lentigo maligna | CUI | C0149722 | CancerType | OtherInSitu |
| Lentigo malignan melanoma | CUI | C2739810 | CancerType | OtherCancerous |
| Lentigo melanoma | CUI | C2739810 | CancerType | OtherCancerous |
| Lentigo senile | CUI | C0036651 | CancerType | OtherBenign |
| Lentigo senilis | CUI | C0036651 | CancerType | OtherBenign |
| Lentigo simplex | CUI | C0302255 | CancerType | OtherBenign |
| Lobular capillary haemangioma | CUI | C0085653 | CancerType | OtherBenign |
| Lobular capillary haemangi oma | CUI | C0085653 | CancerType | OtherBenign |
| Lobular capillary haemangi -oma | CUI | C0085653 | CancerType | OtherBenign |
| Lobular capillary haemangi-oma | CUI | C0085653 | CancerType | OtherBenign |
| Lobular capillary haemangi- oma | CUI | C0085653 | CancerType | OtherBenign |
| Lymphangioendothelial sarcoma | CUI | C0346082 | CancerType | OtherCancerous |
| Lymphangioendothelioma | CUI | C0024217 | CancerType | OtherCancerous |
| Lymphangioma | CUI | C0024221 | CancerType | OtherCancerous |
| Lymphangiomyoma | CUI | C0024223 | CancerType | OtherCancerous |
| Lymphangiomyomatosis | CUI | C0751674 | CancerType | OtherIntermediate |
| Lymph angio myo matosis | CUI | C0751674 | CancerType | OtherIntermediate |
| Lymph -angio -myo -matosis | CUI | C0751674 | CancerType | OtherIntermediate |
| Lymph-angio-myo-matosis | CUI | C0751674 | CancerType | OtherIntermediate |
| Lymph- angio- myo- matosis | CUI | C0751674 | CancerType | OtherIntermediate |
| Lymphangiosarcoma | CUI | C0346082 | CancerType | OtherCancerous |
| Malignant desmoplastic melanoma | CUI | C0334439 | CancerType | OtherCancerous |
| Malignant eccrine spiradenoma | CUI | C1266063 | CancerType | OtherCancerous |
| Malignant haemangiopericytoma | CUI | C0334542 | CancerType | OtherCancerous |
| Malignant lymphangiosarcoma | CUI | C0346082 | CancerType | OtherCancerous |
| Malignant melanoma | CUI | C0151779 | CancerType | OtherCancerous |
| Malignant nodular hidradenoma | CUI | C2211426 | CancerType | OtherCancerous |
| Malignant syringoma | CUI | C0346027 | CancerType | OtherCancerous |
| Melanocytic naevus | CUI | C0027962 | CancerType | OtherBenign |
| Melanocytic nevus | CUI | C0027962 | CancerType | OtherBenign |
| Melanoma | CUI | C0151779 | CancerType | OtherCancerous |
| Melanoma in situ | CUI | C0854696 | CancerType | OtherInSitu |
| Merkel cell carcinoma | CUI | C0007129 | CancerType | OtherCancerous |
| Milia | CUI | C0345996 | CancerType | OtherBenign |
| Milium cyst | CUI | C0345996 | CancerType | OtherBenign |
| Mongolian blue spot | CUI | C0265985 | CancerType | OtherBenign |
| Mucinous carcinoma | CUI | C1879641 | CancerType | OtherCancerous |
| Myointimoma | CancerType | OtherBenign |  |  |
| Myointim oma | CancerType | OtherBenign |  |  |
| Myointim -oma | CancerType | OtherBenign |  |  |
| Myointim-oma | CancerType | OtherBenign |  |  |
| Myointim- oma | CancerType | OtherBenign |  |  |
| Naevus of Ito | CancerType | OtherBenign |  |  |
| Naevus of Ota | CUI | C0027961 | CancerType | OtherBenign |
| Neurilemmoma | CUI | C0027809 | CancerType | OtherBenign |
| Neurofibroma | CUI | C0027830 | CancerType | OtherBenign |
| Neuronaevus | CUI | C0334430 | CancerType | OtherBenign |
| Neuronevus | CUI | C0334430 | CancerType | OtherBenign |
| nevus of Ito | CancerType | OtherBenign |  |  |
| nevus of Ota | CUI | C0027961 | CancerType | OtherBenign |
| Nodular hidradenoma | CUI | C4505470 | CancerType | OtherBenign |
| Nodular hidraden oma | CUI | C4505470 | CancerType | OtherBenign |
| Nodular hidraden -oma | CUI | C4505470 | CancerType | OtherBenign |
| Nodular hidraden-oma | CUI | C4505470 | CancerType | OtherBenign |
| Nodular hidraden- oma | CUI | C4505470 | CancerType | OtherBenign |
| Nodular malignant melanoma | CUI | C0334424 | CancerType | OtherCancerous |
| Nodular melanoma | CUI | C0334424 | CancerType | OtherCancerous |
| Nonpigmented naevus | CUI | C0334432 | CancerType | OtherBenign |
| Nonpigmented nevus | CUI | C0334432 | CancerType | OtherBenign |
| Nonspecific | CancerType | Non -specific |  |  |
| Nonspecific | CancerType | Non- specific |  |  |
| Nonspecific | CancerType | Non-specific |  |  |
| Non specific | CancerType | Non -specific |  |  |
| Non specific | CancerType | Non- specific |  |  |
| Non specific | CancerType | Non-specific |  |  |
| Non -specific | CancerType | Non -specific |  |  |
| Non-specific | CancerType | Non-specific |  |  |
| Non- specific | CancerType | Non- specific |  |  |
| Papillary hidradenoma | CUI | C0334348 | CancerType | OtherBenign |
| Papillary intralymphatic angioendomethelioma | CUI | C0346087 | CancerType | OtherIntermediate |
| Papillary intralymphatic angio -endomethelioma | CUI | C0346087 | CancerType | OtherIntermediate |
| Papillary intralymphatic angio -endo -methelioma | CUI | C0346087 | CancerType | OtherIntermediate |
| Papillary intralymphatic angio-endomethelioma | CUI | C0346087 | CancerType | OtherIntermediate |
| Papillary intralymphatic angio-endo-methelioma | CUI | C0346087 | CancerType | OtherIntermediate |
| Papillary intralymphatic angio- endomethelioma | CUI | C0346087 | CancerType | OtherIntermediate |
| Papillary intralymphatic angio- endo- methelioma | CUI | C0346087 | CancerType | OtherIntermediate |
| Papillary intra lymphatic angioendomethelioma | CUI | C0346087 | CancerType | OtherIntermediate |
| Papillary intra -lymphatic angioendomethelioma | CUI | C0346087 | CancerType | OtherIntermediate |
| Papillary intra -lymphatic angio endomethelioma | CUI | C0346087 | CancerType | OtherIntermediate |
| Papillary intra -lymphatic angio endo methelioma | CUI | C0346087 | CancerType | OtherIntermediate |
| Papillary intra-lymphatic angioendomethelioma | CUI | C0346087 | CancerType | OtherIntermediate |
| Papillary intra-lymphatic angio endomethelioma | CUI | C0346087 | CancerType | OtherIntermediate |
| Papillary intra-lymphatic angio endo methelioma | CUI | C0346087 | CancerType | OtherIntermediate |
| Papillary intra- lymphatic angioendomethelioma | CUI | C0346087 | CancerType | OtherIntermediate |
| Papillary intra- lymphatic angio endomethelioma | CUI | C0346087 | CancerType | OtherIntermediate |
| Papillary intra- lymphatic angio endo methelioma | CUI | C0346087 | CancerType | OtherIntermediate |
| Papillary syringadenoma | CUI | C0406803 | CancerType | OtherBenign |
| Papillary syringaden oma | CUI | C0406803 | CancerType | OtherBenign |
| Papillary syringaden -oma | CUI | C0406803 | CancerType | OtherBenign |
| Papillary syringaden-oma | CUI | C0406803 | CancerType | OtherBenign |
| Papillary syringaden- oma | CUI | C0406803 | CancerType | OtherBenign |
| Pigmented, melanocytic naevus | CUI | C0027962 | CancerType | OtherBenign |
| Pigmented, melanocytic nevus | CUI | C0027962 | CancerType | OtherBenign |
| Pigmented, naevus | CUI | C0027962 | CancerType | OtherBenign |
| Pigmented, nevus | CUI | C0027962 | CancerType | OtherBenign |
| Pigmented hairy epidermal naevus | CUI | C0263579 | CancerType | OtherBenign |
| Pigmented hairy epidermal nevus | CUI | C0263579 | CancerType | OtherBenign |
| Pigmented naevus | CUI | C0027962 | CancerType | OtherBenign |
| Pigmented nevus | CUI | C0027962 | CancerType | OtherBenign |
| Pigmented spindle cell naevus of Reed | CUI | C0206738 | CancerType | OtherBenign |
| Pigmented spindle cell nevus of Reed | CUI | C0206738 | CancerType | OtherBenign |
| Pilar cyst | CUI | C2266788 | CancerType | OtherBenign |
| Pilomatrical carcinoma | CUI | C0585475 | CancerType | OtherCancerous |
| Pilomatrix carcinoma | CUI | C0585475 | CancerType | OtherCancerous |
| Pilomatrixoma | CUI | C0585475 | CancerType | OtherCancerous |
| Plexiform haemangioma | CUI | C4317089 | CancerType | OtherBenign |
| Plexiform haemangi oma | CUI | C4317089 | CancerType | OtherBenign |
| Plexiform haemangi -oma | CUI | C4317089 | CancerType | OtherBenign |
| Plexiform haemangi-oma | CUI | C4317089 | CancerType | OtherBenign |
| Plexiform haemangi- oma | CUI | C4317089 | CancerType | OtherBenign |
| Pre melanoma | CUI | C0854696 | CancerType | OtherInSitu |
| Pre-melanoma | CUI | C0854696 | CancerType | OtherInSitu |
| Primary cutaneous mucinous carcinoma | CUI | C1879641 | CancerType | OtherCancerous |
| Pseudomyogenic (epithelioid sarcoma -like) haemangioendothelioma | CUI | C3840252 | CancerType | OtherIntermediate |
| Pseudomyogenic (epithelioid sarcoma-like) haemangioendothelioma | CUI | C3840252 | CancerType | OtherIntermediate |
| Pseudomyogenic (epithelioid sarcoma- like) haemangioendothelioma | CUI | C3840252 | CancerType | OtherIntermediate |
| Pseudomyogenic haemangioendothelioma | CUI | C3840252 | CancerType | OtherIntermediate |
| Pyogenic granuloma | CUI | C0085653 | CancerType | OtherBenign |
| Pyogenic granul oma | CUI | C0085653 | CancerType | OtherBenign |
| Pyogenic granul -oma | CUI | C0085653 | CancerType | OtherBenign |
| Pyogenic granul-oma | CUI | C0085653 | CancerType | OtherBenign |
| Pyogenic granul- oma | CUI | C0085653 | CancerType | OtherBenign |
| Reed cell naevus | CUI | C0206738 | CancerType | OtherBenign |
| Reed cell nevus | CUI | C0206738 | CancerType | OtherBenign |
| Reed naevus | CUI | C0206738 | CancerType | OtherBenign |
| Reed nevus | CUI | C0206738 | CancerType | OtherBenign |
| Retiform haemangioendothelioma | CUI | C1304512 | CancerType | OtherIntermediate |
| Retiform haem angio endothelioma | CUI | C1304512 | CancerType | OtherIntermediate |
| Retiform haem angio endotheli oma | CUI | C1304512 | CancerType | OtherIntermediate |
| Retiform haem -angio -endothelioma | CUI | C1304512 | CancerType | OtherIntermediate |
| Retiform haem -angio -endotheli -oma | CUI | C1304512 | CancerType | OtherIntermediate |
| Retiform haem-angio-endothelioma | CUI | C1304512 | CancerType | OtherIntermediate |
| Retiform haem-angio-endotheli-oma | CUI | C1304512 | CancerType | OtherIntermediate |
| Retiform haem- angio- endothelioma | CUI | C1304512 | CancerType | OtherIntermediate |
| Retiform haem- angio- endotheli- oma | CUI | C1304512 | CancerType | OtherIntermediate |
| Rodent ulcer | CUI | C0007117 | CancerType | BasalCellCarcinoma |
| SCC in situ | CUI | C0006079 | Type | OtherInSitu |
| SCC in -situ | CUI | C0006079 | Type | OtherInSitu |
| SCC in-situ | CUI | C0006079 | Type | OtherInSitu |
| SCC in- situ | CUI | C0006079 | Type | OtherInSitu |
| Schwannoma | CUI | C0027809 | CancerType | OtherBenign |
| Sclerosing sweat duct carcinoma | CUI | C0346027 | CancerType | OtherCancerous |
| Sebaceous adenocarcinoma | CUI | C0206684 | CancerType | OtherCancerous |
| Sebaceous adenoma | CUI | C0585469 | CancerType | OtherBenign |
| Sebaceous aden oma | CUI | C0585469 | CancerType | OtherBenign |
| Sebaceous aden -oma | CUI | C0585469 | CancerType | OtherBenign |
| Sebaceous aden-oma | CUI | C0585469 | CancerType | OtherBenign |
| Sebaceous aden- oma | CUI | C0585469 | CancerType | OtherBenign |
| Sebaceous cyst | CUI | C0014511 | CancerType | OtherBenign |
| Sebaceous epithelioma | CUI | C2939441 | CancerType | OtherBenign |
| Sebaceous gland hyperplasia | CUI | C0406484 | CancerType | OtherBenign |
| Sebaceous hyperplasia | CUI | C0406484 | CancerType | OtherBenign |
| Sebaceous naevus | CUI | C3854181 | CancerType | OtherBenign |
| Sebaceous nevus | CUI | C3854181 | CancerType | OtherBenign |
| Seborrhoeic keratosis | CUI | C0022603 | CancerType | OtherBenign |
| Senile angioma | CUI | C0343082 | CancerType | OtherBenign |
| Senile angi oma | CUI | C0343082 | CancerType | OtherBenign |
| Senile angi -oma | CUI | C0343082 | CancerType | OtherBenign |
| Senile angi-oma | CUI | C0343082 | CancerType | OtherBenign |
| Senile angi- oma | CUI | C0343082 | CancerType | OtherBenign |
| Skin appendage adenoma | CUI | C0334342 | CancerType | OtherBenign |
| Skin tag | CUI | C0037293 | CancerType | OtherBenign |
| Small congenital naevus | CUI | C1883045 | CancerType | OtherBenign |
| Small congenital nevus | CUI | C1883045 | CancerType | OtherBenign |
| Solar keratosis | CUI | C0006079 | Type | OtherInSitu |
| Solar lentigo | CUI | C0036651 | CancerType | OtherBenign |
| Spindle cell angioendothelioma | CUI | C1304508 | CancerType | OtherIntermediate |
| Spindle cell angio endothelioma | CUI | C1304508 | CancerType | OtherIntermediate |
| Spindle cell angio -endothelioma | CUI | C1304508 | CancerType | OtherIntermediate |
| Spindle cell angio-endothelioma | CUI | C1304508 | CancerType | OtherIntermediate |
| Spindle cell angio- endothelioma | CUI | C1304508 | CancerType | OtherIntermediate |
| Spindle cell haemangioendothelioma | CUI | C1304508 | CancerType | OtherIntermediate |
| Spindle cell haem -angioendothelioma | CUI | C1304508 | CancerType | OtherIntermediate |
| Spindle cell haem-angioendothelioma | CUI | C1304508 | CancerType | OtherIntermediate |
| Spindle cell haem- angioendothelioma | CUI | C1304508 | CancerType | OtherIntermediate |
| Spindle cell haemangioma | CUI | C1304508 | CancerType | OtherBenign |
| Spindle cell haemangi oma | CUI | C1304508 | CancerType | OtherBenign |
| Spindle cell haemangi -oma | CUI | C1304508 | CancerType | OtherBenign |
| Spindle cell haemangi-oma | CUI | C1304508 | CancerType | OtherBenign |
| Spindle cell haemangi- oma | CUI | C1304508 | CancerType | OtherBenign |
| Spindle cell malignant melanoma | CUI | C0334444 | CancerType | OtherCancerous |
| Spindle cell melanoma | CUI | C0334444 | CancerType | OtherCancerous |
| Spindle cell naevus | CUI | C0206738 | CancerType | OtherBenign |
| Spindle cell nevus | CUI | C0206738 | CancerType | OtherBenign |
| Spitz naevus | CUI | C0206739 | CancerType | OtherBenign |
| Spitz nevus | CUI | C0206739 | CancerType | OtherBenign |
| Squamous carcinoma | CUI | C0553723 | CancerType | OtherCancerous |
| Squamous cell carcinoma | CUI | C0553723 | CancerType | OtherCancerous |
| Squamous cell carcinoma in situ | CUI | C0006079 | Type | OtherInSitu |
| Squamous cell carcinoma in -situ | CUI | C0006079 | Type | OtherInSitu |
| Squamous cell carcinoma in-situ | CUI | C0006079 | Type | OtherInSitu |
| Squamous cell carcinoma in- situ | CUI | C0006079 | Type | OtherInSitu |
| Squamous papilloma | CUI | C0345983 | CancerType | OtherBenign |
| Squamous papill oma | CUI | C0345983 | CancerType | OtherBenign |
| Squamous papill -oma | CUI | C0345983 | CancerType | OtherBenign |
| Squamous papill-oma | CUI | C0345983 | CancerType | OtherBenign |
| Squamous papill- oma | CUI | C0345983 | CancerType | OtherBenign |
| Strawberry naevus | CUI | C4317089 | CancerType | OtherBenign |
| Strawberry nevus | CUI | C4317089 | CancerType | OtherBenign |
| Subungal malignant melanoma | CUI | C0346037 | CancerType | OtherCancerous |
| Sub ungal malignant melanoma | CUI | C0346037 | CancerType | OtherCancerous |
| Sub -ungal malignant melanoma | CUI | C0346037 | CancerType | OtherCancerous |
| Sub-ungal malignant melanoma | CUI | C0346037 | CancerType | OtherCancerous |
| Sub- ungal malignant melanoma | CUI | C0346037 | CancerType | OtherCancerous |
| Subungal melanoma | CUI | C0346037 | CancerType | OtherCancerous |
| Sub ungal melanoma | CUI | C0346037 | CancerType | OtherCancerous |
| Sub -ungal melanoma | CUI | C0346037 | CancerType | OtherCancerous |
| Sub-ungal melanoma | CUI | C0346037 | CancerType | OtherCancerous |
| Sub- ungal melanoma | CUI | C0346037 | CancerType | OtherCancerous |
| Superficial spreading malignant melanoma | CUI | C0334438 | CancerType | OtherCancerous |
| Superficial spreading melanoma | CUI | C0334438 | CancerType | OtherCancerous |
| Superficial spreading melanoma in situ | CUI | C0854696 | CancerType | OtherInSitu |
| Sweat gland adenocarcinoma | CUI | C0334344 | CancerType | OtherCancerous |
| Sweat gland adenoma | CUI | C0019522 | CancerType | OtherBenign |
| Syringocystadenoma papilliferum | CUI | C0406803 | CancerType | OtherBenign |
| Syringo cystadenoma papilliferum | CUI | C0406803 | CancerType | OtherBenign |
| Syringo -cystadenoma papilliferum | CUI | C0406803 | CancerType | OtherBenign |
| Syringo-cystadenoma papilliferum | CUI | C0406803 | CancerType | OtherBenign |
| Syringo- cystadenoma papilliferum | CUI | C0406803 | CancerType | OtherBenign |
| Syringofibroadenoma | CUI | C1266060 | CancerType | OtherBenign |
| Syringofibroaden oma | CUI | C1266060 | CancerType | OtherBenign |
| Syringofibroaden -oma | CUI | C1266060 | CancerType | OtherBenign |
| Syringofibroaden-oma | CUI | C1266060 | CancerType | OtherBenign |
| Syringofibroaden- oma | CUI | C1266060 | CancerType | OtherBenign |
| Syringo fibroadenoma | CUI | C1266060 | CancerType | OtherBenign |
| Syringo fibroaden oma | CUI | C1266060 | CancerType | OtherBenign |
| Syringo fibroaden -oma | CUI | C1266060 | CancerType | OtherBenign |
| Syringo fibroaden-oma | CUI | C1266060 | CancerType | OtherBenign |
| Syringo fibroaden- oma | CUI | C1266060 | CancerType | OtherBenign |
| Syringo -fibroadenoma | CUI | C1266060 | CancerType | OtherBenign |
| Syringo -fibroaden oma | CUI | C1266060 | CancerType | OtherBenign |
| Syringo -fibroaden -oma | CUI | C1266060 | CancerType | OtherBenign |
| Syringo-fibroadenoma | CUI | C1266060 | CancerType | OtherBenign |
| Syringo-fibroaden oma | CUI | C1266060 | CancerType | OtherBenign |
| Syringo-fibroaden-oma | CUI | C1266060 | CancerType | OtherBenign |
| Syringo- fibroadenoma | CUI | C1266060 | CancerType | OtherBenign |
| Syringo- fibroaden oma | CUI | C1266060 | CancerType | OtherBenign |
| Syringo- fibroaden- oma | CUI | C1266060 | CancerType | OtherBenign |
| Syringoma | CUI | C0206673 | CancerType | OtherBenign |
| Syringomatous carcinoma | CUI | C0346027 | CancerType | OtherCancerous |
| Syringomatous sweat duct carcinoma | CUI | C0346027 | CancerType | OtherCancerous |
| Syringomatous tumour | CUI | C3839745 | CancerType | OtherBenign |
| Trichilemmal cyst | CUI | C2266788 | CancerType | OtherBenign |
| Trichilemmoma | CUI | C0334263 | CancerType | OtherBenign |
| Trichoblastoma | CUI | C1299879 | CancerType | OtherBenign |
| Tricho blastoma | CUI | C1299879 | CancerType | OtherBenign |
| Tricho -blastoma | CUI | C1299879 | CancerType | OtherBenign |
| Tricho-blastoma | CUI | C1299879 | CancerType | OtherBenign |
| Tricho- blastoma | CUI | C1299879 | CancerType | OtherBenign |
| Trichoepithelioma | CUI | C0349658 | CancerType | OtherBenign |
| Tricho epithelioma | CUI | C0349658 | CancerType | OtherBenign |
| Tricho -epithelioma | CUI | C0349658 | CancerType | OtherBenign |
| Tricho-epithelioma | CUI | C0349658 | CancerType | OtherBenign |
| Tricho- epithelioma | CUI | C0349658 | CancerType | OtherBenign |
| Trichoepitheliomatous | CUI | C0334262 | CancerType | OtherBenign |
| Trichofolliculoma | CUI | C0334262 | CancerType | OtherBenign |
| Tricho folliculoma | CUI | C0334262 | CancerType | OtherBenign |
| Tricho -folliculoma | CUI | C0334262 | CancerType | OtherBenign |
| Tricho-folliculoma | CUI | C0334262 | CancerType | OtherBenign |
| Tricho- folliculoma | CUI | C0334262 | CancerType | OtherBenign |
| Tufted angioma | CUI | C0346073 | CancerType | OtherBenign |
| Tufted angi oma | CUI | C0346073 | CancerType | OtherBenign |
| Tufted angi -oma | CUI | C0346073 | CancerType | OtherBenign |
| Tufted angi-oma | CUI | C0346073 | CancerType | OtherBenign |
| Tufted angi- oma | CUI | C0346073 | CancerType | OtherBenign |
| Tufted haemangioma | CUI | C1266161 | CancerType | OtherBenign |
| Tufted haemangi oma | CUI | C1266161 | CancerType | OtherBenign |
| Tufted haemangi -oma | CUI | C1266161 | CancerType | OtherBenign |
| Tufted haemangi-oma | CUI | C1266161 | CancerType | OtherBenign |
| Tufted haemangi- oma | CUI | C1266161 | CancerType | OtherBenign |
| Venous haemangioma | CUI | C0334532 | CancerType | OtherBenign |
| Verruca | CUI | C0043037 | CancerType | OtherBenign |
| Verrucous keratotic haemangioma | CUI | C0334540 | CancerType | OtherBenign |
| Verrucous keratotic haemangi oma | CUI | C0334540 | CancerType | OtherBenign |
| Verrucous keratotic haemangi -oma | CUI | C0334540 | CancerType | OtherBenign |
| Verrucous keratotic haemangi-oma | CUI | C0334540 | CancerType | OtherBenign |
| Verrucous keratotic haemangi- oma | CUI | C0334540 | CancerType | OtherBenign |
| Verrucous papilloma | CUI | C0334243 | CancerType | OtherBenign |
| Verrucous papill oma | CUI | C0334243 | CancerType | OtherBenign |
| Verrucous papill -oma | CUI | C0334243 | CancerType | OtherBenign |
| Verrucous papill-oma | CUI | C0334243 | CancerType | OtherBenign |
| Verrucous papill- oma | CUI | C0334243 | CancerType | OtherBenign |
| Verrucous squamous cell carcinoma | CUI | C0206706 | CancerType | OtherCancerous |
| Viral wart | CUI | C0043037 | CancerType | OtherBenign |
| Xanthelasma | CUI | C0302314 | CancerType | OtherBenign |
| Xanthoma | CUI | C0302314 | CancerType | OtherBenign |
| Xanth oma | CUI | C0302314 | CancerType | OtherBenign |
| Xanth -oma | CUI | C0302314 | CancerType | OtherBenign |
| Xanth-oma | CUI | C0302314 | CancerType | OtherBenign |
| Xanth- oma | CUI | C0302314 | CancerType | OtherBenign |
| Xanthomata | CUI | C0302314 | CancerType | OtherBenign |
| Xantho mata | CUI | C0302314 | CancerType | OtherBenign |
| Xantho -mata | CUI | C0302314 | CancerType | OtherBenign |
| Xantho-mata | CUI | C0302314 | CancerType | OtherBenign |
| Xantho- mata | CUI | C0302314 | CancerType | OtherBenign |

## *Diameter.lst*

| **Value** |
| --- |
| Diameter |

## *Differentiation.lst*

| **Value** | **Feature 1** | **Value 1** |
| --- | --- | --- |
| Adamantinoid | Differentiation | Adamantinoid |
| Adenoid | Differentiation | Adenoid |
| Adnexal | Differentiation | Adnexal |
| Apocrine | Differentiation | Apocrine |
| Clear Cell | Differentiation | Clear Cell |
| Ductal Glandular | Differentiation | Ductal Glandular |
| Eccrine | Differentiation | Eccrine |
| Follicular | Differentiation | Follicular |
| Giant Cell | Differentiation | Giant Cell |
| Granular Cell | Differentiation | Granular Cell |
| Infundibulocystic | Differentiation | Infundibulocystic |
| Infundibulo cystic | Differentiation | Infundibulocystic |
| Infundibulo-cystic | Differentiation | Infundibulocystic |
| Keratotic | Differentiation | Keratotic |
| Matricial | Differentiation | Matricial |
| Myoepithelial | Differentiation | Myoepithelial |
| Neuroendocrine | Differentiation | Neuroendocrine |
| Neuro endocrine | Differentiation | Neuroendocrine |
| Neuro-endocrine | Differentiation | Neuroendocrine |
| Pleiomorphic | Differentiation | Pleiomorphic |
| Plieomorphic | Differentiation | Pleiomorphic |
| Sebaceous | Differentiation | Sebaceous |
| Signet Cell | Differentiation | Signet Cell |
| Squamous | Differentiation | Squamous |

## *Dimensions.lst*

| **Value** | **Feature 1** | **Value 1** |
| --- | --- | --- |
| Deep | Dimension | Thickness |
| Diameter | Dimension | Width |
| Length | Dimension | Length |
| Long | Dimension | Length |
| Thick | Dimension | Thickness |
| Thickness | Dimension | Thickness |
| Underlying fatty tissue | Dimension | Thickness |
| Underlying tissue | Dimension | Thickness |
| Wide | Dimension | Width |
| Width | Dimension | Width |
| Width (lower half) | Dimension | Width |
| Width (upper half) | Dimension | Width |

## *Distance_to.lst*

| **Value** |
| --- |
| Distance to |

## *doubleTag.lst*

| **Value** | **Feature 1** | **Value 1** |
| --- | --- | --- |
| A-B) | len | 2 |
| A-C) | len | 3 |
| A-D) | len | 4 |
| A-E) | len | 5 |
| A-F) | len | 6 |
| B-C) | len | 2 |
| B-D) | len | 3 |
| B-E) | len | 4 |
| B-F) | len | 5 |
| C-D) | len | 2 |
| C-E) | len | 3 |
| C-F) | len | 4 |
| D-E) | len | 2 |
| D-F) | len | 3 |
| E-F) | len | 2 |

## *ExcisionCompleted.lst*

| **Value** | **Feature 1** | **Value 1** |
| --- | --- | --- |
| Appears complete | Negatives | true |
| Appears completed | Negatives | true |
| Appropriately excised | Negatives | true |
| Clear margins | Negatives | true |
| Completed excision | Negatives | true |
| Complete excision | Negatives | true |
| Complete excision cannnot be assured | Negatives | false |
| Complete excision cannnot be guaranteed | Negatives | false |
| Complete excision is assured | Negatives | true |
| Complete excision is not guaranteed | Negatives | true |
| Completely excised | Negatives | true |
| Completeness of excision cannnot be guaranteed | Negatives | false |
| Completeness of excision is assured | Negatives | true |
| Completeness of excision is not assured | Negatives | true |
| Completeness of excision is not guaranteed | Negatives | true |
| Excision cannnot be assured | Negatives | false |
| Excision complete | Negatives | true |
| Excision completed | Negatives | true |
| Excision incomplete | Negatives | false |
| Excision incompleted | Negatives | false |
| Excision is appropriate | Negatives | true |
| Excision is assured | Negatives | true |
| Excision is complete | Negatives | true |
| Excision is guaranteed | Negatives | true |
| Excision is inappropriate | Negatives | false |
| Excision is incomplete | Negatives | false |
| Excision is incompleted | Negatives | false |
| Excision is not appropriate | Negatives | true |
| Excision is not complete | Negatives | true |
| Excision is not guaranteed | Negatives | true |
| Inappropriate excision | Negatives | false |
| Inappropriately excised | Negatives | false |
| Incomplete excision | Negatives | false |
| Incompletely excised | Negatives | false |
| Look complete | Negatives | true |
| Look completed | Negatives | true |
| Looks complete | Negatives | true |
| Looks completed | Negatives | true |
| Margins are clear | Negatives | true |
| Margins are clear | Negatives | true |
| Margins are not clear | Negatives | true |

Negatives = true, negation is true; negatives = false, negation is false.

## *ExcisionNature.lst*

| **Value** | **Feature 1** | **Value 1** |
| --- | --- | --- |
| Incomplete | ExcisionNature | PreviouslyIncomplete |
| Incompletely excised | ExcisionNature | PreviouslyIncomplete |
| Old biopsy | ExcisionNature | Recurrent |
| Old biopsy site | ExcisionNature | Recurrent |
| Old flap | ExcisionNature | Recurrent |
| Old FTSG | ExcisionNature | Recurrent |
| Old full thickness graft | ExcisionNature | Recurrent |
| Old full thickness skin graft | ExcisionNature | Recurrent |
| Old graft | ExcisionNature | Recurrent |
| Old local flap | ExcisionNature | Recurrent |
| Old scar | ExcisionNature | Recurrent |
| Old site | ExcisionNature | Recurrent |
| Old skin graft | ExcisionNature | Recurrent |
| Old split thickness graft | ExcisionNature | Recurrent |
| Old split thickness skin graft | ExcisionNature | Recurrent |
| Old SSG | ExcisionNature | Recurrent |
| Old STSG | ExcisionNature | Recurrent |
| Previous Incomplete | ExcisionNature | PreviouslyIncomplete |
| Previous Incomplete excision | ExcisionNature | PreviouslyIncomplete |
| Previous Incompletely excised | ExcisionNature | PreviouslyIncomplete |
| Previously Incomplete | ExcisionNature | PreviouslyIncomplete |
| Previously Incompletely excised | ExcisionNature | PreviouslyIncomplete |
| Primary | ExcisionNature | Primary |
| Recurrence | ExcisionNature | Recurrent |
| Recurrent | ExcisionNature | Recurrent |

## *ExcisionType.lst*

| **Value** | **Feature 1** | **Value 1** |
| --- | --- | --- |
| Curettage | ExcisionType | Curettage |
| Deeper Excision | ExcisionType | Re-excision |
| Deeper Margin | ExcisionType | SupplementalDeepMarginSpecimen |
| Deeper Margin | ExcisionType | SupplementalDeepMarginSpecimen |
| Deeper Margin Specimen | ExcisionType | SupplementalDeepMarginSpecimen |
| Deeper Margin Specimens | ExcisionType | SupplementalDeepMarginSpecimen |
| Deeper Specimen | ExcisionType | SupplementalDeepMarginSpecimen |
| Deeper Specimens | ExcisionType | SupplementalDeepMarginSpecimen |
| Deep Margin Specimen | ExcisionType | SupplementalDeepMarginSpecimen |
| Deep Margin Specimens | ExcisionType | SupplementalDeepMarginSpecimen |
| Excision | ExcisionType | ExcisionBiopsy |
| Excision Biopsy | ExcisionType | ExcisionBiopsy |
| Excision Bx | ExcisionType | ExcisionBiopsy |
| Further Deeper Margin | ExcisionType | SupplementalDeepMarginSpecimen |
| Further Deeper Margin | ExcisionType | SupplementalDeepMarginSpecimen |
| Further Deeper Margin Specimen | ExcisionType | SupplementalDeepMarginSpecimen |
| Further Deeper Margin Specimens | ExcisionType | SupplementalDeepMarginSpecimen |
| Further Deeper Specimen | ExcisionType | SupplementalDeepMarginSpecimen |
| Further Deeper Specimens | ExcisionType | SupplementalDeepMarginSpecimen |
| Further Deep Margin Specimen | ExcisionType | SupplementalDeepMarginSpecimen |
| Further Deep Margin Specimens | ExcisionType | SupplementalDeepMarginSpecimen |
| Further excision | ExcisionType | Re-excision |
| Further Peripheral Margin Specimen | ExcisionType | SupplementalPeripheralMarginSpecimen |
| Further Peripheral Margin Specimens | ExcisionType | SupplementalPeripheralMarginSpecimen |
| Further Wider Margin | ExcisionType | SupplementalPeripheralMarginSpecimen |
| Further Wider Margin | ExcisionType | SupplementalPeripheralMarginSpecimen |
| Further Wider Margin Specimen | ExcisionType | SupplementalPeripheralMarginSpecimen |
| Further Wider Margin Specimens | ExcisionType | SupplementalPeripheralMarginSpecimen |
| Further Wider Specimen | ExcisionType | SupplementalPeripheralMarginSpecimen |
| Further Wider Specimens | ExcisionType | SupplementalPeripheralMarginSpecimen |
| Incision Biopsy | ExcisionType | IncisionBiopsy |
| Incision Bx | ExcisionType | IncisionBiopsy |
| Mapping biopsy | ExcisionType | PunchBiopsy |
| Mapping bx | ExcisionType | PunchBiopsy |
| Moh's | ExcisionType | Mohs |
| Moh's micrographic surgery | ExcisionType | Mohs |
| Moh's surgery | ExcisionType | Mohs |
| Mohs | ExcisionType | Mohs |
| Mohs micrographic surgery | ExcisionType | Mohs |
| Mohs surgery | ExcisionType | Mohs |
| Narrow margin excision | ExcisionType | ExcisionBiopsy |
| Peripheral Margin Specimen | ExcisionType | SupplementalPeripheralMarginSpecimen |
| Peripheral Margin Specimens | ExcisionType | SupplementalPeripheralMarginSpecimen |
| Punch | ExcisionType | PunchBiopsy |
| Punch Biopsy | ExcisionType | PunchBiopsy |
| Punch Bx | ExcisionType | PunchBiopsy |
| Re excision | ExcisionType | Re-excision |
| Re-excision | ExcisionType | Re-excision |
| Second Deeper Margin | ExcisionType | SupplementalDeepMarginSpecimen |
| Second Deeper Margin | ExcisionType | SupplementalDeepMarginSpecimen |
| Second Deeper Margin Specimen | ExcisionType | SupplementalDeepMarginSpecimen |
| Second Deeper Margin Specimens | ExcisionType | SupplementalDeepMarginSpecimen |
| Second Deeper Specimen | ExcisionType | SupplementalDeepMarginSpecimen |
| Second Deeper Specimens | ExcisionType | SupplementalDeepMarginSpecimen |
| Second Deep Margin Specimen | ExcisionType | SupplementalDeepMarginSpecimen |
| Second Deep Margin Specimens | ExcisionType | SupplementalDeepMarginSpecimen |
| Second Peripheral Margin Specimen | ExcisionType | SupplementalPeripheralMarginSpecimen |
| Second Peripheral Margin Specimens | ExcisionType | SupplementalPeripheralMarginSpecimen |
| Second Wider Margin | ExcisionType | SupplementalPeripheralMarginSpecimen |
| Second Wider Margin | ExcisionType | SupplementalPeripheralMarginSpecimen |
| Second Wider Margin Specimen | ExcisionType | SupplementalPeripheralMarginSpecimen |
| Second Wider Margin Specimens | ExcisionType | SupplementalPeripheralMarginSpecimen |
| Second Wider Specimen | ExcisionType | SupplementalPeripheralMarginSpecimen |
| Second Wider Specimens | ExcisionType | SupplementalPeripheralMarginSpecimen |
| Shave | ExcisionType | ShaveBiopsy |
| Shave Biopsy | ExcisionType | ShaveBiopsy |
| Shave Bx | ExcisionType | ShaveBiopsy |
| Subsequent Deeper Margin | ExcisionType | SupplementalDeepMarginSpecimen |
| Subsequent Deeper Margin | ExcisionType | SupplementalDeepMarginSpecimen |
| Subsequent Deeper Margin Specimen | ExcisionType | SupplementalDeepMarginSpecimen |
| Subsequent Deeper Margin Specimens | ExcisionType | SupplementalDeepMarginSpecimen |
| Subsequent Deeper Specimen | ExcisionType | SupplementalDeepMarginSpecimen |
| Subsequent Deeper Specimens | ExcisionType | SupplementalDeepMarginSpecimen |
| Subsequent Deep Margin Specimen | ExcisionType | SupplementalDeepMarginSpecimen |
| Subsequent Deep Margin Specimens | ExcisionType | SupplementalDeepMarginSpecimen |
| Subsequent Peripheral Margin Specimen | ExcisionType | SupplementalPeripheralMarginSpecimen |
| Subsequent Peripheral Margin Specimens | ExcisionType | SupplementalPeripheralMarginSpecimen |
| Subsequent Wider Margin | ExcisionType | SupplementalPeripheralMarginSpecimen |
| Subsequent Wider Margin | ExcisionType | SupplementalPeripheralMarginSpecimen |
| Subsequent Wider Margin Specimen | ExcisionType | SupplementalPeripheralMarginSpecimen |
| Subsequent Wider Margin Specimens | ExcisionType | SupplementalPeripheralMarginSpecimen |
| Subsequent Wider Specimen | ExcisionType | SupplementalPeripheralMarginSpecimen |
| Subsequent Wider Specimens | ExcisionType | SupplementalPeripheralMarginSpecimen |
| Supplemental Deeper Margin | ExcisionType | SupplementalDeepMarginSpecimen |
| Supplemental Deeper Margin | ExcisionType | SupplementalDeepMarginSpecimen |
| Supplemental Deeper Margin Specimen | ExcisionType | SupplementalDeepMarginSpecimen |
| Supplemental Deeper Margin Specimens | ExcisionType | SupplementalDeepMarginSpecimen |
| Supplemental Deeper Specimen | ExcisionType | SupplementalDeepMarginSpecimen |
| Supplemental Deeper Specimens | ExcisionType | SupplementalDeepMarginSpecimen |
| Supplemental Deep Margin Specimen | ExcisionType | SupplementalDeepMarginSpecimen |
| Supplemental Deep Margin Specimens | ExcisionType | SupplementalDeepMarginSpecimen |
| Supplemental Peripheral Margin Specimen | ExcisionType | SupplementalPeripheralMarginSpecimen |
| Supplemental Peripheral Margin Specimens | ExcisionType | SupplementalPeripheralMarginSpecimen |
| Supplemental Wider Margin | ExcisionType | SupplementalPeripheralMarginSpecimen |
| Supplemental Wider Margin | ExcisionType | SupplementalPeripheralMarginSpecimen |
| Supplemental Wider Margin Specimen | ExcisionType | SupplementalPeripheralMarginSpecimen |
| Supplemental Wider Margin Specimens | ExcisionType | SupplementalPeripheralMarginSpecimen |
| Supplemental Wider Specimen | ExcisionType | SupplementalPeripheralMarginSpecimen |
| Supplemental Wider Specimens | ExcisionType | SupplementalPeripheralMarginSpecimen |
| Wedge | ExcisionType | WedgeExcision |
| Wedge Excision | ExcisionType | WedgeExcision |
| Wide Excision | ExcisionType | WideLocalExcision |
| Wide Local Excision | ExcisionType | WideLocalExcision |
| Wider Excision | ExcisionType | Re-excision |
| Wider local Excision | ExcisionType | Re-excision |
| Wider Margin | ExcisionType | SupplementalPeripheralMarginSpecimen |
| Wider Margin | ExcisionType | SupplementalPeripheralMarginSpecimen |
| Wider Margin Specimen | ExcisionType | SupplementalPeripheralMarginSpecimen |
| Wider Margin Specimens | ExcisionType | SupplementalPeripheralMarginSpecimen |
| Wider Specimen | ExcisionType | SupplementalPeripheralMarginSpecimen |
| Wider Specimens | ExcisionType | SupplementalPeripheralMarginSpecimen |
| WL | ExcisionType | WideLocalExcision |

## *Frozen_Section_Triggers.lst*

| **Value** |
| --- |
| Skin containing |
| Skin with |

## *Frozen_Section.lst*

| **Value** |
| --- |
| Frozensection |
| Frozen section |

## *Lesion.lst*

| **Value** |
| --- |
| Approximately |
| Approximately  measuring |
| Area |
| Area measuring |
| Areas |
| Areas measuring |
| Central |
| Central measuring |
| Irregular |
| Irregular area |
| Irregular area measuring |
| Irregular areas |
| Irregular areas measuring |
| Irregular measuring |
| Irregular Nodule |
| Irregular Nodule measuring |
| Irregular plaque |
| Irregular plaque measuring |
| Irregular surface |
| Irregular surface measuring |
| Irregular ulcer |
| Irregular ulcer measuring |
| Lesion |
| Lesion measuring |
| Lesions |
| Lesions measuring |
| Nodule |
| Nodule |
| Nodule  measuring |
| Nodules |
| Nodules measuring |
| Plaque |
| Plaque measuring |
| Plaques |
| Plaques measuring |
| Raised |
| Raised measuring |
| Surface |
| Surface  measuring |
| Surfaces |
| Surfaces measuring |
| Ulcer |
| Ulcerated |
| Ulcerated  measuring |
| Ulcer measuring |
| Ulcers |

## *LevelOfInvasionTrigger.lst*

| **Value** | **Feature 1** | **Value 1** |
| --- | --- | --- |
| Abuting | Negatives | true |
| Abuts | Negatives | true |
| Abuts but does not invade | Negatives | false |
| Abuts but not invading | Negatives | false |
| Abutting | Negatives | true |
| Abuttment | Negatives | true |
| Abutts | Negatives | true |
| Abutts but does not invade | Negatives | false |
| Abutts but not invading | Negatives | false |
| Confined | Negatives | true |
| Extending into | Negatives | true |
| Extending to | Negatives | true |
| Extension | Negatives | true |
| Extension into | Negatives | true |
| Focally reaching | Negatives | true |
| Infiltrating underlying | Negatives | true |
| Invades | Negatives | true |
| Invades into | Negatives | true |
| Invading | Negatives | true |
| Invading into | Negatives | true |
| Invasion | Negatives | true |
| Invasion into | Negatives | true |
| Involved | Negatives | true |
| Involved with | Negatives | true |
| Involving | Negatives | true |
| Penetrates | Negatives | true |
| Reaching | Negatives | true |
| Tethered to | Negatives | true |
| Within | Negatives | true |

## *LVI.lst*

| **Value** |
| --- |
| LVI |
| LVSI |
| LymphovascularInvasion |
| Lymphovascular Invasion |
| Lympho vascularInvasion |
| Lympho vascular invasion |
| Lympho -vascular invasion |
| Lympho-vascular invasion |
| Lympho- vascular invasion |
| Lympho vascular space invasion |
| LymphovascularSpread |
| Lymphovascular Spread |
| Lympho vascularSpread |
| Lympho vascular Spread |
| Lympho -vascular Spread |
| Lympho-vascular Spread |
| Lympho- vascular Spread |

## *LVIorPNI.lst*

| **Value** | **Feature 1** | **Value 1** |
| --- | --- | --- |
| LV | LVI | Yes |
| Lymphovascular | LVI | Yes |
| Lympho vascular | LVI | Yes |
| Lympho-vascular | LVI | Yes |
| Perineural | PNI | Yes |
| Peri neural | PNI | Yes |
| Peri -neural | PNI | Yes |
| Peri-neural | PNI | Yes |
| Peri- neural | PNI | Yes |
| PN | PNI | Yes |

## *MarginWordDistances.lst*

| **Value** | **Feature 1** | **Value 1** |
| --- | --- | --- |
| At the margin | Value | 0 |
| Extending | Value | 0 |
| Extends | Value | 0 |
| Inappropriate | Value | 0 |
| Involved | Value | 0 |
| Involves | Value | 0 |
| Involving | Value | 0 |
| Positive | Value | 0 |
| Present | Value | 0 |
| Preset | Value | 0 |

## *MedialOrLateral.lst*

| **Value** | **Feature 1** | **Value 1** |
| --- | --- | --- |
| Lateral | MedialOrLateral | Lateral |
| Medial | MedialOrLateral | Medial |

## *Micro deep margin.lst*

| **Value** |
| --- |
| Deep |
| Deep clearance |
| Deep clearances |
| Deep excision |
| Deep excisions |
| Deeply |
| Deep margin |
| Deep margins |
| Subcutaneous (deep) margin |
| Subcutaneous margin |

## *Micro peripheral margin.lst*

| **Value** |
| --- |
| Black |
| Black ink |
| Black inked |
| Blue |
| Blue ink |
| Blue inked |
| Epidermal clearance |
| Epidermal clearances |
| Epidermal margin |
| Epidermal margins |
| Green |
| Green ink |
| Green inked |
| Lateral clearance |
| Lateral clearances |
| Lateral excision |
| Lateral excision |
| Lateral excisions |
| Lateral excisions |
| Lateral margin |
| Lateral margins |
| Margin laterally |
| Margin peripherally |
| Margin radially |
| Peripheral clearance |
| Peripheral clearances |
| Peripheral excision |
| Peripheral excisions |
| Peripheral margin |
| Peripheral margins |
| Radial clearance |
| Radial clearances |
| Radial excision |
| Radial excisions |
| Radial margin |
| Radial margins |
| Red |
| Red ink |
| Red inked |
| Yellow |
| Yellow ink |
| Yellow inked |

## *Micro_deep_margin_maybe.lst*

| **Value** |
| --- |
| Other margin |
| Other margins |

## *Micro_peripheral_margin_maybe.lst*

| **Value** |
| --- |
| Circumferential |
| Epidermal |
| Lateral |
| Peripheral |
| Radial |
| RM |

## *MultipleScalp.lst*

| **Value** | **Feature 1** | **Value 1** |
| --- | --- | --- |
| Frontal | MultipleScalp | Frontal |
| Frontal parietal temporal occipital | MultipleScalp | PanScalp |
| Frontal temporal occipital parietal | MultipleScalp | PanScalp |
| Frontoparietal | MultipleScalp | Fronto-parietal |
| Fronto parietal | MultipleScalp | Fronto-parietal |
| Fronto -parietal | MultipleScalp | Fronto-parietal |
| Fronto-parietal | MultipleScalp | Fronto-parietal |
| Fronto- parietal | MultipleScalp | Fronto-parietal |
| Fronto parieto temporo occipital | MultipleScalp | PanScalp |
| Fronto -parieto-temporo occipital | MultipleScalp | PanScalp |
| Fronto-parieto-temporo occipital | MultipleScalp | PanScalp |
| Fronto- parieto-temporo occipital | MultipleScalp | PanScalp |
| Frontotemporal | MultipleScalp | Fronto-temporal |
| Fronto temporal | MultipleScalp | Fronto-temporal |
| Fronto -temporal | MultipleScalp | Fronto-temporal |
| Fronto-temporal | MultipleScalp | Fronto-temporal |
| Fronto- temporal | MultipleScalp | Fronto-temporal |
| Fronto temporo occipito parietal | MultipleScalp | PanScalp |
| Fronto -temporo-occipito parietal | MultipleScalp | PanScalp |
| Fronto-temporo-occipito parietal | MultipleScalp | PanScalp |
| Fronto- temporo-occipito parietal | MultipleScalp | PanScalp |
| Nuchal | MultipleScalp | Nuchal |
| Occipital | MultipleScalp | Occipital |
| Parietal | MultipleScalp | Parietal |
| Temporal | MultipleScalp | Temporal |
| Vertex | MultipleScalp | Vertex |
| Vertex of | MultipleScalp | Vertex |

## *o'clock.lst*

| **Value** |
| --- |
| o?clock |
| o? clock |
| o ?clock |
| o ? clock |
| o/clock |
| o/ clock |
| o /clock |
| o / clock |
| o'clock |
| o' clock |
| o 'clock |
| o ' clock |

## *PNI.lst*

| **Value** |
| --- |
| PerineuralInvasion |
| Perineural Invasion |
| Peri neuralInvasion |
| Peri neural Invasion |
| Peri -neural Invasion |
| Peri-neural Invasion |
| Peri- neural Invasion |
| Perineural space invasion |
| Peri neural space invasion |
| Peri-neural space invasion |
| PerineuralSpread |
| Perineural Spread |
| Peri neuralSpread |
| Peri neural Spread |
| Peri -neural Spread |
| Peri-neural Spread |
| Peri- neural Spread |
| PNI |
| PNSI |

## *ProximalOrDistal.lst*

| **Value** | **Feature 1** | **Value 1** |
| --- | --- | --- |
| Caudal | ProximalOrDistal | Distal |
| Cranial | ProximalOrDistal | Proximal |
| Distal | ProximalOrDistal | Distal |
| Inferior | ProximalOrDistal | Distal |
| Proximal | ProximalOrDistal | Proximal |
| Superior | ProximalOrDistal | Proximal |

## *RecurrentDisease.lst*

| **Value** | **Feature 1** | **Value 1** |
| --- | --- | --- |
| Evidence of Recurrent cancer | Negatives | true |
| Evidence of Recurrent Disease | Negatives | true |
| Evidence of Recurrent malignancy | Negatives | true |
| Evidence of Recurrent tumour | Negatives | true |
| Presence of cancer disease | Negatives | true |
| Presence of disease | Negatives | true |
| Presence of malignant disease | Negatives | true |
| Presence of Recurrent disease | Negatives | true |
| Recurrent | Negatives | true |
| Recurrent/residual tumour | Negatives | true |
| Recurrent cancer | Negatives | true |
| Recurrent cancer absent | Negatives | false |
| Recurrent cancer Evident | Negatives | true |
| Recurrent cancer not Present | Negatives | false |
| Recurrent cancer Present | Negatives | true |
| Recurrent component | Negatives | true |
| Recurrent Disease absent | Negatives | false |
| Recurrent Disease Evident | Negatives | true |
| Recurrent Disease is absent | Negatives | false |
| Recurrent disease is not Present | Negatives | false |
| Recurrent disease not Present | Negatives | false |
| Recurrent Disease Present | Negatives | true |
| Recurrent malignancy | Negatives | true |
| Recurrent malignancy absent | Negatives | false |
| Recurrent malignancy Evident | Negatives | true |
| Recurrent malignancy not Present | Negatives | false |
| Recurrent malignancy Present | Negatives | true |
| Recurrent tumour | Negatives | true |
| Recurrent tumour absent | Negatives | false |
| Recurrent tumour Evident | Negatives | true |
| Recurrent tumour not Present | Negatives | false |
| Recurrent tumour Present | Negatives | true |

## *ResidualDisease.lst*

| **Value** | **Feature 1** | **Value 1** |
| --- | --- | --- |
| Evidence of Residual cancer | Negatives | true |
| Evidence of Residual Disease | Negatives | true |
| Evidence of Residual malignancy | Negatives | true |
| Evidence of Residual tumour | Negatives | true |
| Presence of cancer disease | Negatives | true |
| Presence of malignant disease | Negatives | true |
| Presence of residual disease | Negatives | true |
| Presence of tumour disease | Negatives | true |
| Residual | Negatives | true |
| Residual/recurrent tumour | Negatives | true |
| Residual cancer | Negatives | true |
| Residual cancer absent | Negatives | false |
| Residual cancer Evident | Negatives | true |
| Residual cancer not Present | Negatives | false |
| Residual cancer Present | Negatives | true |
| Residual component | Negatives | true |
| Residual Disease absent | Negatives | false |
| Residual Disease Evident | Negatives | true |
| Residual Disease is absent | Negatives | false |
| Residual disease is not Present | Negatives | false |
| Residual disease not Present | Negatives | false |
| Residual Disease Present | Negatives | true |
| Residual malignancy | Negatives | true |
| Residual malignancy absent | Negatives | false |
| Residual malignancy Evident | Negatives | true |
| Residual malignancy not Present | Negatives | false |
| Residual malignancy Present | Negatives | true |
| Residual tumour | Negatives | true |
| Residual tumour absent | Negatives | false |
| Residual tumour Evident | Negatives | true |
| Residual tumour not Present | Negatives | false |
| Residual tumour Present | Negatives | true |

## *SingleMargin.lst*

| **Value** |
| --- |
| Resection margin |
| Resection margins |

## *Specimen.lst*

| **Value** |
| --- |
| Disc of skin |
| Disc of skin measuring |
| Ellipse of skin |
| Ellipse of skin measuring |
| Fragment |
| Fragment measuring |
| Hourglass shaped |
| Hourglass shaped lesion |
| Marker stich |
| Marker stich measuring |
| Marker suture |
| Marker suture measuring |
| MS |
| o' clock |
| o' clock measuring |
| Portion |
| Skin |
| Skin shave |
| Skin shaves |
| Skin shaves, bigger |
| Skin shaves, biggest |
| Skin shaves, larger |
| Skin shaves, largest |
| Stitch |
| Stitch measuring |
| Suture |
| Suture measuring |
| Sutures |

## *StageTrigger.lst*

| **Value** |
| --- |
| AJCC7 |
| AJCC8 |
| Pathological (p) stage |
| Pathological stage |
| Stage |
| Staging |
| T,N,M |
| TNM |
| TNM (7th ed) |
| TNM (7th edition) |
| TNM (8th ed) |
| TNM (8th edition) |
| TNM 7th ed |
| TNM 7th edition |
| TNM 8th ed |
| TNM 8th edition |
| TNM pathological (p) stage |
| TNM pathological stage |

## *SupplementalExcisionTypeOutcome.lst*

| **Value** | **Feature 1** | **Value 1** |
| --- | --- | --- |
| Evidence of malignancy | Negatives | true |
| Evidence of tumour | Negatives | true |
| Malignancy absent | Negatives | false |
| Malignancy evident | Negatives | true |
| Malignancy not Present | Negatives | false |
| Malignancy Present | Negatives | true |
| Malignancy seen | Negatives | true |
| Malignant disease absent | Negatives | false |
| Malignant disease evident | Negatives | true |
| Malignant disease not Present | Negatives | false |
| Malignant disease Present | Negatives | true |
| Malignant disease seen | Negatives | true |
| Presence of Malignancy | Negatives | true |
| Presence of Malignant disease | Negatives | true |
| Presence of tumour | Negatives | true |
| Tumour absent | Negatives | false |
| Tumour evident | Negatives | true |
| Tumour not Present | Negatives | false |
| Tumour Present | Negatives | true |
| Tumour seen | Negatives | true |

## *Thickness.lst*

| **Value** | **Feature 1** | **Value 1** |
| --- | --- | --- |
| Depth |  |  |
| Maximum distance from epidermis |  |  |
| Maximum distance from the epidermis |  |  |
| Maximum distance to epidermis |  |  |
| Maximum distance to the epidermis |  |  |
| Thick | Position | Post |
| Thickness |  |  |

## *Ulcerated.lst*

| **Value** | **Feature 1** | **Value 1** |
| --- | --- | --- |
| Evidence of ulceration | Negatives | true |
| Nonulcerated | Negatives | false |
| Non ulcerated | Negatives | false |
| Non -ulcerated | Negatives | false |
| Non - ulcerated | Negatives | false |
| Non-ulcerated | Negatives | false |
| Non- ulcerated | Negatives | false |
| Presence of ulceration | Negatives | true |
| Ulcer |  |  |
| Ulcerated |  |  |
| Ulcerated ? | Negatives | true |
| Ulcerated Area | Negatives | true |
| Ulcerated Area | Negatives | true |
| Ulcerated Areas | Negatives | true |
| Ulcerated Areas | Negatives | true |
| Ulcerated Irregular area | Negatives | true |
| Ulcerated Irregular area | Negatives | true |
| Ulcerated Irregular areas | Negatives | true |
| Ulcerated Irregular areas | Negatives | true |
| Ulcerated Irregular surface | Negatives | true |
| Ulcerated Irregular surface | Negatives | true |
| Ulcerated lesion | Negatives | true |
| Ulcerated Lesion | Negatives | true |
| Ulcerated Lesion | Negatives | true |
| Ulcerated Lesions | Negatives | true |
| Ulcerated Lesions | Negatives | true |
| Ulcerated Nodule | Negatives | true |
| Ulcerated Nodule | Negatives | true |
| Ulcerated Nodules | Negatives | true |
| Ulcerated Nodules | Negatives | true |
| Ulcerated Plaque | Negatives | true |
| Ulcerated Plaque | Negatives | true |
| Ulcerated Plaques | Negatives | true |
| Ulcerated Plaques | Negatives | true |
| Ulcerated Surface | Negatives | true |
| Ulcerated Surface | Negatives | true |
| Ulcerated Surfaces | Negatives | true |
| Ulcerated Surfaces | Negatives | true |
| Ulcerating |  |  |
| Ulcerating ? | Negatives | true |
| Ulcerating lesion | Negatives | true |
| Ulceration |  |  |
| Ulceration ? | Negatives | true |
| Ulceration evident | Negatives | true |
| Ulceration of lesion | Negatives | true |
| Ulceration present | Negatives | true |
| Ulceration present | Negatives | true |
| With an ulcerating | Negatives | true |
| With a ulcerating | Negatives | true |
| With ulceration | Negatives | true |

## *UlceratedTrigger.lst*

| **Value** |
| --- |
| With |
| With a |
| With an |

## *UpperOrLower.lst*

| **Value** | **Feature 1** | **Value 1** |
| --- | --- | --- |
| Lower | UpperOrLower | Lower |
| Upper | UpperOrLower | Upper |

# **Blocks**

## *AdditionalSentenceBreak.lst*

| **Value** |
| --- |
| - |
| – |
| ' |
| " |

## *FrozenSectionBlocks.lst*

| **Value** |
| --- |
| Paraffin section |
| Paraffin sections |

## *LocalisationSkipTerms.lst*

| **Value** |
| --- |
| Area |
| Region |

# **Document details**

## *AccessionNumber.lst*

| **Value** |
| --- |
| accessionno |
| AccessionNo |
| accession no |
| Accession No |
| accessionnumber |
| AccessionNumber |
| accession number |
| Accession Number |

## *Excisiondate_trigger.lst*

| **Value** |
| --- |
| Date/Time Collected |
| DateandTime Collected |
| Date and Time Collected |

## *Reported_triggers.lst*

| **Value** | **Feature 1** | **Value 1** |
| --- | --- | --- |
| Authorised | Within | True |
| Reported and authorised | Within | False |
| Reported by | Within | True |

## *Reporter.lst*

| **Value** | **Feature 1** | **Value 1** |
| --- | --- | --- |
| A. Dawson | Name | AllanDawson |
| A. Finall | Name | AlisonFinall |
| A Dawson | Name | AllanDawson |
| A Finall | Name | AlisonFinall |
| Alison Finall | Name | AlisonFinall |
| Allan Dawson | Name | AllanDawson |
| D. Parker | Name | DannyParker |
| D. Parker | Name | DannyParker |
| D. W. Williams | Name | DavidWilliams |
| D. Williams | Name | DavidWilliams |
| D. W Williams | Name | DavidWilliams |
| Danny Parker | Name | DannyParker |
| Danny Parker | Name | DannyParker |
| David Williams | Name | DavidWilliams |
| D Parker | Name | DannyParker |
| D Parker | Name | DannyParker |
| D W. Williams | Name | DavidWilliams |
| D Williams | Name | DavidWilliams |
| DW Williams | Name | DavidWilliams |
| D W Williams | Name | DavidWilliams |
| G. Leopold | Name | GarethLeopold |
| Gareth Leopold | Name | GarethLeopold |
| G Leopold | Name | GarethLeopold |
| L. Semkin | Name | LeonidSemkin |
| Leonid Semkin | Name | LeonidSemkin |
| L Semkin | Name | LeonidSemkin |
| M. Brotto | Name | MaurizioBrotto |
| M. Cotter | Name | MargaretCotter |
| Margaret Cotter | Name | MargaretCotter |
| Maurizio Brotto | Name | MaurizioBrotto |
| M Brotto | Name | MaurizioBrotto |
| M Cotter | Name | MargaretCotter |
| N. Burke | Name | NadineBurke |
| N. Tofazzal | Name | NasimaTofazzal |
| N. W. Williams | Name | NamorWilliams |
| N. Williams | Name | NamorWilliams |
| Nadine Burke | Name | NadineBurke |
| Namor Williams | Name | NamorWilliams |
| Namor Wyn Williams | Name | NamorWilliams |
| Nasima Tofazzal | Name | NasimaTofazzal |
| N Burke | Name | NadineBurke |
| N Tofazzal | Name | NasimaTofazzal |
| N Williams | Name | NamorWilliams |
| N W Williams | Name | NamorWilliams |
| O. Kozyar | Name | OlexandraKozyar |
| O Kozyar | Name | OlexandraKozyar |
| Olexandra Kozyar | Name | OlexandraKozyar |
| P. Davis | Name | PeterDavis |
| P. Davis | Name | PeterDavis |
| P. Griffiths | Name | PaulGriffiths |
| Paul Griffiths | Name | PaulGriffiths |
| P Davis | Name | PeterDavis |
| P Davis | Name | PeterDavis |
| Peter Davies | Name | PeterDavis |
| Peter Davis | Name | PeterDavis |
| Peter Davis | Name | PeterDavis |
| P Griffiths | Name | PaulGriffiths |
| R. Trefor | Name | RhiannonTrefor |
| Rhiannon Trefor | Name | RhiannonTrefor |
| R Trefor | Name | RhiannonTrefor |
| S. Howarth | Name | SusannahHowarth |
| S. Ng | Name | SelwynNg |
| S. Roberts | Name | ShaunRoberts |
| Selwyn Ng | Name | SelwynNg |
| Shaun Roberts | Name | ShaunRoberts |
| S Howarth | Name | SusannahHowarth |
| S Ng | Name | SelwynNg |
| S Roberts | Name | ShaunRoberts |
| Susannah Howarth | Name | SusannahHowarth |
| Susanna Howarth | Name | SusannahHowarth |
| Suzie Howarth | Name | SusannahHowarth |
| T. Elazzabi | Name | TawfikElazzabi |
| Tawfik Elazzabi | Name | TawfikElazzabi |
| T Elazzabi | Name | TawfikElazzabi |
| V. Shah | Name | VarshaShah |
| Varsha Shah | Name | VarshaShah |
| V Shah | Name | VarshaShah |

## *Requestor_triggers.lst*

| **Value** |
| --- |
| Requestor |

## *Requestor.lst*

| **Value** | **Feature 1** | **Value 1** | **Feature 2** |
| --- | --- | --- | --- |
| A.Emam | Name | AhmedEmam | Speciality=Plastics |
| A. Emam | Name | AhmedEmam | Speciality=Plastics |
| A.Ghattaura | Name | AmarGhattaura | Speciality=Plastics |
| A. Ghattaura | Name | AmarGhattaura | Speciality=Plastics |
| A.Mughal | Name | AvadMughal | Speciality=Dermatology |
| A. Mughal | Name | AvadMughal | Speciality=Dermatology |
| A Emam | Name | AhmedEmam | Speciality=Plastics |
| A Ghattaura | Name | AmarGhattaura | Speciality=Plastics |
| Ahmed Emam | Name | AhmedEmam | Speciality=Plastics |
| Amar Ghattaura | Name | AmarGhattaura | Speciality=Plastics |
| A Mughal | Name | AvadMughal | Speciality=Dermatology |
| Avad Mughal | Name | AvadMughal | Speciality=Dermatology |
| B.Dixon | Name | BillDixon | Speciality=Plastics |
| B. Dixon | Name | BillDixon | Speciality=Plastics |
| B Dixon | Name | BillDixon | Speciality=Plastics |
| Bill Dixon | Name | BillDixon | Speciality=Plastics |
| D.Boyce | Name | DeanBoyce | Speciality=Plastics |
| D. Boyce | Name | DeanBoyce | Speciality=Plastics |
| D.Nguyen | Name | DaiNguyen | Speciality=Plastics |
| Dai Nguyen | Name | DaiNguyen | Speciality=Plastics |
| D Boyce | Name | DeanBoyce | Speciality=Plastics |
| Dean Boyce | Name | DeanBoyce | Speciality=Plastics |
| D Nguyen | Name | DaiNguyen | Speciality=Plastics |
| E.Azzopardi | Name | ErnestAzzopardi | Speciality=Plastics |
| E. Azzopardi | Name | ErnestAzzopardi | Speciality=Plastics |
| E Azzopardi | Name | ErnestAzzopardi | Speciality=Plastics |
| Ernest Azzopardi | Name | ErnestAzzopardi | Speciality=Plastics |
| F.Schreuder | Name | FredSchreuder | Speciality=Plastics |
| F. Schreuder | Name | FredSchreuder | Speciality=Plastics |
| Fredrick Schreuder | Name | Fred Schreuder | Speciality=Plastics |
| Fred Schreuder | Name | Fred Schreuder | Speciality=Plastics |
| F Schreuder | Name | FredSchreuder | Speciality=Plastics |
| H.Dafydd | Name | HywelDafydd | Speciality=Plastics |
| H. Dafydd | Name | HywelDafydd | Speciality=Plastics |
| H.Laing | Name | HamishLaing | Speciality=Plastics |
| H. Laing | Name | HamishLaing | Speciality=Plastics |
| Hamish Laing | Name | HamishLaing | Speciality=Plastics |
| H Dafydd | Name | HywelDafydd | Speciality=Plastics |
| H Laing | Name | HamishLaing | Speciality=Plastics |
| Hywel Dafydd | Name | HywelDafydd | Speciality=Plastics |
| I.Josty | Name | IainJosty | Speciality=Plastics |
| I. Josty | Name | IainJosty | Speciality=Plastics |
| I.Whitaker | Name | IainWhitaker | Speciality=Plastics |
| I. Whitaker | Name | IainWhitaker | Speciality=Plastics |
| Iain Josty | Name | IainJosty | Speciality=Plastics |
| Iain Whitaker | Name | IainWhitaker | Speciality=Plastics |
| I Josty | Name | IainJosty | Speciality=Plastics |
| I Whitaker | Name | IainWhitaker | Speciality=Plastics |
| J.Cubitt | Name | JonathanCubitt | Speciality=Plastics |
| J. Cubitt | Name | JonathanCubitt | Speciality=Plastics |
| J.Warbrick-Smith | Name | JamesWSmith | Speciality=Plastics |
| J. Warbrick-Smith | Name | JamesWSmith | Speciality=Plastics |
| J.Yarrow | Name | JeremyYarrow | Speciality=Plastics |
| J. Yarrow | Name | JeremyYarrow | Speciality=Plastics |
| James Warbrick-Smith | Name | JamesWSmith | Speciality=Plastics |
| J Cubitt | Name | JonathanCubitt | Speciality=Plastics |
| Jeremy Yarrow | Name | JeremyYarrow | Speciality=Plastics |
| Jonathan Cubitt | Name | JonathanCubitt | Speciality=Plastics |
| Jon Cubitt | Name | JonathanCubitt | Speciality=Plastics |
| J Smith | Name | JamesWSmith | Speciality=Plastics |
| J Yarrow | Name | JeremyYarrow | Speciality=Plastics |
| L.Harry | Name | LozHarry | Speciality=Plastics |
| L. Harry | Name | LozHarry | Speciality=Plastics |
| L.Hiew | Name | LeongHiew | Speciality=Plastics |
| L. Hiew | Name | LeongHiew | Speciality=Plastics |
| L.Tang | Name | LydiaTang | Speciality=Plastics |
| L. Tang | Name | LydiaTang | Speciality=Plastics |
| Leong Hiew | Name | LeongHiew | Speciality=Plastics |
| L Harry | Name | LozHarry | Speciality=Plastics |
| L Hiew | Name | LeongHiew | Speciality=Plastics |
| Lorraine Harry | Name | LozHarry | Speciality=Plastics |
| Loz Harry | Name | LozHarry | Speciality=Plastics |
| L Tang | Name | LydiaTang | Speciality=Plastics |
| Lydia Tang | Name | LydiaTang | Speciality=Plastics |
| Lydia Tang | Name | LydiaTang | Speciality=Plastics |
| M.Cooper | Name | MarkCooper | Speciality=Plastics |
| M. Cooper | Name | MarkCooper | Speciality=Plastics |
| M.Haj-Basheer | Name | MohammedHBasheer | Speciality=Plastics |
| M. Haj-Basheer | Name | MohammedHBasheer | Speciality=Plastics |
| M.Javaid | Name | MuhammadJavaid | Speciality=Plastics |
| M. Javaid | Name | MuhammadJavaid | Speciality=Plastics |
| M.Murison | Name | MaxMurison | Speciality=Plastics |
| M. Murison | Name | MaxMurison | Speciality=Plastics |
| MarkCooper | Name | MarkCooper | Speciality=Plastics |
| MaxMurison | Name | MaxMurison | Speciality=Plastics |
| M Cooper | Name | MarkCooper | Speciality=Plastics |
| M Haj-Basheer | Name | MohammedHBasheer | Speciality=Plastics |
| M Javaid | Name | MuhammadJavaid | Speciality=Plastics |
| M Murison | Name | MaxMurison | Speciality=Plastics |
| Mohammed Haj-Basheer | Name | MohammedHBasheer | Speciality=Plastics |
| Muhammad Javaid | Name | MuhammadJavaid | Speciality=Plastics |
| N.Marsden | Name | NickMarsden | Speciality=Plastics |
| N. Marsden | Name | NickMarsden | Speciality=Plastics |
| N.Wilson-Jones | Name | NicholasWJones | Speciality=Plastics |
| N. Wilson-Jones | Name | NicholasWJones | Speciality=Plastics |
| Nicholas Marsden | Name | NickMarsden | Speciality=Plastics |
| Nicholas Wilson-Jones | Name | NicholasWJones | Speciality=Plastics |
| Nick Marsden | Name | NickMarsden | Speciality=Plastics |
| Nick Wilson-Jones | Name | NicholasWJones | Speciality=Plastics |
| N Marsden | Name | NickMarsden | Speciality=Plastics |
| N Wilson-Jones | Name | NicholasWJones | Speciality=Plastics |
| P.Drew | Name | PeterDrew | Speciality=Plastics |
| P. Drew | Name | PeterDrew | Speciality=Plastics |
| P Drew | Name | PeterDrew | Speciality=Plastics |
| Peter Drew | Name | PeterDrew | Speciality=Plastics |
| Prof.I.Whitaker | Name | IainWhitaker | Speciality=Plastics |
| Prof.I. Whitaker | Name | IainWhitaker | Speciality=Plastics |
| Prof. I.Whitaker | Name | IainWhitaker | Speciality=Plastics |
| Prof. I. Whitaker | Name | IainWhitaker | Speciality=Plastics |
| R.Duncan | Name | RobDuncan | Speciality=Plastics |
| R. Duncan | Name | RobDuncan | Speciality=Plastics |
| R.Karoo | Name | RichardKaroo | Speciality=Plastics |
| R. Karoo | Name | RichardKaroo | Speciality=Plastics |
| R. Nguyen | Name | DaiNguyen | Speciality=Plastics |
| R Duncan | Name | RobDuncan | Speciality=Plastics |
| Richard Karoo | Name | RichardKaroo | Speciality=Plastics |
| R Karoo | Name | RichardKaroo | Speciality=Plastics |
| Rob Duncan | Name | RobDuncan | Speciality=Plastics |
| Robert Duncan | Name | RobDuncan | Speciality=Plastics |
| S.Blackford | Name | SharonBlackford | Speciality=Dermatology |
| S. Blackford | Name | SharonBlackford | Speciality=Dermatology |
| S.Hemmington-Gorse | Name | SarahHGorse | Speciality=Plastics |
| S. Hemmington-Gorse | Name | SarahHGorse | Speciality=Plastics |
| S.Pope-Jones | Name | SophiePJones | Speciality=Plastics |
| S. Pope-Jones | Name | SophiePJones | Speciality=Plastics |
| S.Whitaker | Name | SairanWhitaker | Speciality=Dermatology |
| S. Whitaker | Name | SairanWhitaker | Speciality=Dermatology |
| Sairan Whitaker | Name | SairanWhitaker | Speciality=Dermatology |
| Sarah Hemmington-Gorse | Name | SarahHGorse | Speciality=Plastics |
| S Blackford | Name | SharonBlackford | Speciality=Dermatology |
| Sharon Blackford | Name | SharonBlackford | Speciality=Dermatology |
| S Hemmington-Gorse | Name | SarahHGorse | Speciality=Plastics |
| Sophie Pope-Jones | Name | SophiePJones | Speciality=Plastics |
| S Pope-Jones | Name | SophiePJones | Speciality=Plastics |
| S Whitaker | Name | SairanWhitaker | Speciality=Dermatology |
| T.Bragg | Name | ThomasBragg | Speciality=Plastics |
| T. Bragg | Name | ThomasBragg | Speciality=Plastics |
| T.O’Neill | Name | TomasONeill | Speciality=Plastics |
| T. O’Neill | Name | TomasONeill | Speciality=Plastics |
| T.Potokar | Name | TomPotokar | Speciality=Plastics |
| T. Potokar | Name | TomPotokar | Speciality=Plastics |
| T.Tickunas | Name | TomasTickunas | Speciality=Plastics |
| T. Tickunas | Name | TomasTickunas | Speciality=Plastics |
| T Bragg | Name | ThomasBragg | Speciality=Plastics |
| Thomas Bragg | Name | ThomasBragg | Speciality=Plastics |
| Thomas Potokar | Name | TomPotokar | Speciality=Plastics |
| Tomas O’Neill | Name | TomasONeill | Speciality=Plastics |
| Tomas Tickunas | Name | TomasTickunas | Speciality=Plastics |
| Tom Bragg | Name | ThomasBragg | Speciality=Plastics |
| Tom Potokar | Name | TomPotokar | Speciality=Plastics |
| T ONeill | Name | TomasONeill | Speciality=Plastics |
| T Potokar | Name | TomPotokar | Speciality=Plastics |
| T Tickunas | Name | TomasTickunas | Speciality=Plastics |

## *SupplementaryReport_trigger.lst*

| **Value** |
| --- |
| Addendum |
| Additional report |
| Amended report |
| Amendment |
| Ammended report |
| Ammendment |
| Supplemental report |
| Supplementary report |

## *Titles.lst*

| **Value** |
| --- |
| Doctor |
| Dr |
| Miss |
| Mr |
| Ms |
| Prof |
| Professor |

# **Paragraph titles**

## *ParagraphTitles.lst*

| **Value** | **Feature 1** | **Value 1** |
| --- | --- | --- |
| Accession No | type | AccessionNumber |
| Accession Number | type | AccessionNumber |
| Addendum | type | SupplementaryReport |
| Additional report | type | SupplementaryReport |
| Amended report | type | SupplementaryReport |
| Amendment | type | SupplementaryReport |
| Ammended report | type | SupplementaryReport |
| Ammendment | type | SupplementaryReport |
| Clinical Details | type | ClinicalDetails |
| Macro Description | type | MacroDescription |
| Macroscopy | type | MacroDescription |
| Microscopy | type | Microscopy |
| Specimen received | type | SpecimensReceived |
| Specimen review | type | SupplementaryReport |
| Specimens received | type | SpecimensReceived |
| Supplemental report | type | SupplementaryReport |
| Supplementary report | type | SupplementaryReport |
| Supplementary Report | type | SupplementaryReport |
| Supplementary report: specimen review | type | SupplementaryReport |
| Technical | type | Technical |

# **Case sensitive**

## *CaseSensitiveDiagnosis.lst*

| **Value** | **Feature 1** | **Value 1** | **Feature 2** | **Value 2** |
| --- | --- | --- | --- | --- |
| AFX | CUI | C0346053 | CancerType | OtherCancerous |
| AK | CUI | C0006079 | Type | OtherInSitu |
| BCC | CUI | C0007117 | CancerType | BasalCellCarcinoma |
| DFSP | CUI | C0392784 | CancerType | OtherCancerous |
| ESH | CUI | C3840252 | CancerType | OtherIntermediate |
| ES H | CUI | C3840252 | CancerType | OtherIntermediate |
| ES-H | CUI | C3840252 | CancerType | OtherIntermediate |
| LMM | CUI | C2739810 | CancerType | OtherCancerous |
| MM | CUI | C0151779 | CancerType | OtherCancerous |
| NM | CUI | C0334424 | CancerType | OtherCancerous |
| NMM | CUI | C0334424 | CancerType | OtherCancerous |
| PHE | CUI | C3840252 | CancerType | OtherIntermediate |
| SCC | CUI | C0553723 | CancerType | OtherCancerous |
| SSM | CUI | C0334438 | CancerType | OtherCancerous |
| SSM in situ | CUI | C0854696 | CancerType | OtherInSitu |
| SSMM | CUI | C0334438 | CancerType | OtherCancerous |
| SSMM in situ | CUI | C0854696 | CancerType | OtherInSitu |
