# Supplementary material for: Development and validation of an automated basal cell carcinoma histopathology information extraction system using natural language processing
Source: Front Surg. 2022 Aug 24;9:870494. doi: 10.3389/fsurg.2022.870494 (PMC9683031; doi:10.3389/fsurg.2022.870494)
Supplement: Supplementary file 6 [file Datasheet3.docx]

*Calculating the number of documents needed for a gold standard validation corpus*

Assuming we have a corpus of 1000 documents and a word appears in 3 documents the probability that at least one document has the word when randomly selecting 100 documents is shown by Equation 1. Or more generally, assuming we have a corpus of  documents and a word appears in documents the probability that at least one document has the word when randomly selecting  documents is shown by Equation 2. By setting  = 1000 and calculating the probability for various  and  a plot of capture probability vs document frequency can be generated (Figure 1). This plot shows the capture probability of a word with a given document frequency in a 1000 document corpus. It is not surprising that the capture probability of a word with a smaller document frequency is lower. We defined a valid token for this process as any sequence of alphanumeric characters, beginning with a letter and occurring between spaces, slashes, brackets, braces, parentheses, quotation marks or punctuation marks. To capture 95% of tokens of document frequency = 1, 950 documents would be needed. You need less than 500 documents if the document frequency = 5 and less than 300 if the document frequency = 10. Note that a document frequency = 10 means the word only appears in 1% of the documents in the corpus. We then calculated the capture probability of all valid and relevant tokens manually picked by expert skin cancer clinicians from the working corpus. Assuming we have  unique tokens and knowing the capture probability of each token the percentage of tokens being captured can be shown by Equation 3. We then calculated the document frequency of valid and relevant tokens in the corpus. By only keeping words with a minimum length, small token sizes with a low document frequency can be eliminated (Figure 2). We decided to use tokens with nine characters as a cut off for this task (Figure 3). We also set a minimum document frequency of 5 or above as we deemed that rare tokens would not be used in our JAPE rules.

**Figure 1:** Plot of capture probability versus document frequency.


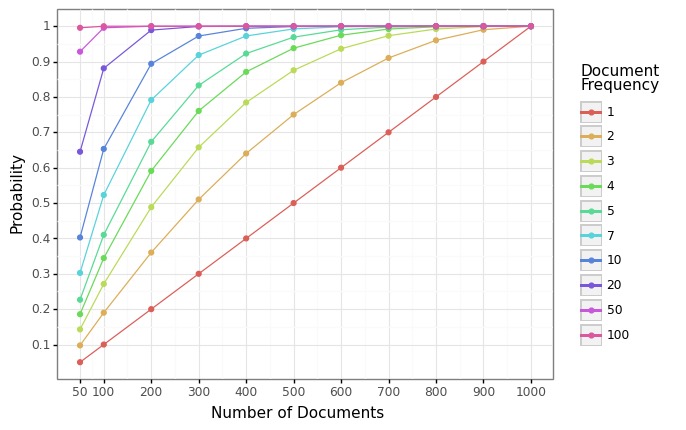


**Figure 2:** Distribution of document frequency in the working corpus.


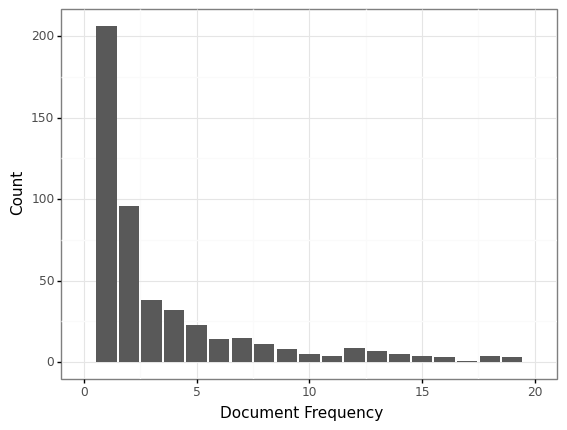


**Figure 3:** Plot demonstrating the aggregate capture probability when keeping tokens appearing in ≥ 5 histopathology reports.


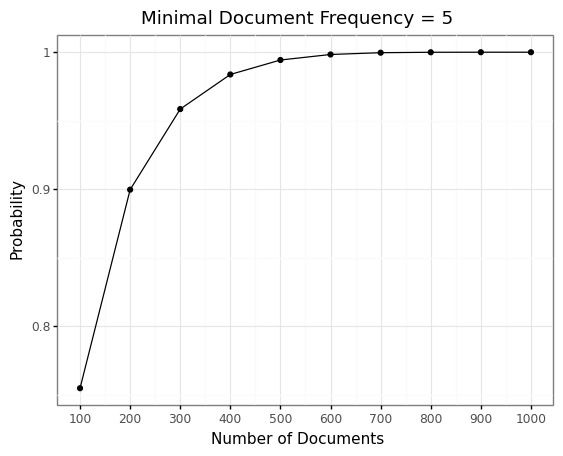


**Equation 1:** Probability that at least one document has the word when randomly selecting 100 documents.

**Equation 2:** The probability that at least one document has the word when randomly selecting documents.

**Equation 3:** Percentage probability of token being captured.
